# Efficient chemical fixation and defixation cycle of carbon dioxide under ambient conditions

**DOI:** 10.1038/s41598-020-71761-w

**Published:** 2020-09-25

**Authors:** Saumen Hajra, Anurag Biswas

**Affiliations:** Center of Biomedical Research, Sanjay Gandhi Post-Graduate Institute of Medical Sciences Campus, Raebareli Road, Lucknow, 226014 India

**Keywords:** Climate sciences, Chemistry

## Abstract

Chemical fixation of CO_2_ as a C1 feedstock for producing value-added products is an important post-combustion technology reducing the CO_2_ emission. As it is an irreversible process, not considered for the CO_2_ capture and release. Overall, these chemical transformations also do not help to mitigate global warming, as the energy consumed in different forms is much higher than the amount of CO_2_ fixed by chemical reactions. Here we describe the development of re-generable chemical fixation of CO_2_ by spiroaziridine oxindole, where CO_2_ is captured (chemical fixation) under catalyst-free condition at room temperature both in aqueous and non-aqueous medium even directly from the slow stream of flue gas producing regioselectively spirooxazolidinyl oxindoles, a potential drug. The CO_2_-adduct is reversed back to the spiroaziridine releasing CO_2_ under mild conditions. Further both the fixation-defixation of CO_2_ can be repeated under near ambient conditions for several cycles in a single loop using a recyclable reagent.

## Introduction

Means of viable development, typically relying on more sensible resource management, is a conceit challenge in front of modern human society. Sustainability level in recent economic growth requires a massive improvement as it is far from an adequate level. According to the data released by Intergovernmental Panel on Climate Change (IPPC 2018), global surface temperature has mounted by approximately 1.5 °C from 1880 to 2018, which is a phenomenon caused by anthropogenic activities, predominantly greenhouse gases like CO_2_ emissions from fossil carbon to accomplish the escalating energy demand. Under this circumstances, melting of thousand years old glaciers, desertification of fertile land, rise in ocean water level and acidification of ocean water had caused enormous detriment to diverse ecological environment^1^. Scientific and technical advancements to curve atmospheric CO_2_ concentration via limiting industrial emission and use CO_2_ as an alternative fuel source in the renewable energy sector, had been a recurrent course of study for past few years^2–5^. The reduction of CO_2_ can be considered as a typical cohesive technology to rise artificial efficiency in producing various valuable hydrocarbons like formic acid, methanol, methane, and C2–C4 olefins^6–12^. Several fascinating integrated protocols have freshly been reported for hydrothermal and photochemical CO_2_ reduction, e.g., metal/metal oxide redox reaction based solar two-step water-splitting thermochemical cycle for CO_2_ reduction via hydrogen generation^13–22^. Alongside, chemical fixation of CO_2_ has gained substantial importance in synthetic chemistry because CO_2_ could be used as a benign, abundant, inexpensive, and renewable C1 reserve to yield a variety of value-added chemicals e.g. esters, amides, aldehydes, carboxylic acids, alcohols, organic carbonates and 2-oxazolidinones, etc^23–26^. In particular, synthesis of therapeutically cherished and synthetically convenient five-membered cyclic urethanes such as oxazolidinones via cycloaddition of CO_2_ with aziridines has become one of the most promising approaches in this area, because this process possess 100% atom efficiency, which exactly matches one of the most substantial criteria of green chemistry^27–31^. Despite being an admirable strategy to chemically capture and recycle CO_2_, most of these protocols suffer from high energy demand and utilize costly catalysts/ionic liquids to achieve ambient or near ambient condition for CO_2_ fixation, even from highly enriched CO_2_ source^32–40^. However, emissions from thermal power plants contain numerous gaseous components like SO_2_, NO_2_ along with CO_2_^41^. In these context, post-combustion CO_2_ capture, release, and storage (CCS) had been the most abundantly used protocols for CO_2_ purification from industrial exhausts. Various strategies are being industrialized for capture, release and storage (CCS) of CO_2_ from gas streams, where gas–solid adsorption by metal–organic frameworks, gas–liquid chemi-absorption by amines and carbonation by quick/slacked lime are notable^42–46^. However, chemical fixation of CO_2_ from contaminated sources under mild conditions to produce industrially vibrant chemicals and products faces great defies because of two main reasons: (1) the high ionization potential (IP), and (2) the negative adiabatic electron affinity (EA) of carbon dioxide. Therefore, most of the reports use harsh reaction conditions to overcome the high thermodynamic stability and chemical inertness of carbon dioxide. Hence, the development of a cost-effective and robust protocol for CO_2_ capture, storage, and release in ambient conditions along with utility is highly desirable. Further, the chemical fixation is an irreversible process producing stable covalent compounds and thus, till now it could not be utilized for CO_2_ capture and release. It might be a potential CCS protocol as it would produce valuable chemicals, provided the chemical fixation and the defixation (release) done under ambient conditions, the latter is an unmet challenge. Herein, we report the first regenerable chemical fixation, where CO_2_ fixation by spiroaziridine oxindole under atmospheric pressure at rt (30 °C) without any catalyst producing stable spirooxazolidinone, a potential drug candidate^47–49^, further reversed back (defixation) to the spiroaziridine releasing CO_2_ under mild conditions. This fixation and defixation cycle can be repeated in a single loop for several times using a recyclable reagent.

## Results and discussion

### Uniqueness of spiroaziridine- and spirooxazolidinone oxindoles

**Figure 1:**
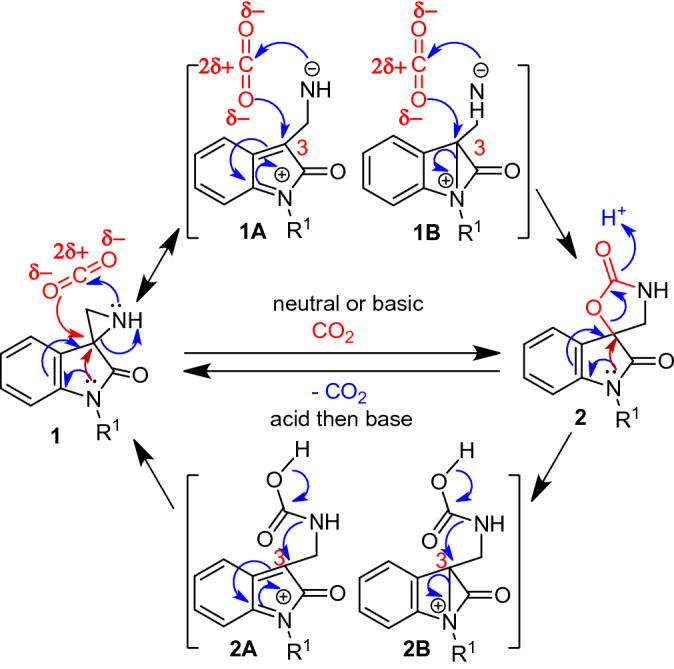
Proposed reactivity of spiroaziridine **1** and spirooxazolidinone **2** towards CO_2_ fixation and release.

CO_2_ is an overall non-polar molecule, but the presence of net partial charges [O^−δ^–C^+2δ^–O^δ^] makes its susceptibility to nucleophilic as well as electrophilic attack at carbon and oxygen, respectively. As a consequence, substrate such as epoxide and aziridine with both reactivity centres are suitable for the fixation of CO_2_^20,36–40^. However, all these require high pressure, -temperature and/or catalyst/additive. Designing substrate with tuned reactivity may lower the pressure and temperature for the chemical fixation of CO_2_ and may further facilitate the CO_2_ release. We envision that NH-free spiroaziridine oxindole **1** could be a suitable substrate with desired reactivity as the presence of oxindole unit may enhance the nucleophilicity of aziridine-nitrogen via an electron-donating resonance effect of nitrogen of oxindole unit and/or its anchimeric assistance (Fig. 1), simultaneously these may increase the electrophilicity of the C3 center of oxindole via resonance structure **1A** and/or the formation of intermediate **1B** under neutral or mild basic condition^50–52^. It is further envisioned that the presence of oxindole unit in spirooxazolidinone similarly will enable the release of CO_2_ under acidic conditions as shown in Fig. 1)^53,54^. More importantly, the spirooxazolidinoyl oxindole is a potential drug candidate^47–49^, so this CO_2_ fixation could be excellent and cheap method for its production.

### Optimization of auto-chemical fixation of CO_2_ by NH-free spiroaziridine 1a under ambient conditions

According to the presumption, we started our studies initially on synthesis of NH-free spiroaziridne oxindole **1a** and its reactivity towards CO_2_ under different conditions. We have developed a new and efficient method for the synthesis of NH-free spiro aziridine **1a** from easily available amino alcohol **3a** on successive treatment with chlorosulfonic acid (ClSO_3_H) in dioxane and aqueous KOH. The exclusive formation of NH-free spiroaziridine **1a** was detected by MS and NMR analysis. With great delight, when a slow stream of CO_2_ was passed through an aqueous dioxane solution of in situ synthesized spiroaziridine **1a** at rt, within 30 min it produced exclusively CO_2_ adduct, spiro oxazolidione **2a** in excellent yield (Table 1, entry 1) without any catalyst. This might be the first report of catalyst-free spontaneous chemical fixation of CO_2_ under ambient condition and also in aqueous medium. Instead of aqueous, solid KOH was also found to be suitable for the in situ synthesis of spiroaziridine **1a** and subsequent fixation of CO_2_, but it took a bit more time than the aqueous-dioxane (entry 2). The dioxane was the optimized solvent for both in situ spiroaziridine formation and the chemical fixation of CO_2_. NaOH instead of KOH is also equally effective for the synthesis of spiroaziridine and subsequent CO_2_ fixation (entries 3 and 4). Further, when in situ generated spiroaziridine was taken in ethyl acetate and treated with slow stream of CO_2_ in absence of any base, it also gave the CO_2_-adduct within 1.5 h in 69% isolated yield (entry 5). Thus it can be concluded that the chemical fixation of CO_2_ by spiroaziridine does not require base as a catalyst/promoter. Ultimately with our great delight, the auto-chemical fixation of CO_2_ was successful with 12.5% CO_2_ in N_2_ as well as a stimulated coal flue gas (12.5% CO_2_, 7.5% O_2_ and 80% N_2_) without any appreciable loss in the yield of the adduct (entries 6–8). These took longer reaction time, may be due to low concentration and retention of CO_2_ in solution.

**Table 1:** Optimization of in situ synthesis of spiroaziridine** 1a** and fixation of CO_2_.
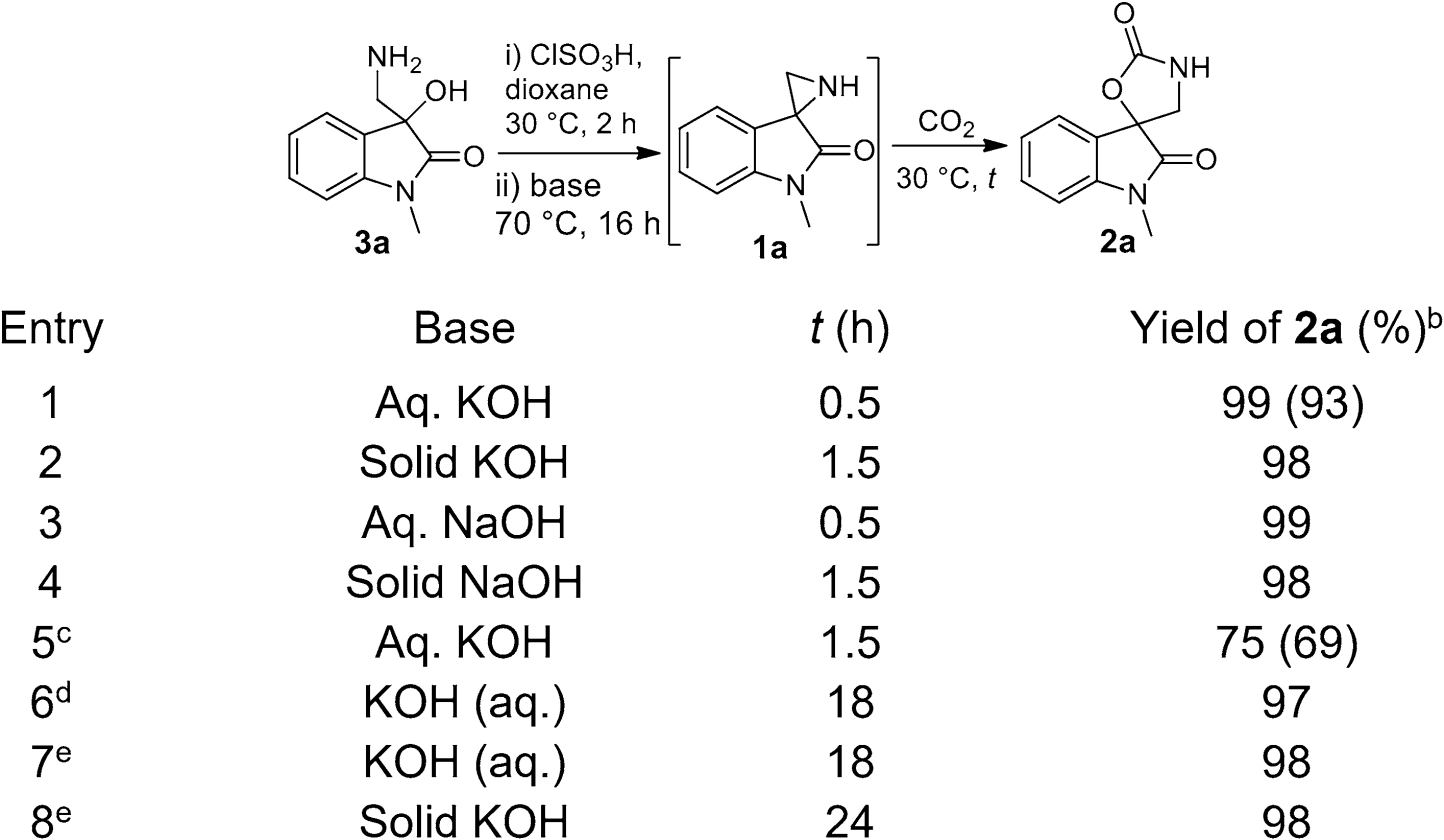

Entry	Base	*t* (h)	Yield of **2a** (%)^a^
1	Aq. KOH	0.5	99 (93)
2	Solid KOH	1.5	98
3	Aq. NaOH	0.5	99
4	Solid NaOH	1.5	98
5^b^	Aq. KOH	1.5	75 (69)
6^c^	KOH (aq.)	18	97
7^d^	KOH (aq.)	18	98
8^d^	Solid KOH	24	98

Chlorosulfonic acid (1.0 equiv.) was added slowly into the dioxane solution of **3a** (100 mg, 0.521 mmol) and stirred at 70 °C. Reaction mixture was basified and a slow stream of carbon dioxide was passed through the solution until complete consumption of **1a**.

^a^GC-yield is determined using naphthalene as internal standard; the value in parenthesis referred to the isolated yield.

^b^Spiroaziridine **1a** was extracted with ethyl acetate and treated with slow stream of CO_2_.

^c^12.5% CO_2_ gas in N_2_ was used as CO_2_ source.

^d^Stimulated flue gas (12.5% CO_2_, 7.5% O_2_ and 80% N_2_) was used as CO_2_ source.

### Defixation of CO_2_ at near ambient conditions

We next sought to explore the possibility to regenerate the spiroaziridine via decarboxylation, which is an unmet challenge in CO_2_-chemical fixation. As per the presumption, the decarboxylation (CO_2_ release) was initiated with the reaction of spiroxazolidinone in the presence of different Brǿnsted acids and the subsequent treatment of base to regenerate the spiroaziridine and its regeneration was quantified with the further chemical fixation of CO_2_ leading to spirooxazolidione again. Both the CO_2_ defixation and the fixation were optimized in dioxane and briefly summarized in Table 2. The regeneration of spiroaziridine **1a** was detected when a dioxane solution of spirooxazolidinone was heated with triflic acid at 100 °C. The extend of formation of spiroaziridine was confirmed by its chemical fixation of CO_2_ and it gave only 24% yield of the resynthesized spirooxazolidinone **2a** (Table 2, entry 1). With our great delight, near quantitative formation of spiroaziridine **1a** was achieved, when the compound **2a** was heated only at 70 °C with HI followed by treatment with aqueous NaOH (Table 2, entry 4). This was revealed with the re-synthesis of spirooxazolidinone **2a** with 94% of isolated yield. HBr was also found to act on at 70 °C, but it took longer time with incomplete conversion (entry 6). Further, to avoid the cumbersome procedure for the preparation of dioxane-HX, we developed an efficient and handy reagent, NaI-phosphoric acid for the regeneration of spiroaziridine **1a** (entry 8) (“Supplementary material”).

**Table 2:** Optimization of reaction condition for defixation and subsequent fixation of CO_2_.
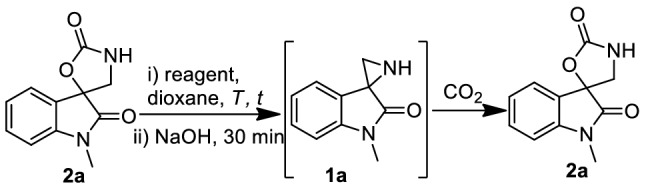

Entry	Reagent	*t* (h)	T (°C)	Conversion (%)^a^	Yield of **2a** (%)^b^
1	TfOH	12	100	> 99	24
2	HI	48	rt	NR	–
3	HI	48	50	45	–
4	HI	5	70	> 99	98 (94)
5	HBr	48	50/rt	NR	–
6	HBr	6	70	64	–
7	NaI-H_3_PO_4_	48	50	48	–
8	**NaI–H**_**3**_**PO**_**4**_	**5**	**70**	**> 99**	**98 (95)**
9	NaBr–H_3_PO_4_	48	70	NR	–

A dioxane solution of spiro-oxazolidone **2a** (100 mg, 0.46 mol) was heated under specified acidic conditions followed by treatment of base and then slow stream of CO_2_.

^a^Conversion of **2a** was determined by GC–MS analysis.

^b^GC-yield is determined using naphthalene as internal standard; the value in parenthesis referred to the isolated yield.

### Mechanism of CO_2_ defixation

**Figure 2:**
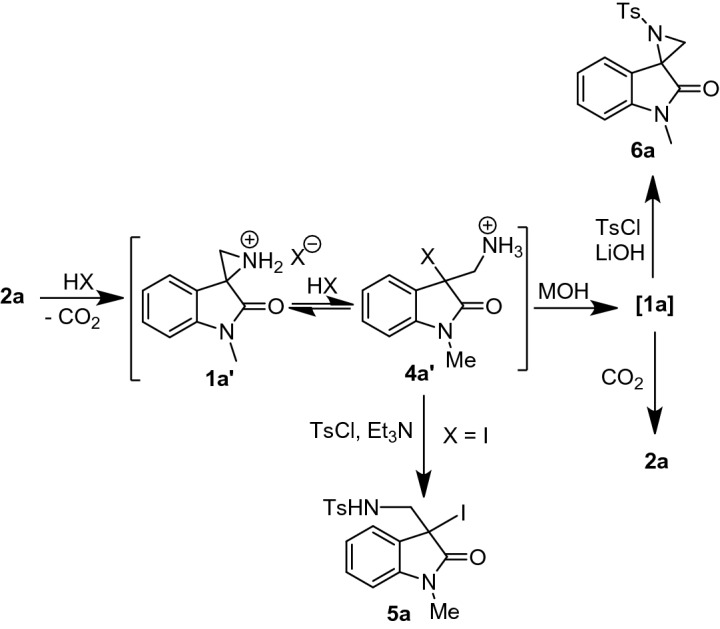
Intermediate compounds during defixation of CO_2_ from **2a.**

In TfOH mediated decarboxylation of **2a** (defixation of CO_2_), the formation of spiroaziridinium ion **1a′** was detected by MS analysis prior to the treatment with base. However, in case of HI or NaI-H_3_PO_4_, exclusive formation of intermediate compound 3-(aminomethyl)-3-iodooxindole **4a′** and no **1a′** was observed by MS analysis prior to the reaction with base. The intermediate iodo-amine **4a′** was isolated and identified as N-tosyl compound **5a** by MS and NMR analysis (Fig. 2). The intermediate compound **4a′** on treatment with base regenerated the spiroaziridine **1a**. Its in situ formation was confirmed by MS and NMR analysis and further isolated as N-tosyl spiroaziridine **6a**.

### Chemical fixation and defixation cycle of CO_2_

**Figure 3:**
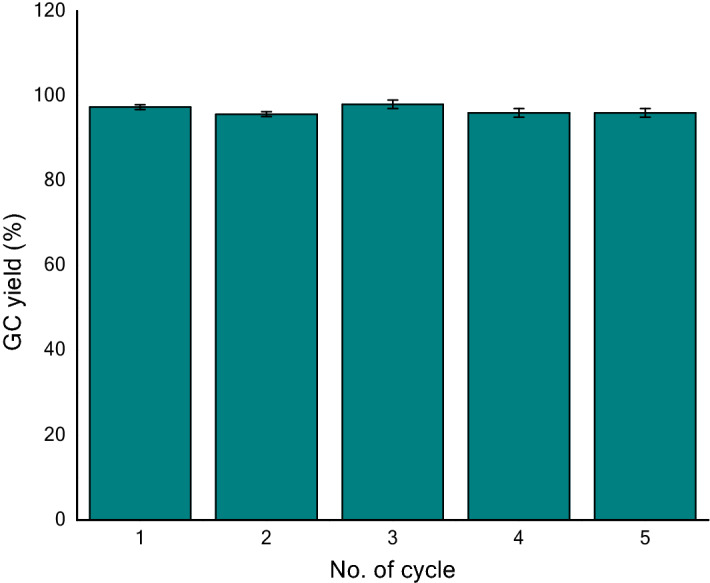
The recycling of spiroaziridine **1a** via re-synthesis of spirooxazolidinone **2a** (The yield in each cycle referred to the GC yield of resynthesized spirooxazolidinone **2a**; Standard deviation: cycle 1 and 2 = 0.58; cycle 3–5 = 1).

The defixation of CO_2_ at 70 °C and the subsequent fixation of CO_2_ at rt (25 °C) was repeated for five times through the isolation of regenerated spirooxazolidinone **2a** using solid NaOH. All the cycles required equal CO_2_-defixation and fixation time-scale and exhibited quantitative regeneration of **2a** (≥ 95%; Fig. 3). Further the CO_2_ defixation (at 70 °C) and the fixation (at rt) were successfully continued for consecutive five cycles in one-pot by treating with NaI-H_3_PO_4_ and solid NaOH. Excitingly the overall yield of spirooxazolidinone after five cycles was found to be excellent (overall GC yield 95% and isolated yield 90%).

**Figure 4:**
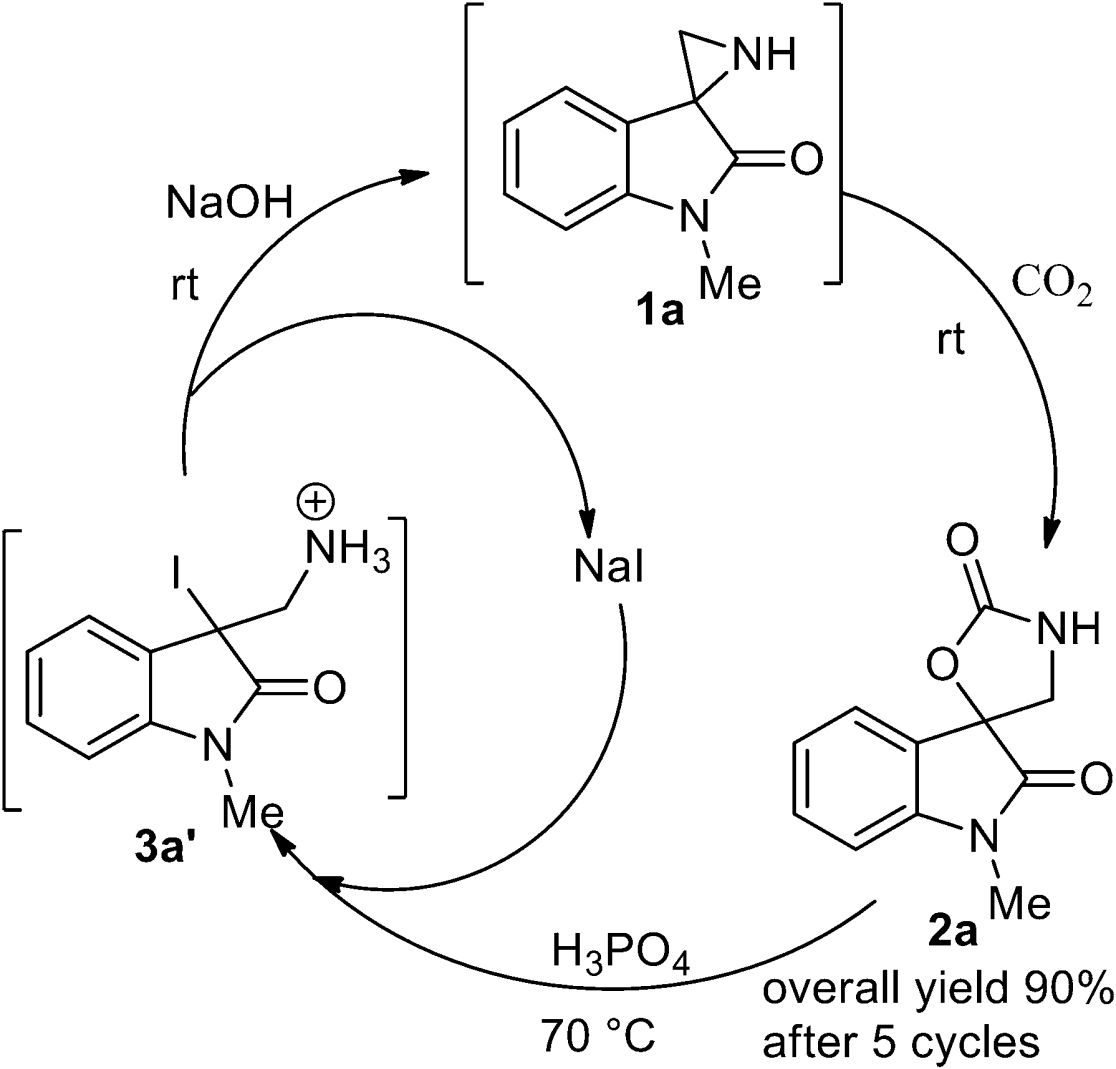
Fixation–defixation cycle of CO_2_ with recyclable NaI.

Again, if we deeply look into the chemical reactions involved during the release and capture of CO_2_ of the process, NaI supposed to regenerate after the treatment of **4a′**/**1a′** with NaOH. So, in principle, NaI may be reused for the subsequent cycles. For the purpose, the first regeneration cycle with release of CO_2_ was carried out as usual with the combination of NaI-H_3_PO_4_ and NaOH and subsequent chemical fixation of CO_2_ produced the spirooxazolidione. The subsequent cycles for the regeneration of spiroaziridine (CO_2_-release/defixation) and CO_2_-fixation were performed without further addition of NaI, only varying with the equivalent of H_3_PO_4_ and NaOH (Fig. 4). Thrillingly these were smoothly continued for five cycles. It showed almost quantitative yield of spirooxazolidinone **2a** in each cycle and finally the spirooxazolidinone **2a** was isolated with excellent overall yield (90%).

**Figure 5:**
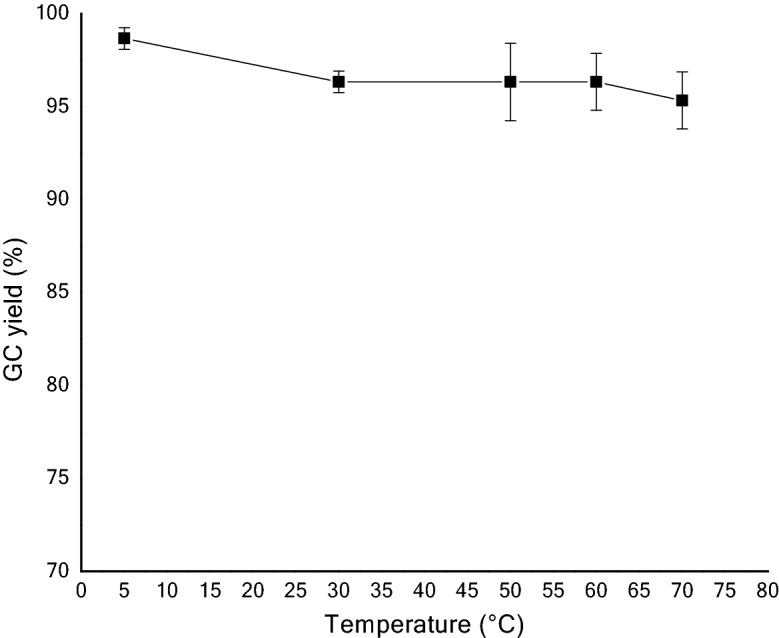
Temperature effect in chemical fixation of CO_2_ by spiroaziridine **1a**.

In some of the developed technologies, the sorbent (liquid or solid) loaded with the captured CO_2_ is transported to a different vessel, where it releases the CO_2_ (regeneration) either after being heated or after a pressure decrease or after any other change of conditions around the sorbent. The sorbent resulting after the regeneration step is sent back to capture more CO_2_ in a cycle. This makes additional cost of the process. It will be desirable to conduct both CO_2_ capture and the release in a single vessel, this is possible when both are near similar conditions. In our case, 70 °C was found to be optimum temperature for the CO_2_ defixation. So, we further studied the temperature effect on CO_2_ fixation. Interestingly, it showed a near horizontal line for the fixation at 5 °C, 30 °C, 50 °C, 60 °C and 70 °C, respectively, with > 95% yield in each case (Fig. 5).

**Figure 6:**
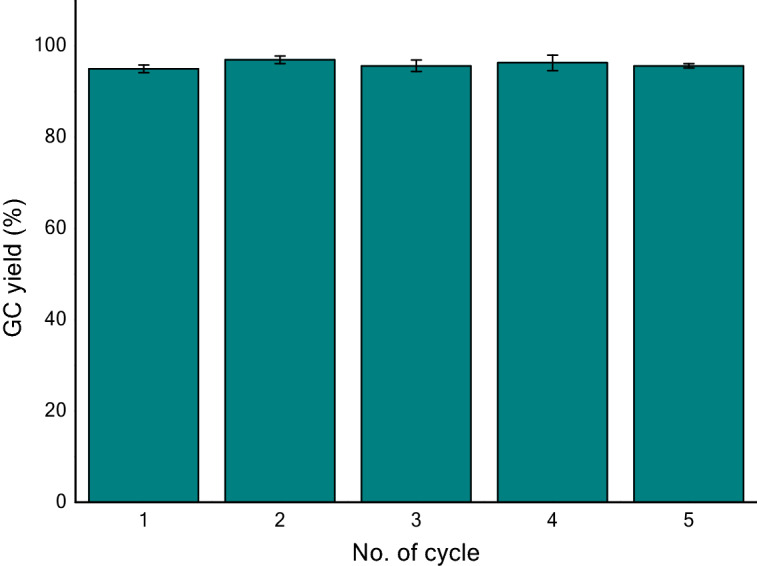
Chemical fixation-defixation cycles at 70 °C **(**The yield in each cycle referred to the GC yield of resynthesized spirooxazolidinone **2a**; Standard deviation: cycle 1 and 2 = 0.82; cycle 3 = 1.25, cycle 4 = 1.70, cycle 5 = 0.47).

Inspired by the above findings of temperature effect on CO_2_ fixation, we performed both CO_2_ defixation and fixation at 70 °C and continued for five cycles. With our great delight, it showed almost quantitative yield of spirooxazolidinone **2a** in each cycle and an excellent overall yield after five cycles. This chemical fixation-defixation (five) cycles at 70 °C are repeated for three times with a standard deviation of 0.47–1.70 (Fig. 6).

**Figure 7:**
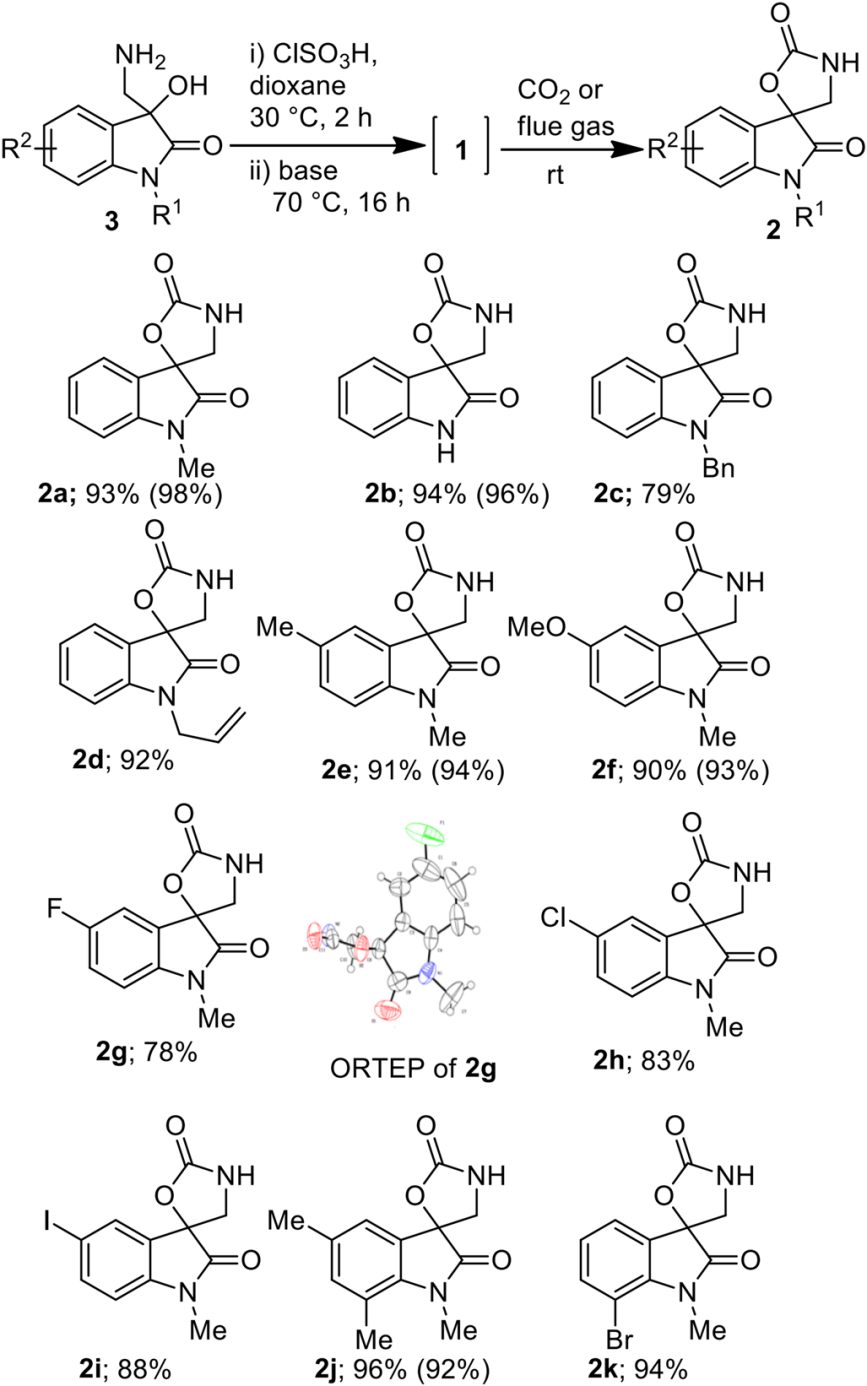
Generalization of catalyst-free CO_2_ fixation in synthesis of various spirooxazolidinoyl oxindoles **2**. The values in parenthesis refer to the GC yield using stimulated flue gas as a source of CO_2_.

The spirooxazolidonyl oxindoles are important bioactive compounds^21,22^. Thus further efforts are made to generalize the developed method for the synthesis of various spirooxazolidines by catalyst-free CO_2_ fixation of in situ generated spiroaziridines (Fig. 7). Irrespective of N-protection- and substitution of arene moiety of the oxindole unit, all underwent smooth auto-chemical fixation of CO_2_ providing the excellent isolated yields of the adducts **2**, albeit *N*-benzyl and *N*-allyl substrates took longer time in comparison with others for the CO_2_ fixation. Further, alike **1a**, the spiroaziridines derived from **3b**, **3e**, **3f** and **3j** also efficiently produced the corresponding CO_2_-adducts **2b**, **2e**, **2f** and **2j** with the flue gas in similar yields as with pure CO_2_. The regioselectivity of the fixation and the structure of the compound **2** was confirmed from the single crystal X-ray analysis of the compounds **2g** (Fig. 7; CCDC 1898609). All the CO_2_-adducts **2** are solid compounds with melting point > 100 °C and bench stable for a couple of months under ambient conditions. Thus the developed regenerable chemical fixation protocol can be utilized for CO_2_ capture, storage and release, if and when it needed.

## Conclusion

In summary, the first regenerable chemical fixation by spiroaziridine oxindole proved to be an excellent protocol for spontaneous and reversible CO_2_ fixation and defixation. We have demonstrated that the CCS [CO_2_ fixation and defixation cycle (regeneration)] can work well in one-pot (single vessel) for several cycles with excellent recovery using recyclable reagent under near ambient conditions. More importantly, the process regioselectively produced bioactive spirooxazolidinoyl oxindole in quantitative yields under extremely mild conditions (no extra reagent/catalyst, 1 atm., and rt). The CO_2_-adducts are stable compounds with high melting points, these can be stored for months under ambient conditions and can be reversed back to the sorbent as and when it requires. So, these findings in the ongoing research can open up a new avenue of the chemical fixation for the development of smart innovative technology towards the energy and cost effective practical CCS and would find abundant applications in CO_2_ fixation and -defixation chemistry towards chemical utilization of CO_2_ in industry.

## Methods

### Auto-chemical fixation of CO_2_ by in situ generated spiroaziridine 1a

Amino alcohol **3a** (500 mg, 2.60 mmol) was dissolved in dry dioxane (8 ml) and cooled to 0 ºC. Chlorosulfonic acid (174 µl, 2.6 mmol) was added drop wise and the reaction mixture was stirred for 2 h at room temperature (rt). 14 ml of 1 M aqueous NaOH solution was added dropwise to quench the acid at 0 ºC and stirred at 70 °C for 16 h. The complete conversion to spiroaziridine was detected by MS analysis. Next, a slow stream of CO_2_ was passed through the solution at rt for 30 min. After complete consumption of **1a** (monitored with TLC and also by MS analysis) the dioxane was removed under reduced pressure and the residue was extracted with EtOAc (3 × 10 ml), washed with brine solution and dried over anhydrous Na_2_SO_4_. Combined organic layer was concentrated and purified by silica gel flash chromatography using EtOAc/hexanes (1:1) to afford the desired CO_2_-adduct **2a** (528 mg, 93%).

*Note* In case of stimulated flue gas (12.5% CO_2_, 80% N_2_ and 7.5% O_2_) or 12.5% CO_2_ in N_2_, the stream of gas was passed through the solution for 18 h.

### Defixation of CO_2_ from spirooxazolidinone 2a and re-fixation of CO_2_

To a solution of spirooxazolidinone **2a** (150 mg, 0.69 mmol) in dry dioxane (6 ml), sodium iodide (414 mg, 2.76 mmol) and *o-*phosphoric acid (144 μl, 2.76 mmol) were added. The mixture was stirred at 70 °C and the consumption of **2a** was monitored by TLC and GC–MS. After 5 h, aqueous NaOH solution (0.7 M, 10 ml) was added and stirred for 30 min. The exclusive regeneration of spiroaziridine **1a** was confirmed by MS analysis. No spirooxazolidinone **2a** and iodoamine **4a** were detected in MS analysis at this stage. The crude solution containing spiroaziridineoxindole **1a** was further used for the chemical fixation of CO_2_. So, the slow stream of CO_2_ was passed through the solution for 30 min. The GC–MS analysis of the crude mixture with naphthalene as an internal standard showed quantitative formation of spirooxazolidinone **2a** (98%). Usual work and flash column chromatographic purification as discussed in general procedure gave the compound **2a** (143 mg, 95%).

### CO_2_-defixation and fixation cycles through in situ regeneration of spiroaziridine 1a and the isolation of spirooxazolidinone 2a

To a stirred solution of spirooxazolidinone **2a** (150 mg, 0.69 mmol) in dry dioxane (6 ml), sodium iodide (414 mg, 2.76 mmol) and *o-*phosphoric acid (144 μl, 2.76 mmol) were successively added at 70 °C and the reaction (consumption of **2a**) was monitored by TLC. After complete consumption of **2a** (5 h), it was brought to 0 °C and solid NaOH powder (390 mg, 9.75 mmol) was added to the reaction mixture. After attaining rt, it was stirred for additional 1 h. The stream of 100% CO_2_ was passed through to the suspended mixture for 1 h at rt. The solid mass was filtered off and washed with dioxane (2 × 5 ml). The combined organic solvent was evaporated to dryness under reduced pressure. The crude compound was dissolved in dioxane (6 ml) and 150 μl of the solution was taken out for the GC–MS analysis with naphthalene (5 mg) as an internal standard. The analysis showed 97% yield of the spirooxazolidinone **2a**. So the calculated amount of resynthesized **2a** was found to be 148.5 mg and 150 μl of the solution contained 3.7 mg of **2a**. The resynthesized compound **2a** (148.5 − 3.7 = 144.8 mg) was used for the second cycle for the regeneration of spiroaziridine and the fixation of CO_2_ using the same procedure as mentioned above i.e. the use of NaI-H_3_PO_4_, solid NaOH and the stream of CO_2_. The GC-yield of the second cycle was observed to be 96%. Similarly, another three cycles were carried and the GC-yields were found to be 99%, 97% and 95%, respectively.

### One-pot CO_2_-defixation and fixation cycles without isolation of re-synthesized spirooxazolidinone

The one-pot CO_2_-defixation and fixation cycles were carried out following the similar procedure as above without separating out the solid by-products and isolation of re-synthesized spirooxazolidinone in the intermediate cycles.

To a stirred solution of spirooxazolidinone **2a** (150 mg, 0.69 mmol) in dry dioxane (6 ml), sodium iodide (414 mg, 2.76 mmol) and *o-*phosphoric acid (144 μl, 2.76 mmol) were successively added at 70 °C and the reaction (consumption of **2a**) was monitored by TLC. After complete consumption of **2a** (5 h), it was brought to rt and solid NaOH powder (390 mg, 9.75 mmol) was added to the reaction mixture at 0 °C. After attaining to rt, it was stirred for additional 1 h. The stream of CO_2_ was passed through to the suspended mixture for 1 h at rt. The complete consumption of in situ regenerated spiroaziridine and the formation of spirooxazolidinone **2a** were monitored by TLC and MS analysis. Without separating out the solid mass and the isolation of spirooxazolidinone, another consecutive four cycles were repeated by adding the same amount of sodium iodide and *o-*phosphoric acid followed by solid NaOH and the stream of CO_2_ for each cycle in the same pot. The consumption of the intermediate substrate and regeneration of the product were monitored during each cycles by TLC and MS analysis. At the end of 5^th^ cycles, the solid mass was filtered off and washed with dioxane (3 × 10 ml). The combined organic solvent was evaporated to dryness under reduced pressure. The crude compound was dissolved in dioxane (6 ml) and the GC–MS analysis with naphthalene as an internal standard showed 95% overall yield of the spirooxazolidinone **2a** for the five cycles. The silica gel flash column chromatographic purification of the crude with hexanes-EtOAc (1:1) gave the spirooxazolidinone **2a** (134.9 mg, 90% overall yield) as a white solid.

### Recycling of spiroaziridine and NaI for the fixation- and defixation of CO_2_

To a stirred solution of spirooxazolidinone **2a** (150 mg, 0.69 mmol) in dry dioxane (6 ml), sodium iodide (414 mg, 2.76 mmol) and *o-*phosphoric acid (144 μl, 2.76 mmol) were successively added at rt and the reaction (consumption of **2a**) was monitored by TLC. After complete consumption of **2a** (5 h), solid NaOH powder (342 mg, 8.6 mmol) was added to the reaction mixture at 0 °C. After attaining to rt, it was stirred for additional 1 h. The stream of CO_2_ was passed through to the suspended mixture for 1 h at rt. The complete consumption of in situ regenerated spiroaziridine and the formation of spirooxazolidinone **2a** (97% GC yield) were monitored by TLC and MS analysis. For the next cycle, the reaction mixture was acidified with *o-*phosphoric acid (292 μl, 5.6 mmol) and stirred at 70 °C without further addition of sodium iodide. After complete consumption of the spirooxazolidinone (monitored with TLC), solid NaOH powder was added (694 mg, 17.36 mmol) and the stream of CO_2_ was passed through for 1 h to reproduce the spirooxazolidinone **2a** (98%, GC yield). This process was repeated for five consecutive cycles. GC–MS analysis showed almost quantitative yield of spirooxazolidinone in each stage and finally the spirooxazolidinone **2a** (135.0 mg, 90%) was isolated after fifth cycle by flash chromatography using hexanes-EtOAc (1:1).

*Note* (a) GC yield is determined by using naphthalene as internal standard; (b) the release of CO_2_ from spiroxazolidinone and its subsequent regeneration using CO_2_ fixation is considered as one complete cycle. (c) At constant temperature (70 °C) five consecutive cycles of CO_2_ fixation and defixation was accomplished using above method (Supplementary material; General procedure 2). GC yield in resynthesis of spirooxazolidione **2a** was monitored at each stage (Fig. 3).

## Supplementary information

Supplementary Information.
