# Supplementary material for: Efficient chemical fixation and defixation cycle of carbon dioxide under ambient conditions
Source: Sci Rep. 2020 Sep 25;10:15825. doi: 10.1038/s41598-020-71761-w (PMC7519152; doi:10.1038/s41598-020-71761-w)
Supplement: Supplementary file 1 — Supplementary Information. [file 41598_2020_71761_MOESM1_ESM.pdf]

Supplementary Information

Efficient chemical fixation and defixation cycle of carbon dioxide under ambient conditions

Saumen Hajra* and Anurag Biswas

Center of Biomedical Research, Sanjay Gandhi Post-Graduate Institute of Medical Sciences Campus, Raebareli Road, Lucknow 226014, India

General Information	**S2**
Synthesis of NH-free spiroaziridine **1a**	**S2**
General procedure for auto-chemical fixation of CO_2	**S3**
General procedure for the defixation of CO_2 from spirooxazolidinoe **2a** and re-fixation of CO_2	**S4**
General Procedure 1: CO_2-Defixation and fixation cycles via isolation of **2a**	**S4**
General Procedure 2: One-pot recycling of spiroaziridine and spirooxazolidione for the fixation- and defixation of CO_2	**S5**
Temperature effect in chemical fixation of CO_2	**S6**
One-pot experiment for recycling of spirooxazolidione for the defixation- and fixation of CO_2 via GC-MS analysis	**S6-S30**
Spectral data of spirooxazolidinones **2**	**S31-S34**
Synthesis of compounds **5a** and **6a** and spectral data	**S35-S37**
References	**S37**
X-ray crystal structure details of **2g**	**S38-S39**
NMR spectra	**S40-S53**

General information

All chemicals were used as received. The solvents were dried by standard procedures, distilled and stored under nitrogen. Flash column chromatography was performed in all cases using the indicated solvent system on silica gel (230-400 mesh) purchased. Analytical thin layer chromatography was performed using 60 F^{254} percolated silica gel plate (0.2 mm thickness) and compounds were visualized by irradiation of UV light. The ^{1}H NMR and ^{13}C NMR spectra were measured with 400 MHz using $CDCl_3$ or DMSO-d_6 or C_6D_6. Splitting patterns were reported as s = singlet, d = doublet, dd = doublet of doublet, t = triplet, q = quartet, m = multiplet, br = broad. Electro spray ionization (ESI) mass spectrometry (MS) experiments were performed on Agilent Technologies 6530 Accurate-Mass Q-TOF LC/MS from Centre of Biomedical Research (CBMR). GC-MS (Agilent Technology) was obtained from CBMR and for the analysis, RAM temperature was used 50 °C for each sample.

Amino alcohol **3** was synthesized in 1 g scale from spiroepoxide by following the literature procedure.[1]

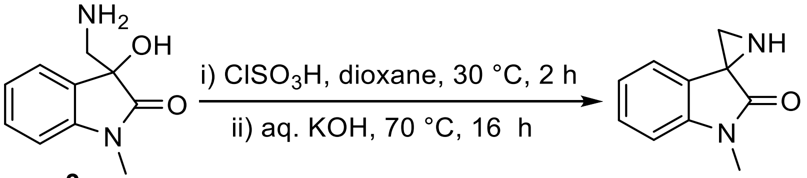

3a **1a**

Synthesis of 1'-methylspiro[aziridine-2,3'-indolin]-2'-one 1a: To a stirred solution of amino alcohol **3a** (100 mg, 0.52 mmol) in dry dioxane (4 ml), chlorosulphonic acid (35 μL, 0.52 mmol) was added at 0 °C. Reaction mixture was warmed to room temperature and stirred for 2 h. 3 ml of 1 M aqueous KOH solution was added drop wise to quench the acid at 0 ºC and stirred at 70 °C for 16 h. The complete conversion to spiroaziridine was detected by MS analysis. The reaction mixture was further diluted with water (5 ml) and extracted with EtOAc (3x10 ml), washed with brine solution and dried over anhydrous Na_2SO_4. Combined organic layer was concentrated under reduced pressure at rt and afforded a gummy liquid. However, MS analysis of the gummy liquid did not show the desired mass of **1a**. It seems the spiroaziridine **1a** undergoes decomposition/polymerization in neat condition and also on silica gel as an attempt to purify by silica gel was also not successful.

Instead of aqueous of KOH, treatment with solid KOH also showed the complete conversion to spiroaziridine **1a**. It is worth to note that both aqueous and non-aqueous solution of spiroaziridine **1a** in dioxane is stable for a few weeks under ambient condition in N_2 or Ar

atmosphere. For the detail characterization of spiroaziridine **1a**, it was synthesized in deuterated solvent like C_6D_6 as follow.

To a stirred solution of amino alcohol **3a** (100 mg, 0.52 mmol) in dry dioxane (4 ml), chlorosulphonic acid (35 μL, 0.52 mmol) was added at 0 °C. Reaction mixture was warmed to room temperature and stirred for 2 h prior to treatment of base. The reaction mixture was diluted with ether (20 ml) and a white precipitate was obtained. It was filtered and washed thoroughly with ether (3x7 ml) and dried. The salt was added to a biphasic mixture of C_6D_6 (4 ml) and 0.3 M aqueous NaOH (9 ml). The suspended mixture was stirred at 70 °C and monitored by TLC and MS analysis. After 16 h, the C_6D_6 solution was separated out and dried over Na_2SO_4. The formation of spiroaziridine **1a** was analyzed by NMR and MS. As mentioned earlier, the isolation of neat **1a** was not successful, as it is prone to decomposition/polymerization. However, the compound **1a** in benzene or dioxane was found to be stable under inert condition at room temperature.

^{1}H NMR (400 MHz, C_6D_6) δ 6.94 (ddd, *J* = 7.8, 7.2, 1.8 Hz, 1H), 6.73 – 6.66 (m, 2H), 6.13 (d, *J* = 7.8 Hz, 1H), 3.95 (d, *J* = 0.8 Hz, 1H), 3.27 (d, *J* = 6.9 Hz, 1H), 2.72 (d, *J* = 6.9 Hz, 1H), 2.52 (s, 3H).^{13}C NMR (100 MHz, C_6D_6) δ 170.7, 145.3, 129.7, 123.0, 121.9, 121.7, 108.2, 55.9, 53.3, 25.5; HRMS (ESI) calcd for $C_{10}H_{11}N_2O$ [M+H]+ = 175.0871, found 175.0867.

General procedure for auto-chemical fixation of CO_2 by in situ generated spiroaziridine 1a

Amino alcohol **3a** (500 mg, 2.60 mmol) was dissolved in dry dioxane (8 ml) and cooled to 0 ºC. Chlorosulfonic acid (174 μL, 2.60 mmol) was added drop wise and the reaction mixture was stirred for 2 h at room temperature (rt). 13 ml of 1 M aqueous NaOH solution was added dropwise to quench the acid at 0 ºC and stirred at 70 °C for 16 h. The complete conversion to spiroaziridine was detected by MS analysis. Next, a slow stream of CO_2 was passed through the solution at rt for 30 min. After complete consumption of **1a** (monitored with TLC and also by MS analysis) the dioxane was removed under reduced pressure and the residue was extracted with EtOAc (3×10 ml), washed with brine solution and dried over anhydrous Na_2SO_4. Combined organic layer was concentrated and purified by silica gel flash chromatography using EtOAc/hexanes (1:1) to afford the desired CO_2-adduct **2a** (528 mg, 93%).

Note: In case of stimulated flue gas (12.5% CO_2, 80% N_2 and 7.5% O_2) or 12.5% CO_2 in N_2, the stream of gas was passed through the solution for 18 h.

General procedure for the defixation of CO_2 from spirooxazolidinoe 2a and re-fixation of CO_2

To a solution of spirooxazolidinone **2a** (150 mg, 0.69 mmol) in dry dioxane (6 ml), sodium iodide (414 mg, 2.76 mmol) and *o*-phosphoric acid (144 μL, 2.76 mmol) were added. The mixture was stirred at 70 °C and the consumption of **2a** was monitored by TLC and GC-MS. After 5 h, aqueous NaOH solution (0.7 M, 12 ml) was added and stirred for 30 min. The exclusive regeneration of spiroaziridine **1a** was confirmed by MS analysis. No spirooxazolidinone **2a** and iodoamine **4a** were detected in MS analysis at this stage. The crude solution containing spiroaziridineoxindole **1a** was further used for the chemical fixation of CO_2. So, the slow stream of CO_2 was passed through the solution for 30 min. The GC-MS analysis of the crude mixture with naphthalene as an internal standard showed quantitative formation of spirooxazolidinone **2a** (98%). Usual work and flash column chromatographic purification as discussed in general procedure gave the compound **2a** (143 mg, 95%).

General Procedure 1: CO_2-Defixation and fixation cycles through in situ regeneration of spiroaziridine 1a and the isolation of spirooxazolidinone 2a

To a stirred solution of spirooxazolidinone **2a** (150 mg, 0.69 mmol) in dry dioxane (6ml), sodium iodide (413 mg, 2.76 mmol) and *o*-phosphoric acid (144 μL, 2.76 mmol) were successively added at 70 ºC and the reaction (consumption of **2a**) was monitored by TLC. After complete consumption of **2a** (5 h), it was brought to 0 °C and solid NaOH powder (390 mg, 9.75mmol) was added to the reaction mixture. After attaining rt, it was stirred for additional 1 h. The stream of 100% CO_2 was passed through to the suspended mixture for 1 h at rt. The solid mass was filtered off and washed with dioxane (2x5 ml). The combined organic solvent was evaporated to dryness under reduced pressure. The crude compound was dissolved in dioxane (6 ml) and 150 μL of the solution was taken out for the GC-MS analysis with naphthalene (5 mg) as an internal standard. The analysis showed 97% yield of the spirooxazolidinone **2a**. So, the calculated amount of resynthesized **2a** was found to be 148.5 mg and 150 μL of the solution contained 3.7 mg of **2a**. The resynthesized compound **2a** (148.5 – 3.7 = 144.8 mg) was used for the second cycle for the regeneration of spiroaziridine

and the fixation of CO_2 using the same procedure as mentioned above *i.e.* the use of NaI-H_3PO_4, solid NaOH and the stream of CO_2. The GC-yield of the second cycle was observed to be 96%. Similarly, another three cycles were carried and the GC-yields were found to be 99%, 97% and 95%, respectively.

General Procedure 2: One-pot recycling of spiroaziridine and spirooxazolidione for the fixation- and defixation of CO_2

To a stirred solution of spirooxazolidinone **2a** (150 mg, 0.69 mmol) in dry dioxane (6 ml), sodium iodide (414 mg, 2.76 mmol) and *o*-phosphoric acid (144 μL, 2.76 mmol) were successively added at rt and the reaction (consumption of **2a**) was monitored by TLC. After complete consumption of **2a** (5 h), solid NaOH powder (342 mg, 8.6 mmol) was added to the reaction mixture at 0 °C. After attaining to rt, it was stirred for additional 1 h. The stream of CO_2 was passed through to the suspended mixture for 1 h at rt. The complete consumption of in situ regenerated spiroaziridine and the formation of spirooxazolidinone **2a** (97% GC yield) were monitored by TLC and MS analysis. For the next cycle, the reaction mixture was acidified with *o*-phosphoric acid (292 μL, 5.6 mmol) and stirred at 70 °C without further addition of sodium iodide. After complete consumption of the spirooxazolidinone (monitored with TLC), solid NaOH powder was added (694 mg, 17.36 mmol) and the stream of CO_2 was passed through for 1 h to reproduce the spirooxazolidinone **2a** (98%, GC yield). This process was repeated for five consecutive cycles. GC-MS analysis showed almost quantitative yield of spirooxazolidinone in each stage and finally the spirooxazolidinone **2a** (135.0 mg, 90%) was isolated after fifth cycle by flash chromatography using hexanes-EtOAc (1:1).

Note: a) GC yield is determined by using naphthalene as internal standard; **b)** The release of CO_2 from spiroxazolidinone and its subsequent regeneration using CO_2 fixation is considered as one complete cycle.

Chemical fixation of CO_2 by NH-free spiroaziridine oxindole 1a at different temperature

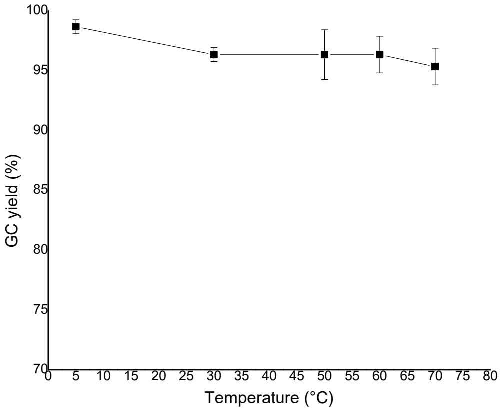

Temperature (°C)	Mean GC yield (%)	Standard Deviation
5	98.66	0.57
30	96.33	0.57
50	96.33	2.08
60	96.33	1.52
70	95.33	1.52

Temperature effect in chemical fixation of CO_2 by spiroaziridine

One-pot recycling experiment of spirooxazolidione for the fixation- and defixation of CO_2:

Experimental temperature: 30 °C

Trial I:

<u>Cycle I-1:</u>

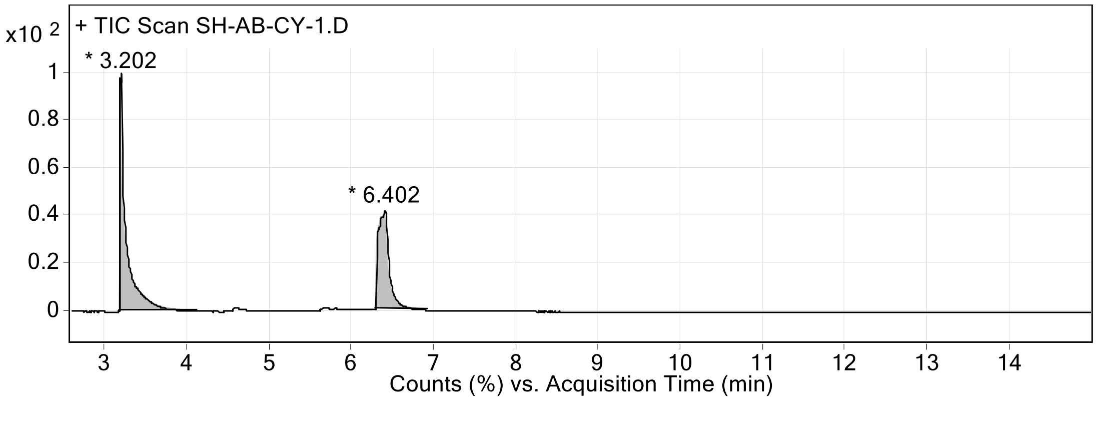

Integration Peak List				
**Peak**	**RT**	**Height**	**Area**	**Area Sum Percent**
1. Naphthalene	3.202	1930385.97	9792614.73	56.54
2. Spiro-oxazolidinone	6.402	796192.76	7528592.26	43.46

Yield calculation:

Initial amount of **2a** for defixtion**:** 150 mg, 0.69 mmol

Volume of crude in dioxane: 5 ml

Internal Std. (Naphthalene): 3.3 mg, 0.026 mmol

Volume of **2a** dioxane solution for GC-Ms analysis: 150 µL (contains 4.36 mg of **2a**)

Quantitative estimation of **2a** in crude solution: 145.5 mg, 0.67 mmol

Yield: 97%

Cycle I-2:

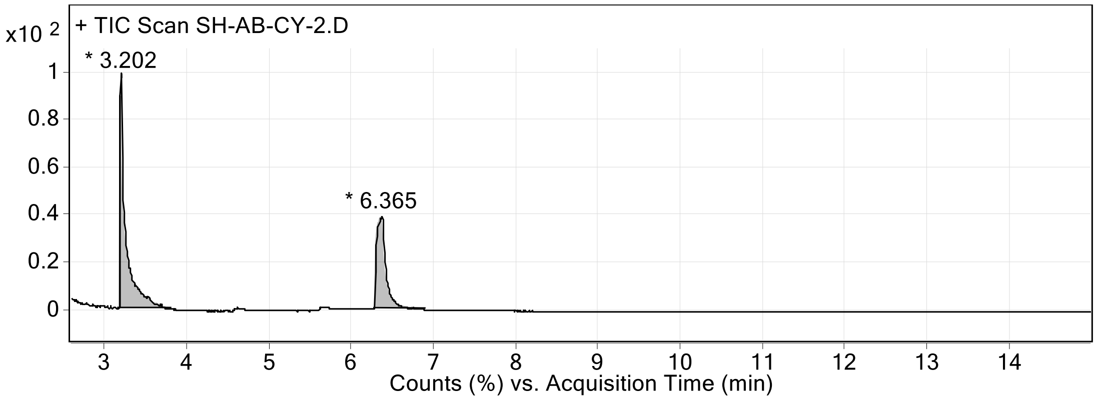

Integration Peak List				
**Peak**	**Area**	**Area Sum Percent**	**RT**	**Height**
1. Naphthalene	7335606.22	60.59	3.202	1509927.2
2. Spiro-oxazolidinone	4770631.52	39.41	6.365	589464.11

Yield calculation:

Initial amount of **2a** for defixtion**:** 141.14 mg (= 145.5 mg - 4.36 mg), 0.647mmol

Volume of crude in dioxane: 5 ml

Internal Std. (Naphthalene): 3.67 mg, 0.0287 mmol

Volume of **2a** dioxane solution for GC-MS analysis: 150 µL (contains 4.06 mg of **2a**)

Quantitative estimation of **2a** in crude solution: 135.3 mg, 0.62 mmol

Yield: 96%

Cycle I-3:

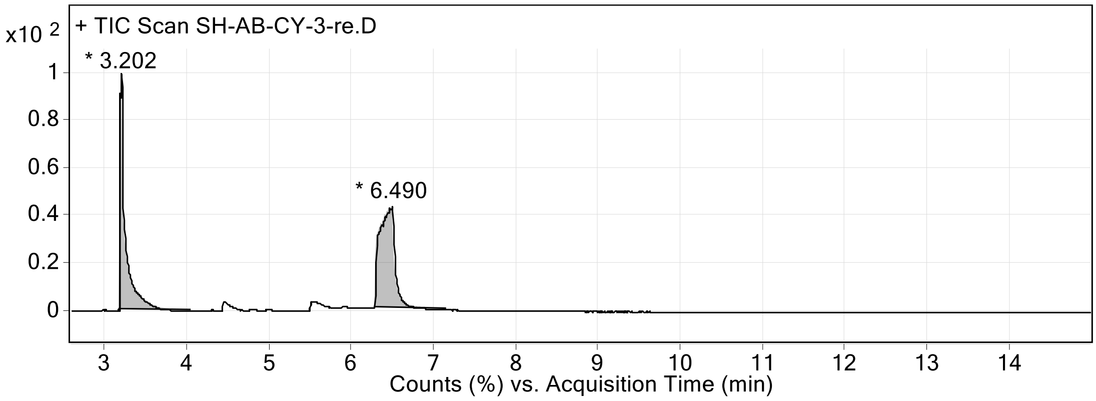

Integration Peak List				
**Peak**	**Area**	**Area Sum Percent**	**RT**	**Height**
1. Naphthalene	9492767	44.09	3.202	2108254.12
2. Spiro-oxazolidinone	12036141.22	55.91	6.49	905129.02

Yield calculation:

Initial amount of **2a** for defixtion**:** 131.27mg (=135.33 mg - 4.06 mg), 0.602 mmol

Volume of crude in dioxane: 5 ml

Internal Std. (Naphthalene): 3.07 mg, 0.0141mmol

Volume of **2a** dioxane solution for GC-Ms analysis: 150 µL (contains 3.90 mg of **2a**)

Quantitative estimation of **2a** in crude solution: 129.9 mg, 0.596 mmol

Yield: 99%

Cycle I-4:

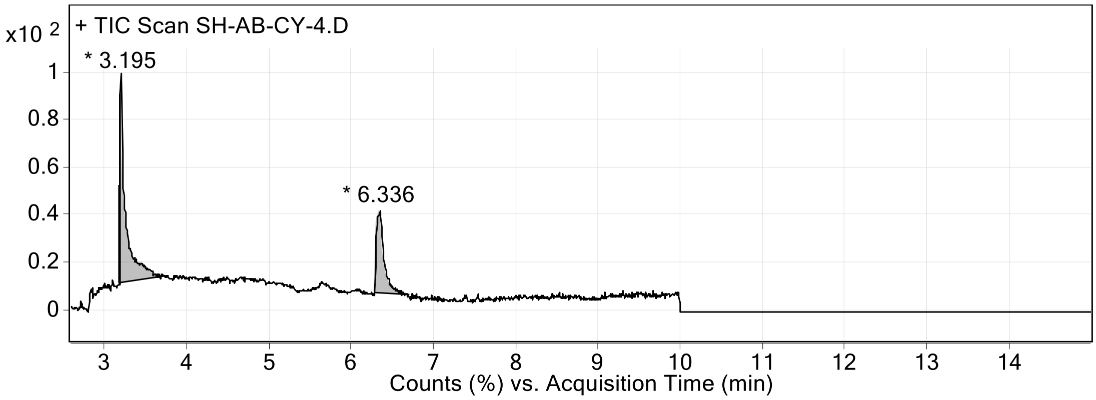

Integration Peak List				
**Peak**	**Area**	**Area Sum Percent**	**RT**	**Height**
1. Naphthalene	6908939.68	64.44	3.195	1444178.83
2. Spiro-oxazolidinone	3812747.82	35.56	6.336	571040.18

Yield calculation:

Initial amount of **2a** for defixtion**:** 126.06 mg (=129.96 mg - 3.90 mg), 0.602 mmol

Volume of crude in dioxane: 5 ml

Internal Std. (Naphthalene): 3.90 mg, 0.0304 mmol

Volume of **2a** dioxane solution for GC-Ms analysis: 150 µL (contains 3.66 mg of **2a**)

Quantitative estimation of **2a** in crude solution: 121.9 mg, 0.559mmol

Yield: 97%

Cycle I-5:

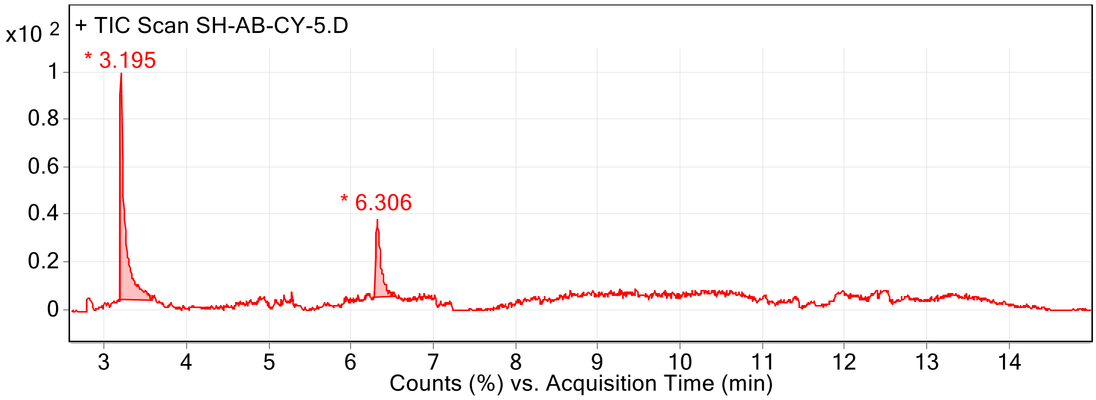

**Integration Peak List**				
**Peak**	**Area**	**Area Sum Percent**	**RT**	**Height**
1. Naphthalene	5980273.72	72.94	3.195	1379460.58
2. Spiro-oxazolidinone	2218933.78	27.06	6.306	481589.99

Yield calculation:

Initial amount of **2a** for defixtion**:** 118.61 mg (=122.28 mg - 3.67 mg), 0.602 mmol

Volume of crude in dioxane: 5 ml

Internal Std. (Naphthalene): 5.350 mg, 0.0418 mmol

Volume of **2a** dioxane solution for GC-Ms analysis: 150 µL (contains 3.38 mg of **2a**)

Quantitative estimation of **2a** in crude solution: 112.69 mg, 0.517 mmol

Yield: 95%

Trial II:

Cycle II-1:

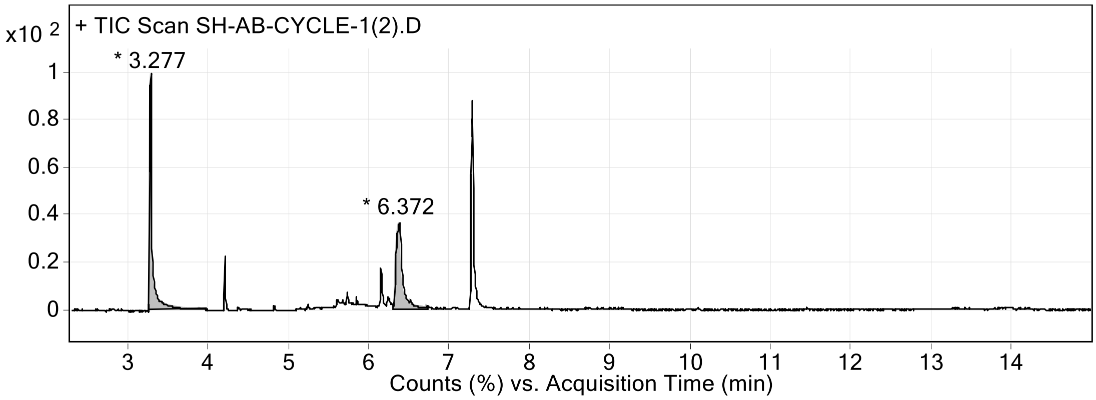

**Integration Peak List**				
**Peak**	**Area**	**Area Sum Percent**	**RT**	**Height**
1. Naphthalene	68774919.99	54.48	3.277	25079419.37
2. Spiro-oxazolidinone	57469178.35	45.52	6.372	9254016.88

Yield calculation:

Initial amount of **2a** for defixtion**:** 150 mg, 0.688 mmol

Volume of crude in dioxane: 5 ml

Internal Std. (Naphthalene): 3.1 mg, 0.0242mmol

Volume of **2a** dioxane solution for GC-Ms analysis: 150 µL (contains 4.41 mg of **2a**)

Quantitative estimation of **2a** in crude solution: 146.9 mg, 0.674 mmol

Yield: 98%

Cycle II-2:

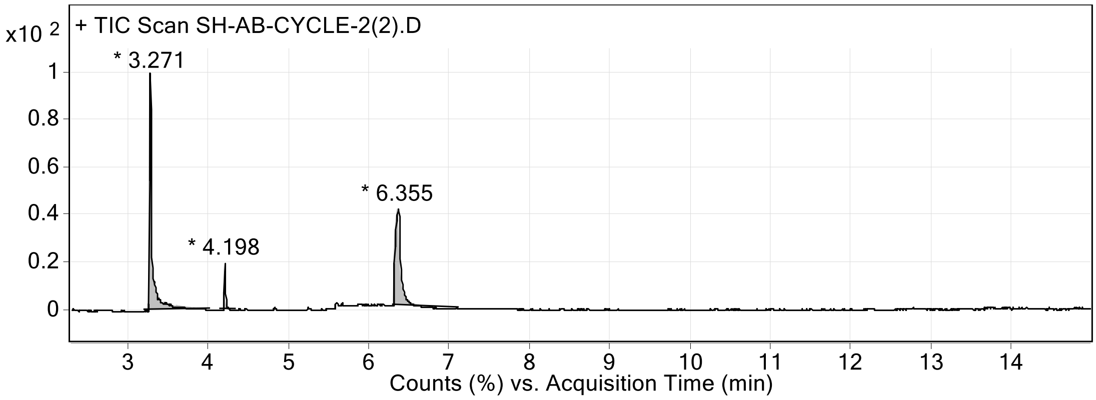

**Integration**

Peak List				
**Peak**	**Area**	**Area Sum Percent**	**RT**	**Height**
1. Naphthalene	54273886.39	51.63	3.271	28609479.64
2. Spiro-oxazolidinone	50851918.25	48.37	6.355	11353640.2

Yield calculation:

Initial amount of **2a** for defixtion**:** 142.59 mg (=147 mg - 4.41 mg), 0.654 mmol

Volume of crude in dioxane: 5 ml

Internal Std. (Naphthalene): 2.55 mg, 0.0199 mmol

Volume of **2a** dioxane solution for GC-Ms analysis: 150 µL (contains 4.06 mg of **2a**)

Quantitative estimation of **2a** in crude solution: 135.5 mg, 0.621 mmol

Yield: 95%

Cycle II-3:

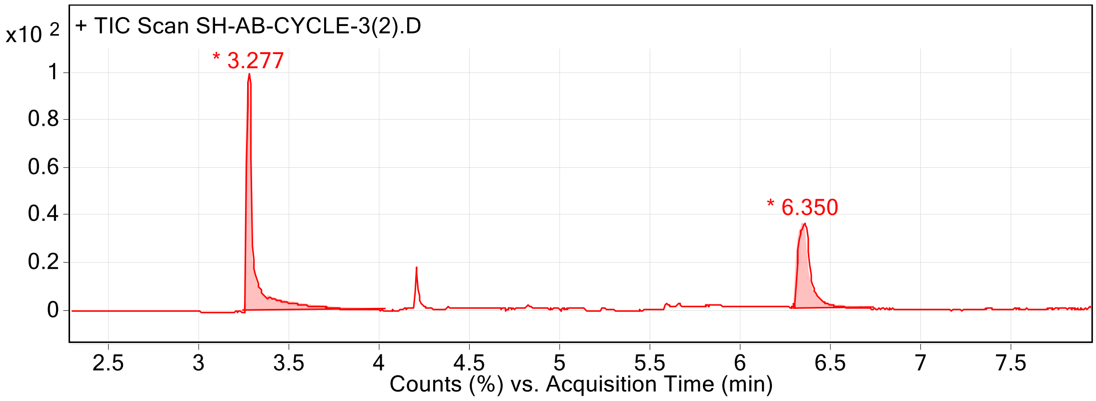

Integration Peak List				
**Peak**	**Area**	**Area Sum Percent**	**RT**	**Height**
1. Naphthalene	79655099.18	62.55	3.277	25677621.52
2. Spiro-oxazolidinone	47699755.46	37.45	6.35	9239351.22

Yield calculation: Initial amount of **2a** for defixtion**:** 131.4 mg (=135.46 mg - 4.06 mg), 0.603 mmol

Volume of crude in dioxane: 5 ml

Internal Std. (Naphthalene): 3.75 mg, 0.0293 mmol

Volume of **2a** dioxane solution for GC-Ms analysis: 150 µL (contains 3.82 mg of **2a**)

Quantitative estimation of **2a** in crude solution: 127.3 mg, 0.584 mmol

Yield: 97%

Cycle II-4:

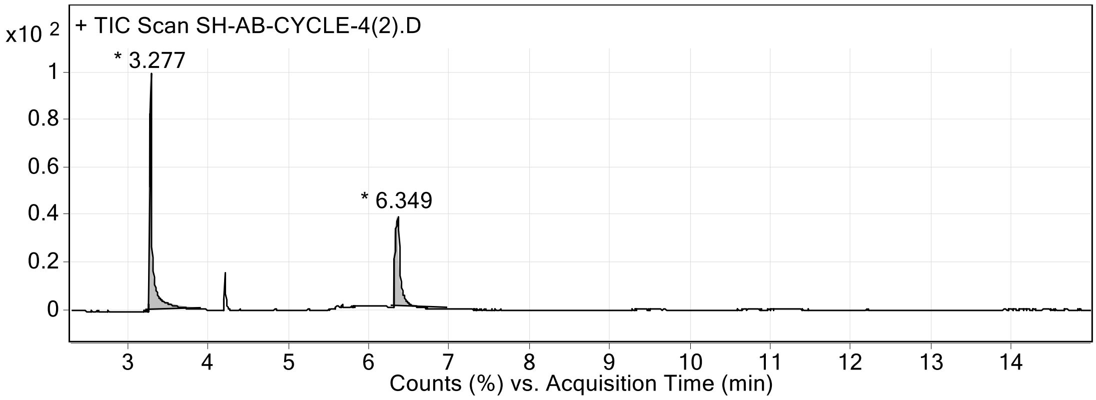

**Integration Peak List**				
**Peak**	**Area**	**Area Sum Percent**	**RT**	**Height**
1. Naphthalene	74827144.12	60.02	3.277	28565448.54
2. Spiro-oxazolidinone	49840209.86	39.98	6.349	10822784.83

Yield calculation: Initial amount of **2a** for defixtion**:** 123.68 mg (=127.5 mg - 3.82 mg), 0.567 mmol

Volume of crude in dioxane: 5 ml

Internal Std. (Naphthalene): 3.11 mg, 0.0243mmol

Volume of **2a** dioxane solution for GC-Ms analysis: 150 µL (contains 3.53 mg of **2a**)

Quantitative estimation of **2a** in crude solution: 117.6 mg, 0.54 mmol

Yield: 95%

Cycle II-5:

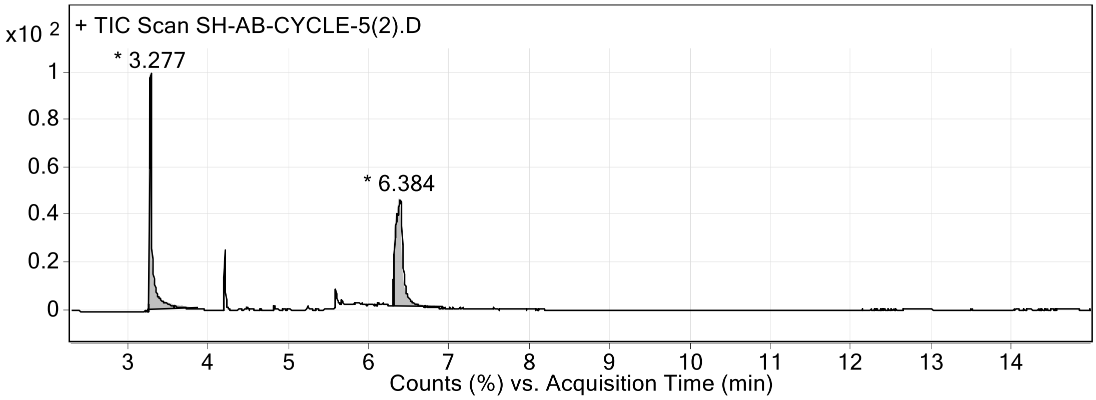

**Integration Peak List**				
**Peak**	**Area**	**Area Sum Percent**	**RT**	**Height**
1. Naphthalene	78769255.78	47.42	3.277	28952094.38
2. Spiro-oxazolidinone	87338208.09	52.58	6.384	13106843.91

Yield calculation: Initial amount of **2a** for defixtion**:** 113.966 mg (= 117.496 mg - 3.53 mg), 0.523 mmol

Volume of crude in dioxane: 5 ml

Internal Std. (Naphthalene): 1.75 mg, 0.0137mmol

Volume of **2a** dioxane solution for GC-Ms analysis: 150 µL (contains 3.31 mg of **2a**)

Quantitative estimation of **2a** in crude solution: 110.4 mg, 0.506 mmol

Yield: 97%

Trial III:

Cycle III-1:

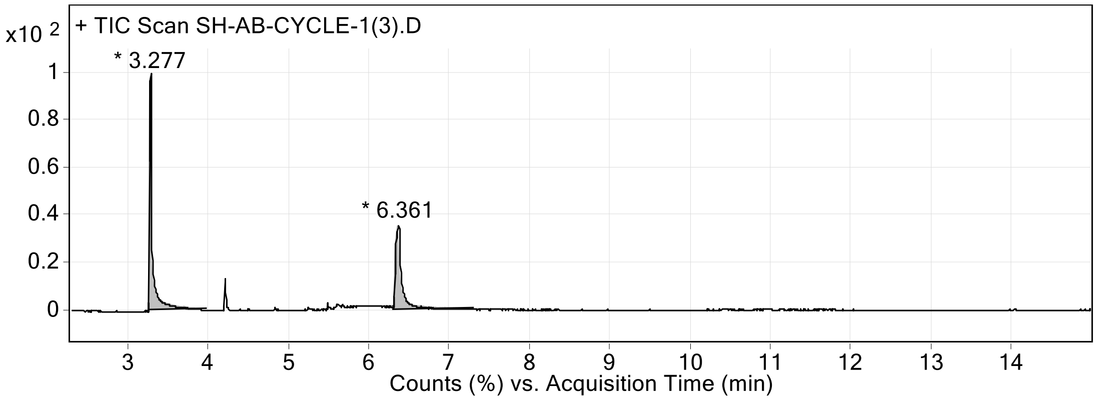

**Integration Peak List**				
**Peak**	**Area**	**Area Sum Percent**	**RT**	**Height**
1. Naphthalene	76807638.63	56.96	3.277	27266453.92
2. Spiro-oxazolidinone	58034973.6	43.04	6.361	9647509.93

Yield calculation: Initial amount of **2a** for defixtion**:** 150 mg, 0.688 mmol

Volume of crude in dioxane: 5 ml

Internal Std. (Naphthalene): 3.39 mg, 0.0265 mmol

Volume of **2a** dioxane solution for GC-Ms analysis: 150 µL (contains 4.37 mg of **2a**)

Quantitative estimation of **2a** in crude solution: 145.5 mg, 0.667 mmol

Yield: 97%

Cycle III-2:

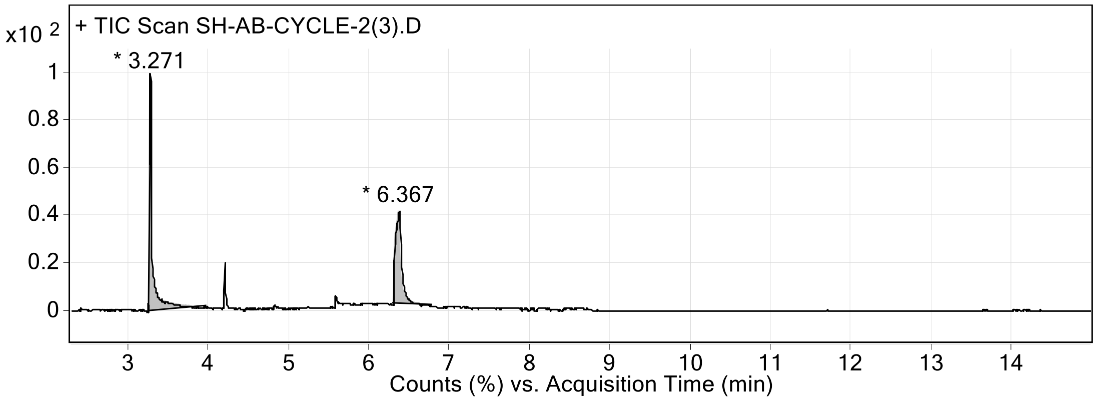

**Integration**

Peak List				
**Peak**	**Area**	**Area Sum Percent**	**RT**	**Height**
1. Naphthalene	69315918.05	55.58	3.271	25253754.87
2. Spiro-oxazolidinone	55404883.24	44.42	6.367	9917017.96

Yield calculation: Initial amount of **2a** for defixtion**:** 141.04 mg (=145.4 mg - 3.53 mg), 0.647 mmol

Volume of crude in dioxane: 5 ml

Internal Std. (Naphthalene): 2.98 mg, 0.0233mmol

Volume of **2a** dioxane solution for GC-Ms analysis: 150 µL (contains 4.06 mg of **2a**)

Quantitative estimation of **2a** in crude solution: 135.3 mg, 0.621 mmol

Yield: 96%

__**Cycle III-3:**__

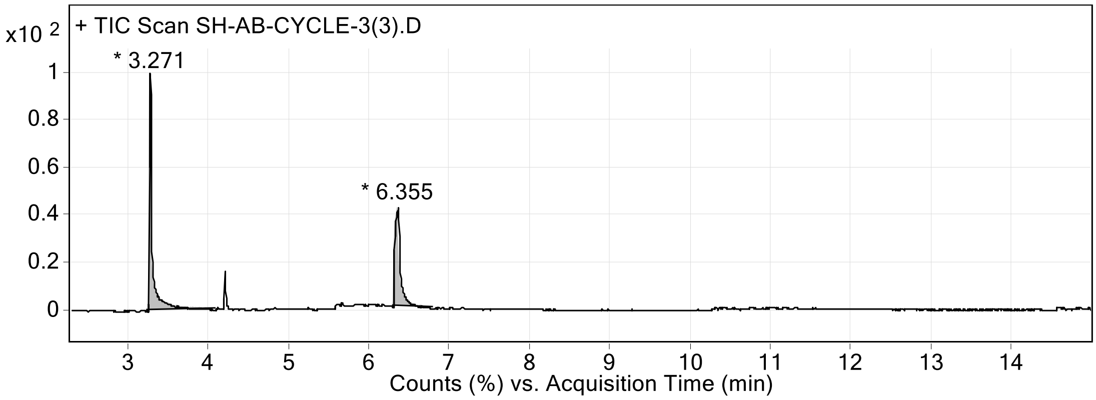

Integration Peak List				
**Peak**	**Area**	**Area Sum Percent**	**RT**	**Height**
1. Naphthalene	61579493.26	57.05	3.271	23123228.65
2. Spiro-oxazolidinone	46360049.82	42.95	6.355	9594942.17

Yield calculation: Initial amount of **2a** for defixtion**:** 131.34 mg (=135.4 mg - 4.06 mg), 0.602 mmol

Volume of crude in dioxane: 5 ml

Internal Std. (Naphthalene): 3.011 mg, 0.0235mmol

Volume of **2a** dioxane solution for GC-Ms analysis: 150 µL (contains 3.86 mg of **2a**)

Quantitative estimation of **2a** in crude solution: 128.6 mg, 0.590 mmol

Yield: 98%

Cycle III-4:

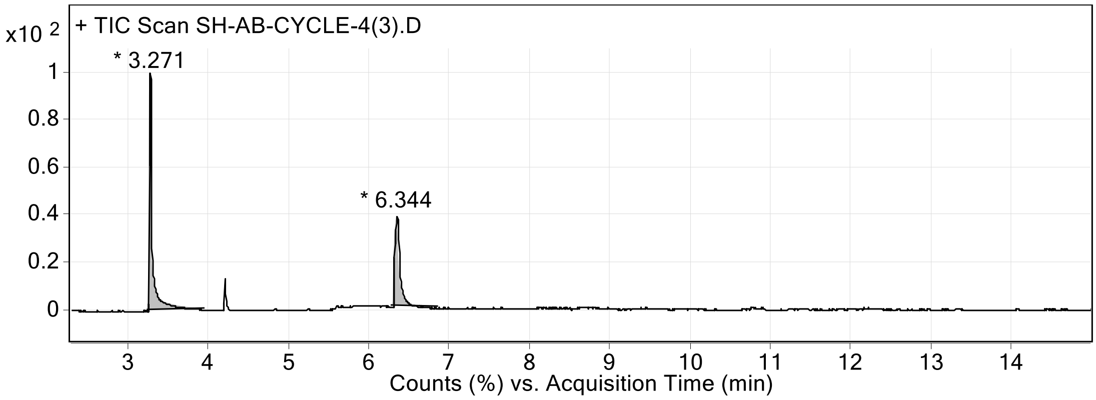

**Integration Peak List**				
**Peak**	**Area**	**Area Sum Percent**	**RT**	**Height**
1. Naphthalene	70768526.49	61.39	3.271	26353240.34
2. Spiro-oxazolidinon e	44511321.77	38.61	6.344	10012780.88

Yield calculation:

Initial amount of **2a** for defixtion**:** 124.70 mg (=128.56 mg - 3.86 mg), 0.572 mmol

Volume of crude in dioxane: 5 ml

Internal Std. (Naphthalene): 3.353 mg, 0.0262 mmol

Volume of **2a** dioxane solution for GC-Ms analysis: 150 µL (contains 3.59 mg of **2a**)

Quantitative estimation of **2a** in crude solution: 119.7 mg, 0.549mmol

Yield: 96%

Cycle III-5:

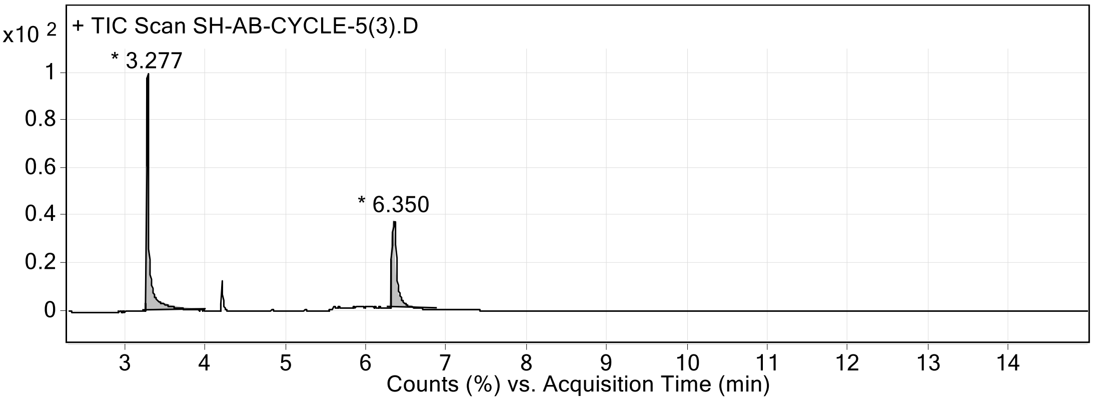

**Integration Peak List**				
**Peak**	**Area**	**Area Sum Percent**	**RT**	**Height**
1. Naphthalene	80426048.58	62.27	3.277	28833282.75
2. Spiro-oxazolidinone	48729999.63	37.73	6.35	10569910.38

Yield calculation:

Initial amount of **2a** for defixtion**:** 116.11 mg (=119.7 mg - 3.59 mg), 0.533 mmol

Volume of crude in dioxane: 5 ml

Internal Std. (Naphthalene): 3.24 mg, 0.0253mmol

Volume of **2a** dioxane solution for GC-Ms analysis: 150 µL (contains 3.34 mg of **2a**)

Quantitative estimation of **2a** in crude solution: 111.4 mg, 0.511 mmol

Yield: 96%

Fixation-defixation cycle of CO_2 both at 30 °C using recyclable NaI

At constant temperature (30 ^{0}C) five consecutive cycles of CO_2 fixation and defixation was accomplished using above method (general procedure 3). GC yield in resynthesis of spirooxazolidione **2a** was monitored at each stage.

GC yiled vs no. of cycle plot fo CO_2 fixation and defixation at 30 $^{\circ}$C;

	GC Yield(%)			Standard Deviation	Mean
cycle 1	97	98	97	0.58	97.33
cycle 2	96	95	96	0.58	95.67
cycle 3	99	97	98	1	98
cycle 4	97	95	96	1	96
cycle 5	95	97	96	1	96

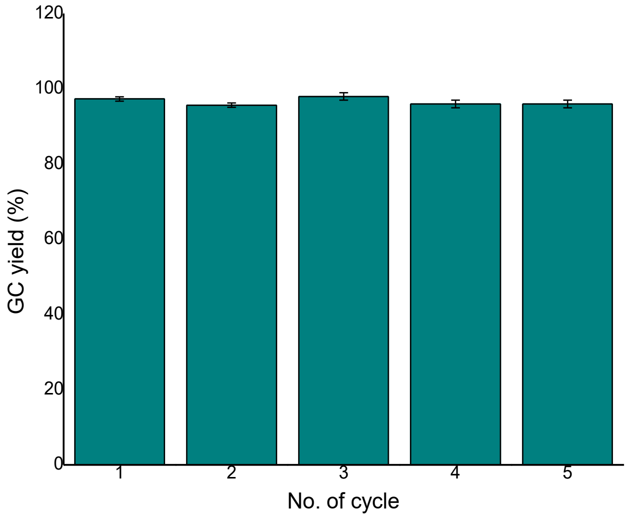

Chemical fixation-defixation cycles at 30 °C

Experimental temperature 70 °C

Trial I(70):

Cycle I-1(70):

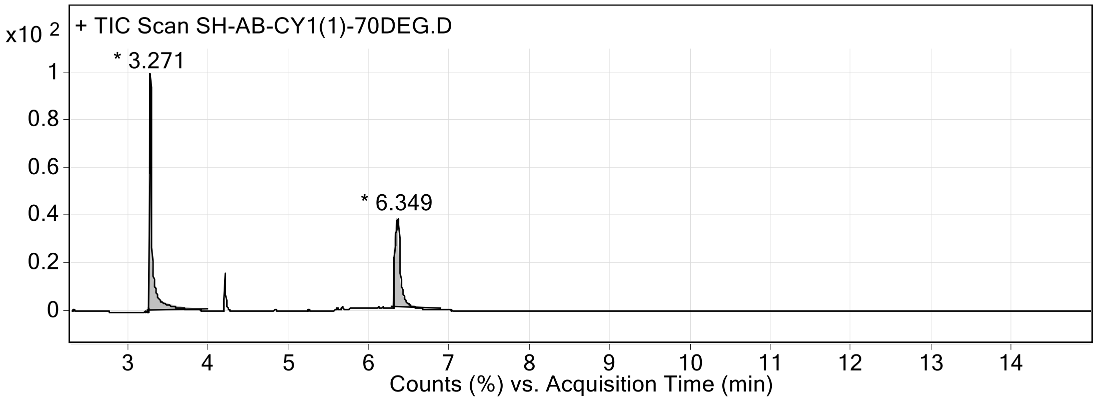

Integration Peak List				
**Peak**	**Area**	**Area Sum Percent**	**RT**	**Height**
1. Naphthalene	62338108.32	59.25	3.271	23198595.16
2. Spiro-oxazolidinone	42878022.12	40.75	6.349	8666552.32

Yield calculation: Initial amount of **2a** for defixtion**:** 150 mg

Volume of crude in dioxane: 5 ml

Internal Std. (Naphthalene): 3.69 mg, 0.0288 mmol

Volume of **2a** dioxane solution for GC-Ms analysis: 150 µL (contains 4.32 mg of **2a**)

Quantitative estimation of **2a** in crude solution: 144.0 mg, 0.660 mmol

Yield: 96%

Cycle I-2(70):

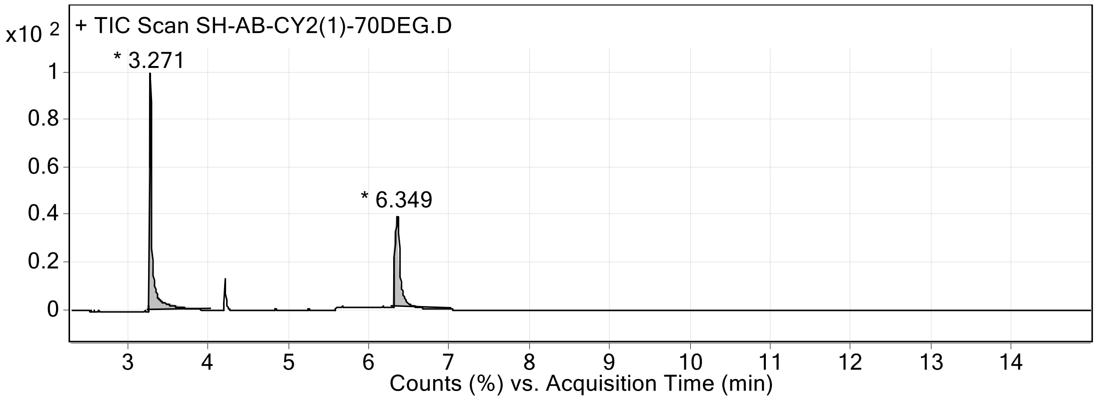

Integration Peak List				
Peak	Area	Area Sum Percent	RT	Height
1. Naphthalene	60427560.55	59.54	3.271	23079063.38
2. Spiro-oxazolidinone	41062505.37	40.46	6.349	8856209.75

Yield calculation: Initial amount of **2a** for defixtion**:** 139.68 mg (=144.0 mg - 4.32 mg), 0.641 mmol

Volume of crude in dioxane: 5 ml

Internal Std. (Naphthalene): 3.55 mg, 0.0277 mmol

Volume of **2a** dioxane solution for GC-Ms analysis: 150 µL (contains 4.10 mg of **2a**)

Quantitative estimation of **2a** in crude solution: 136.8 mg, 0.627 mmol

Yield: 98%

Cycle I-3(70):

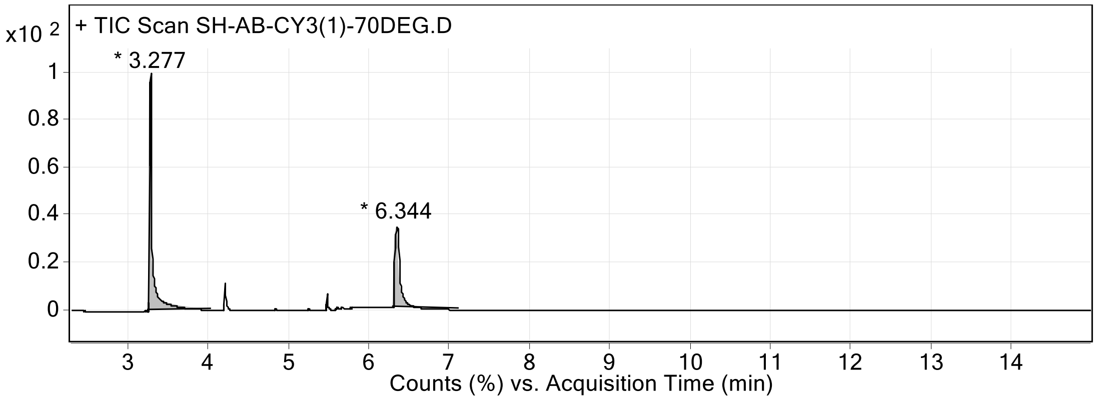

Integration Peak List				
Peak	Area	Area Sum Percent	RT	Height
1. Naphthalene	70709124.62	64.6	3.277	24765258.68
2. Spiro-oxazolidinone	38746389.53	35.4	6.344	8369362.62

Yield calculation: **:** Initial amount of **2a** for defixtion**:** 132.78 mg (=136.89 mg - 4.11 mg), 0.641 mmol

Volume of crude in dioxane: 5 ml

Internal Std. (Naphthalene): 4.14 mg, 0.0323 mmol

Volume of **2a** dioxane solution for GC-Ms analysis: 150 µL (contains 3.86 mg of **2a**)

Quantitative estimation of **2a** in crude solution: 128.6 mg, 0.59 mmol

Yield: 97%

Cycle I-4(70):

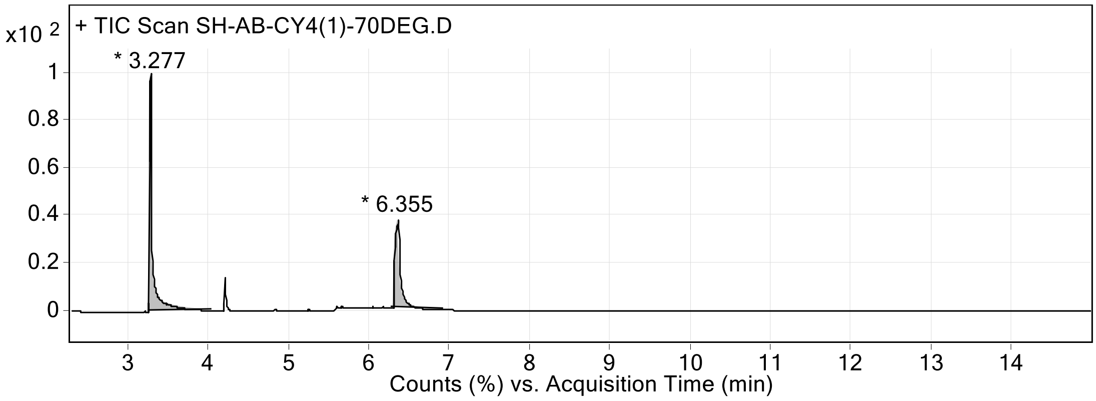

**Integration Peak List**				
**Peak**	**Area**	**Area Sum Percent**	**RT**	**Height**
1. Naphthalene	69413066.26	61.57	3.277	24398156.22
2. Spiro-oxazolidinone	43321231.8	38.43	6.355	8966318.53

Yield calculation: Initial amount of **2a** for defixtion**:** 124.94 mg (=128.8 mg - 3.86 mg), 0.641 mmol

Volume of crude in dioxane: 5 ml

Internal Std. (Naphthalene): 3.314 mg, 0.0259 mmol

Volume of **2a** dioxane solution for GC-Ms analysis: 150 µL (contains 3.52 mg of **2a**)

Quantitative estimation of **2a** in crude solution: 117.5 mg, 0.539mmol

Yield: 94%

Cycle I-5(70):

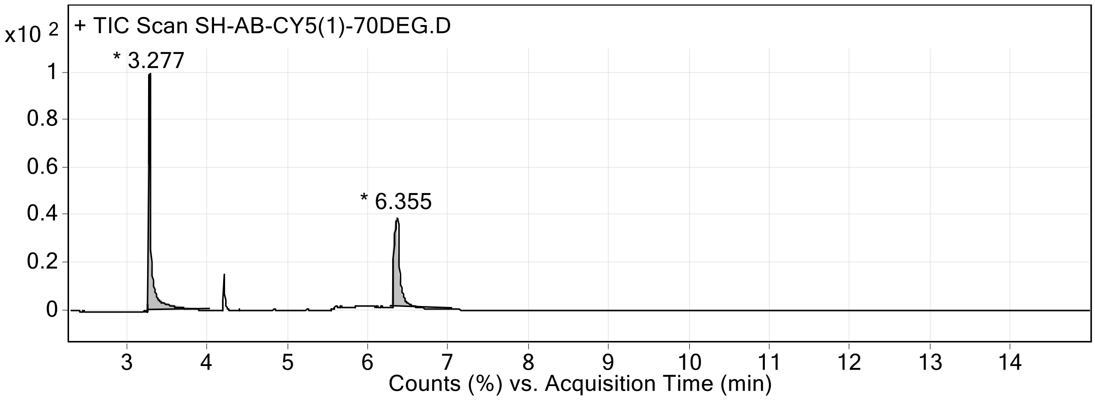

**Integration Peak List**				
**Peak**	**Area**	**Area Sum Percent**	**RT**	**Height**
1. Naphthalene	62423792.79	58.73	3.277	22606241.65
2. Spiro-oxazolidinone	43857733.95	41.27	6.355	8510821.61

Yield calculation: Initial amount of **2a** for defixtion**:** 113.92 mg (=117.44 mg - 3.52 mg), 0.641 mmol

Volume of crude in dioxane: 5 ml

Internal Std. (Naphthalene): 2.741 mg, 0.0214mmol

Volume of **2a** dioxane solution for GC-Ms analysis: 150 µL (contains 3.28 mg of **2a**)

Quantitative estimation of **2a** in crude solution: 109.3 mg, 0.501 mmol

Yield: 96%

Trial II(70):

Cycle II-1(70):

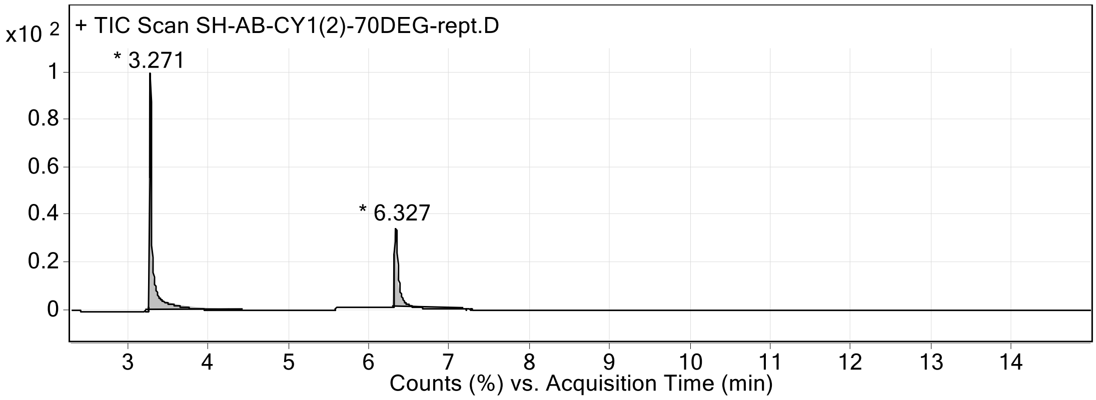

Integration Peak List

**Peak**	**Area**	**Area Sum Percent**	**RT**	**Height**
1. Naphthalene	63940832.3	70.19	3.271	22080535.46
2. Spiro-oxazolidinone	27154156.85	29.81	6.327	7266189.58

Yield calculation: Initial amount of **2a** for defixtion**:** 150 mg, 0.0688 mmol

Volume of crude in dioxane: 5 ml

Internal Std. (Naphthalene): 5.91 mg, 0.0462 mmol

Volume of **2a** dioxane solution for GC-Ms analysis: 150 μL (contains 4.28 mg of **2a**)

Quantitative estimation of **2a** in crude solution: 142.6 mg, 0.654 mmol

Yield: 95%

Cycle II-2(70):

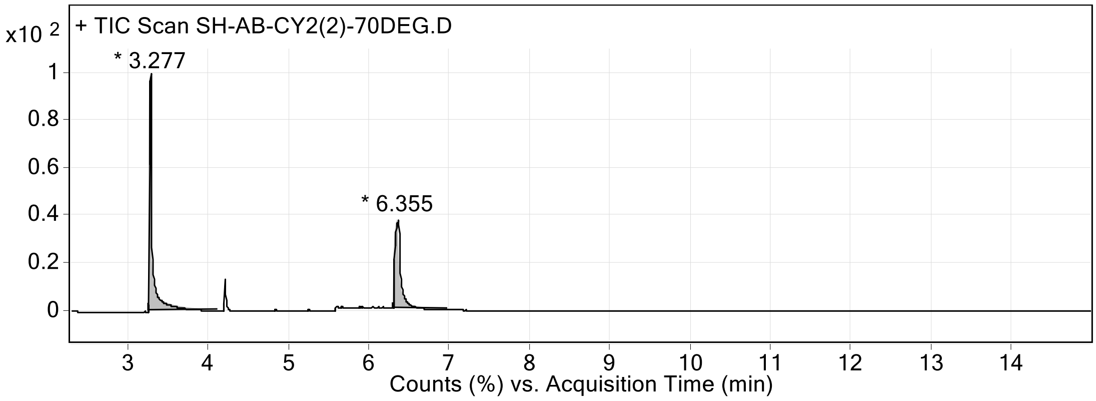

Integration Peak List

Peak	Area	Area Sum Percent	RT	Height
1. Naphthalene	69371056.27	59.78	3.277	24319131.75
2. Spiro-oxazolidinone	46671549.31	40.22	6.355	9075990.19

Yield calculation: Initial amount of **2a** for defixtion**:** 138.22 mg (=142.5 mg - 4.28 mg), 0.634 mmol

Volume of crude in dioxane: 5 ml

Internal Std. (Naphthalene): 3.49 mg, 0.0273 mmol

Volume of **2a** dioxane solution for GC-Ms analysis: 150 µL (contains 4.00 mg of **2a**)

Quantitative estimation of **2a** in crude solution: 133.3 mg, 0.612 mmol

Yield: 96%

Cycle II-3(70):

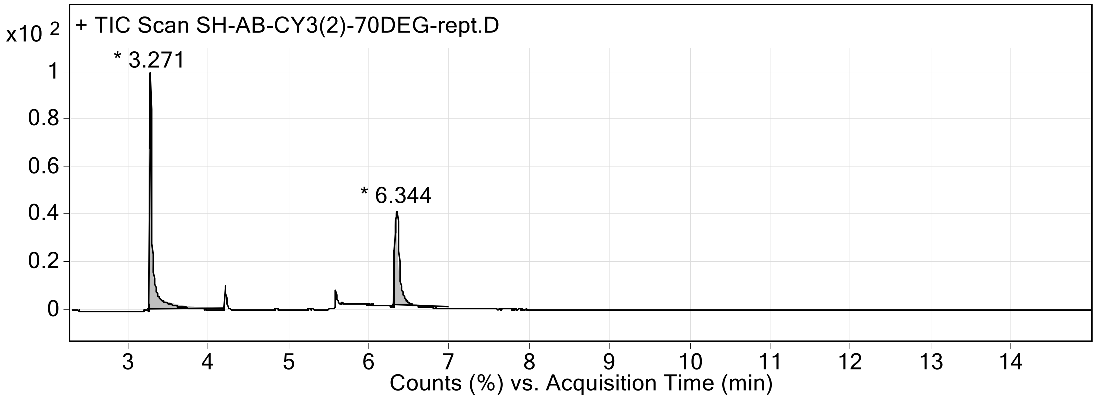

Integration Peak List

Peak	Area	Area Sum Percent	RT	Height
1. Naphthalene	56521739.71	61.81	3.271	20392113.31
2. Spiro-oxazolidinone	34929223.92	38.19	6.344	8093252.18

Yield calculation Initial amount of **2a** for defixtion**:** 129.3 mg (=133.33 mg - 4.00 mg), 0.593 mmol

Volume of crude in dioxane: 5 ml

Internal Std. (Naphthalene): 3.46 mg, 0.0271 mmol

Volume of **2a** dioxane solution for GC-Ms analysis: 150 µL (contains 3.65 mg of **2a**)

Quantitative estimation of **2a** in crude solution: 121.7 mg, 0.558 mmol

Yield: 94%

Cycle II-4(70):

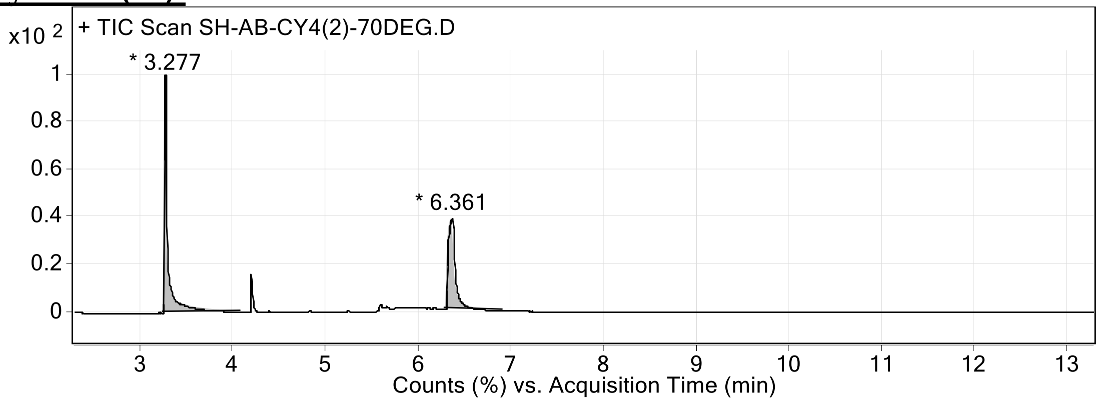

Integration Peak List

Peak	Area	Area Sum Percent	RT	Height
1. Naphthalene	65855089.34	58.1	3.277	23341460.44
2. Spiro-oxazolidinone	47494199.12	41.9	6.361	8875383.95

Yield calculation Initial amount of **2a** for defixtion**:** 117.87 mg (=121.52 mg - 3.65mg), 0.541 mmol

Volume of crude in dioxane: 5 ml

Internal Std. (Naphthalene): 2.79 mg, 0.0218 mmol

Volume of **2a** dioxane solution for GC-Ms analysis: 150 µL (contains 3.43 mg of **2a**)

Quantitative estimation of **2a** in crude solution: 114.2 mg, 0.524 mmol

Yield: 97%

Cycle II-5(70):

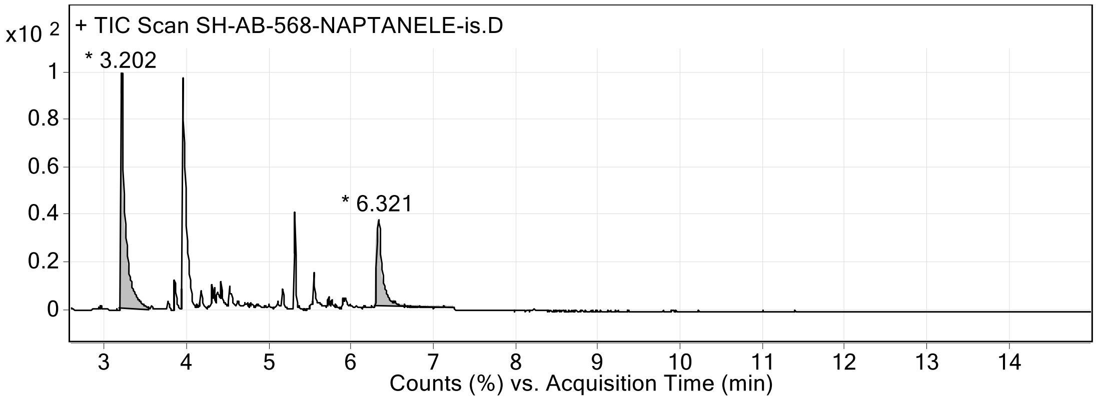

Integration Peak List

**Peak**	**Area**	**Area Sum Percent**	**RT**	**Height**
1. Naphthalene	6913695.25	63.32	3.941	2198375.45
2. Spiro-oxazolidinone	4004995.22	36.68	6.328	709837.99

Yield calculation Initial amount of **2a** for defixtion**:** 110.9 mg (=114.33 mg - 3.43 mg), 0.509 mmol

Volume of crude in dioxane: 5 ml

Internal Std. (Naphthalene): 3.23 mg, 0.0252 mmol

Volume of **2a** dioxane solution for GC-Ms analysis: 150 µL (contains 3.18 mg of **2a**)

Quantitative estimation of **2a** in crude solution: 106.1 mg, 0.487 mmol

Yield: 96%

Trial III(70):

Cycle III-1(70):

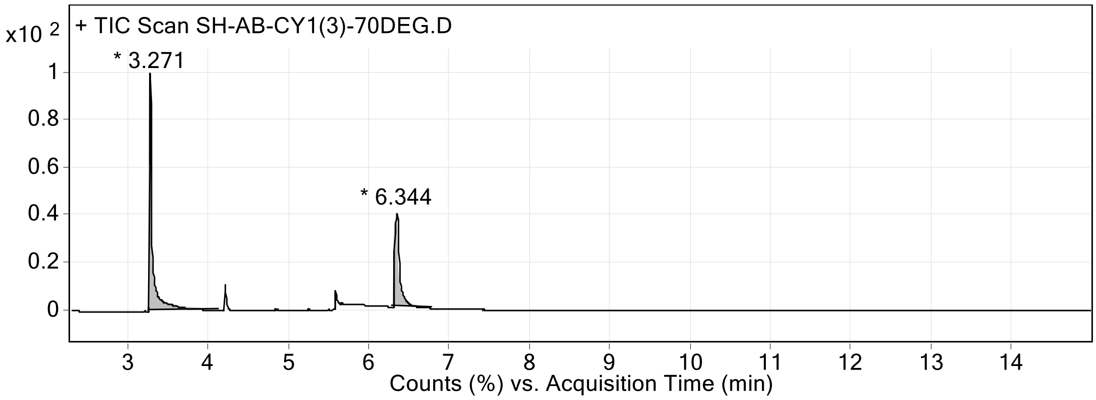

Integration Peak List

Peak	Area	Area Sum Percent	RT	Height
1. Naphthalene	59368654.48	61.89	3.271	21546655.44
2. Spiro-oxazolidinone	36549873.43	38.11	6.344	8479997.47

Yield calculation Initial amount of **2a** for defixtion**:** 150 mg, 0.688 mmol

Volume of crude in dioxane: 5 ml

Internal Std. (Naphthalene): 4.033 mg, 0.0315 mmol

Volume of **2a** dioxane solution for GC-Ms analysis: 150 µL (contains 4.23 mg of **2a**)

Quantitative estimation of **2a** in crude solution: 141 mg, 0.648 mmol

Yield: 94%

Cycle III-2(70):

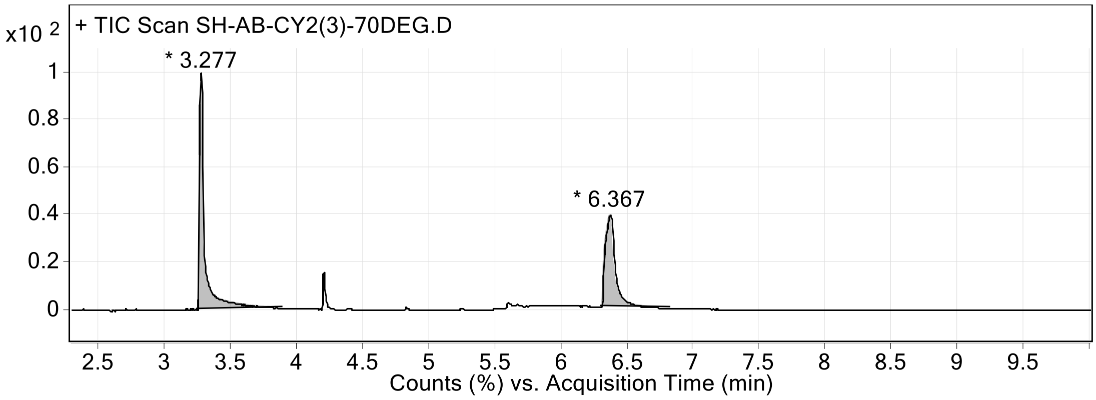

Integration

Peak List				
**Peak**	**Area**	**Area Sum Percent**	**RT**	**Height**
1. Naphthalene	70013679.24	57.99	3.277	24016537.25
2. Spiro-oxazolidinone	50712454.57	42.01	6.367	9284020.98

Yield calculation: Initial amount of **2a** for defixtion**:** 136.77 mg (=141 mg - 4.23 mg), 0.627 mmol

Volume of crude in dioxane: 5 ml

Internal Std. (Naphthalene): 3.22 mg, 0.0252 mmol

Volume of **2a** dioxane solution for GC-Ms analysis: 150 µL (contains 3.98 mg of **2a**)

Quantitative estimation of **2a** in crude solution: 132.7 mg, 0.608 mmol

Yield: 97%

Cycle III-3(70):

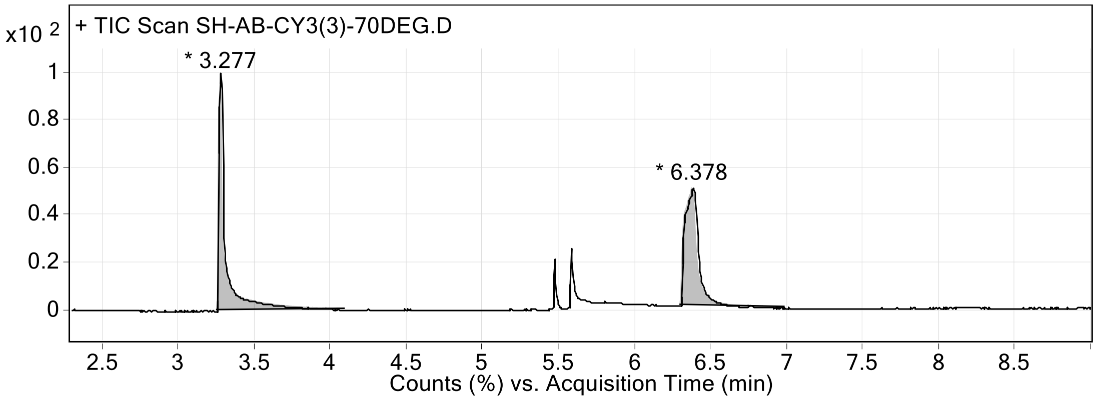

Integration
Peak List

**Peak**	**Area**	**Area Sum Percent**	**RT**	**Height**
1. Naphthalene	77772193.75	51.14	3.277	23680497.99
2. Spiro-oxazolidinone	74296205.04	48.86	6.378	11705644.92

Yield calculation: Initial amount of **2a** for defixtion**:** 128.79 mg (=132.67 mg - 3.98 mg), 0.591 mmol

Volume of crude in dioxane: 5 ml

Internal Std. (Naphthalene): 2.279 mg, 0.0178 mmol

Volume of **2a** dioxane solution for GC-Ms analysis: 150 µL (contains 3.71 mg of **2a**)

Quantitative estimation of **2a** in crude solution: 123.6 mg, 0.567 mmol

Yield: 96%

<u>Cycle III-4(70):</u>

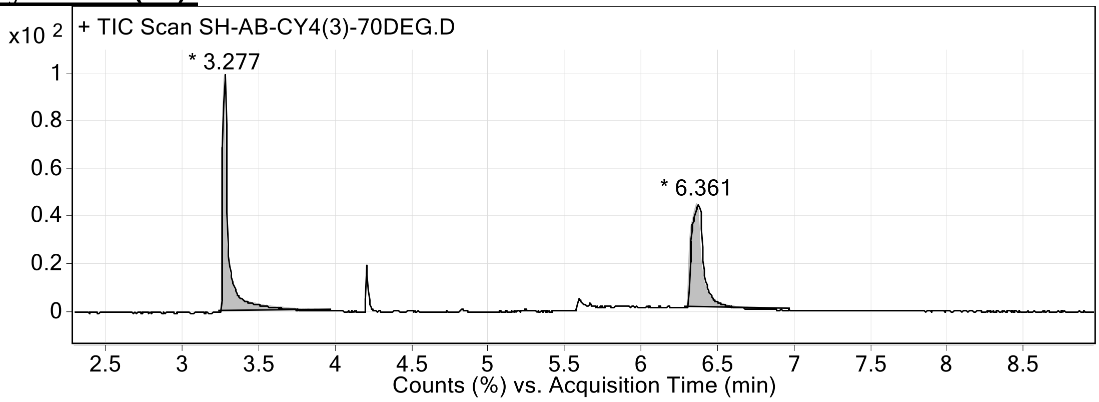

Integration Peak List

**Peak**	**Area**	**Area Sum Percent**	**RT**	**Height**
1. Naphthalene	66721485.55	53.68	3.277	23269727.64
2. Spiro-oxazolidinone	57569015.44	46.32	6.361	10168689.95

<u>Yield calculation:</u> Initial amount of **2a** for defixtion**:** 119.97 mg (=123.68 mg - 3.71 mg), 0.55 mmol

Volume of crude in dioxane: 5 ml

Internal Std. (Naphthalene): 2.4 mg, 0.0188 mmol

Volume of **2a** dioxane solution for GC-Ms analysis: 150 µL (contains 3.53 mg of **2a**)

Quantitative estimation of **2a** in crude solution: 117.9 mg, 0.540 mmol

Yield: 98 %

Cycle III-5(70):

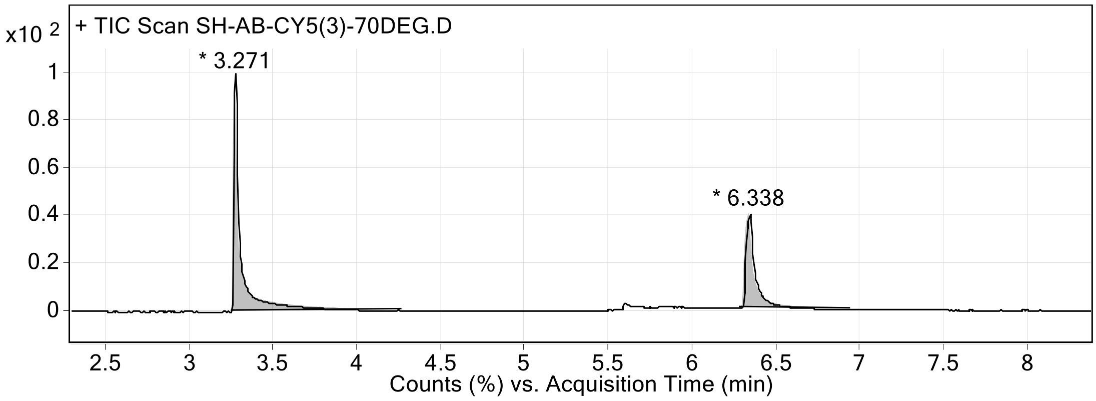

Integration Peak List

**Peak**	**Area**	**Area Sum Percent**	**RT**	**Height**
1. Naphthalene	63275089.23	65.15	3.271	21676187.7
2. Spiro-oxazolidinone	33841420.2	34.85	6.338	8533938.52

Yield calculation: Initial amount of **2a** for defixtion**:** 114.04 mg (=117.57 mg - 3.53 mg), 0.523 mmol

Volume of crude in dioxane: 5 ml

Internal Std. (Naphthalene): 3.57 mg, 0.0279 mmol

Volume of **2a** dioxane solution for GC-Ms analysis: 150 µL (contains 3.25 mg of **2a**)

Quantitative estimation of **2a** in crude solution: 108.4 mg, 0.497 mmol

Yield: 95 %

GC yield vs no. of cycle plot fo CO_2 fixation and defixation at 70 ^{o}C

		GC yield(%)		Standard Deviation	Mean
cycle1	96	95	94	0.816497	95
cycle2	98	96	97	0.816497	97
cycle 3	97	94	96	1.247219	95.67
cycle 4	94	97	98	1.699673	96.33
cycle 5	96	96	95	0.471405	95.67

At constant temperature (70 ^{0}C) five consecutive cycles of CO_2 fixation and defixation was accomplished using above method (general procedure 3). GC yield in resynthesis of spirooxazolidione **2a** was monitored at each stage.

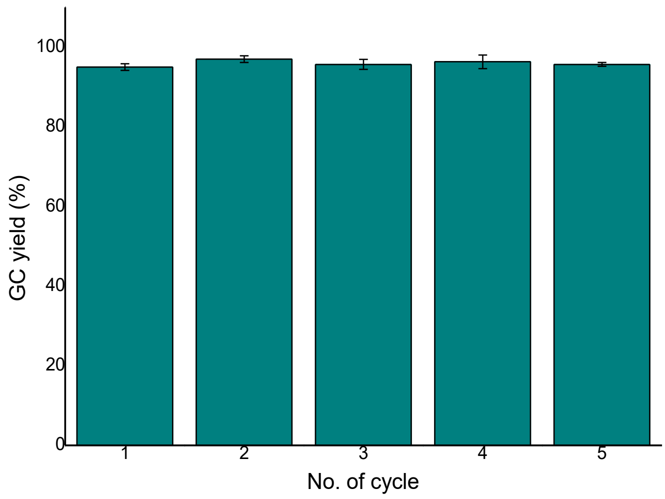

Chemical fixation-defixation cycles at 70 °C

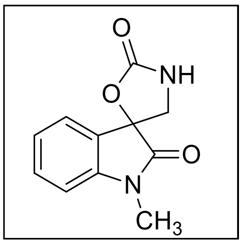

1-Methylspiro[indoline-3,5'-oxazolidine]-2,2'-dione (2a): White solid (528 mg, 93% isolated yield, 98% GC yield using flue gas); Melting point 178-180 °C; ^{1}H NMR (400 MHz, $CDCl_3$) δ 7.49 (d, *J* = 7.4 Hz, 1H), 7.42 (t, *J* = 7.8 Hz, 1H), 7.16 (t, *J* = 7.6 Hz, 1H), 6.87 (d, *J* = 7.8 Hz, 1H), 6.01 – 5.86 (m, 1H), 3.98 (d, *J* = 9.0 Hz, 1H), 3.76 (d, *J* = 9.0 Hz, 1H), 3.22 (s, 3H).^{13}C NMR (100 MHz, d_6-DMSO) δ 173.4, 158.0, 144.4, 131.7, 126.7, 125.1, 123.7, 109.8, 78.8, 47.7, 26.7; HRMS (ESI) calcd for $C_{11}H_{11}N_2O_3$ [M+H]+ = 219.0770, found 219.0767.

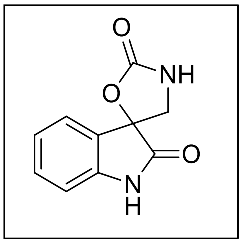

Spiro[indoline-3,5'-oxazolidine]-2,2'-dione (2b): Pale yellow solid (32.3 mg, 94% yield, 96% GC yield using flue gas); Melting point 235-237 °C; ^{1}H NMR (400 MHz, DMSO-d_6) δ 10.68 (s, 1H), 8.08 (s, 1H), 7.51 (d, *J* = 7.3 Hz, 1H), 7.35 (td, *J* = 7.7, 1.3 Hz, 1H), 7.07 (td, *J* = 7.6, 1.0 Hz, 1H), 6.89 (d, *J* = 7.7 Hz, 1H), 3.74 (dd, *J* = 9.7, 1.0 Hz, 1H), 3.67 (d, *J* = 9.6 Hz, 1H); ^{13}C NMR (100 MHz, DMSO-d_6) δ 175.2, 158.1, 142.9, 131.7, 127.2, 125.5, 123.1, 110.8, 79.1, 47.7;HRMS (ESI) calcd for $C_{10}H_9N_2O_3$ [M+H]+ =205.0613, found 205.0603.

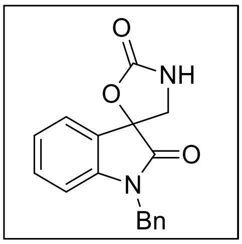

1-Benzylspiro[indoline-3,5'-oxazolidine]-2,2'-dione (2c): Pale yellow solid (25.9 mg, 79% yield); Melting point 168-170 °C; ^{1}H NMR (400 MHz, DMSO-d_6) δ 8.16 (s, 1H), 7.59 (dd, *J* = 7.4, 1.3 Hz, 1H), 7.41 – 7.24 (m, 6H), 7.12 (td, *J* = 7.5, 0.9 Hz, 1H), 6.97 (d, *J* = 7.8 Hz, 1H), 4.93 (d, *J* = 15.8 Hz, 1H), 4.84 (d, *J* = 15.8 Hz,1H), 3.81 (d, *J* = 9.8 Hz,1H), 3.75 (d, *J* = 9.7 Hz, 1H);^{13}C NMR (100 MHz, DMSO) δ 173.7, 158.0, 143.4, 136.2, 131.7, 129.2,

128.1, 127.7, 126.6, 125.4, 123.9, 110.4, 78.8, 47.7, 43.3; HRMS (ESI) calcd for $C_{17}H_{15}N_2O_3$ [M+H]+ = 295.1083, found 295.1077.

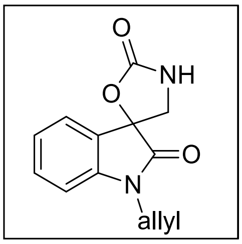

1-Allylspiro[indoline-3,5'-oxazolidine]-2,2'-dione (2d): Pale yellow solid (30.9 mg, 92% yield); Melting point 144-146 °C; ^{1}H NMR (400 MHz, DMSO-d_6) δ 8.12 (s, 1H), 7.58 (d, *J* = 7.3 Hz, 1H), 7.41 (t, *J*= 7.7 Hz, 1H), 7.14 (t, *J* = 7.5 Hz, 1H), 7.02 (d, *J* = 7.9 Hz, 1H), 5.83 (ddd, *J* = 21.2, 9.9, 5.1 Hz, 1H), 5.19 (s,, 1H), 5.16 (d, *J* = 5.8Hz, 1H), 4.33 – 4.22 (m, 2H), 3.75 (d, *J* = 9.8 Hz, 1H), 3.71 (d, *J* = 9.7 Hz, 1H); ^{13}C NMR (100 MHz, DMSO-d_6) δ 173.3, 158.0, 143.5, 131.9, 131.7, 126.6, 125.3, 123.8, 117.7, 110.4, 78.7, 47.7, 42.2; HRMS (ESI) calcd for $C_{13}H_{13}N_2O_3$ [M+H] + = 245.0926, found 245.0920.

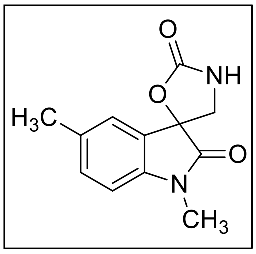

1,5-Dimethylspiro[indoline-3,5'-oxazolidine]-2,2'-dione (2e): Pale yellow solid (30.7 mg, 91% yield, 94% GC yield using flue gas); Melting point 227-229 °C; ^{1}H NMR (400 MHz, DMSO-d_6) δ 8.09 (s, 1H), 7.39 (s, 1H), 7.25 (d, *J* = 7.8, 1H), 6.97 (d, *J* = 7.9 Hz, 1H), 3.72 (d, *J* = 9.7 Hz, 1H), 3.67 (d, *J* = 9.7 Hz, 1H), 3.11 (s, 3H), 2.31 (s, 3H); ^{13}C NMR (100 MHz, DMSO-d_6) δ 173.3, 158.0, 142.0, 133.0, 131.8, 126.7, 125.6, 109.6, 78.9, 47.8, 26.7, 21.0; HRMS (ESI) calcd for $C_{12}H_{13}N_2O_3$ [M+H]+ = 233.0926, found 233.0923.

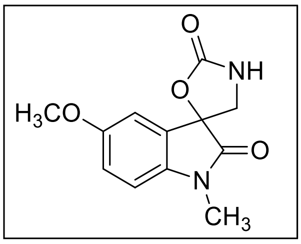

5-Methoxy-1-methylspiro[indoline-3,5'-oxazolidine]-2,2'-dione (2f): Pale yellow solid (30.2 mg, 90% yield, 93% GC yield using flue gas); Melting point 185-187 °C; ^{1}H NMR

(400 MHz, $CDCl_3$) δ 7.10 (d, *J* = 2.5 Hz, 1H), 6.96 (dd, *J* = 8.5, 2.6 Hz, 1H), 6.80 (d, *J* = 8.5 Hz, 1H), 5.53 (br, 1H), 4.00 (dd, *J* = 8.9, 1.1 Hz, 1H), 3.84 (s, 3H), 3.76 (d, *J* = 8.9 Hz, 1H), 3.22 (s, 3H); ^{13}C NMR (100 MHz, DMSO-d_6) δ 173.2, 158.0, 156.6, 137.6, 127.6, 116.5, 112.0, 110.5, 79.1, 56.2, 47.7, 26.7; HRMS (ESI) calcd for $C_{12}H_{13}N_2O_4$ [M+H]+ = 249.0875, found 249.0868.

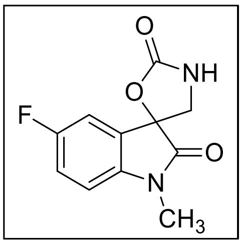

5-Fluoro-1-methylspiro[indoline-3,5'-oxazolidine]-2,2'-dione (2g): White solid (26.3 mg, 78% yield); Melting point 60-62 °C; ^1H NMR (400 MHz, $CDCl_3$) δ 7.29 (dd, *J* = 7.2, 2.5 Hz, 1H), 7.14 (td, *J* = 8.8, 2.5 Hz, 1H), 6.84-6.80 (m, 1H), 6.61 (s, 1H), 4.00 (d, *J* = 9.2 Hz, 1H), 3.78 (d, *J* = 9.2 Hz, 1H), 3.23 (s, 3H); ^{13}C NMR (100 MHz, $CDCl_3$) δ 172.6, 160.9, 158.5 (d, *J* = 14.2 Hz, 1C), 139.8, 127.6, 117.8 (d, *J* = 23.5 Hz, 1C), 112.8 (d, *J* = 25.3 Hz, 1C), 109.8 (d, *J* = 8.1 Hz, 1C), 79.1, 48.2, 26.7; HRMS (ESI) calcd for $C_{11}H_{10}FN_2O_3$[M+H]+ = 237.0675, found 237.0675.

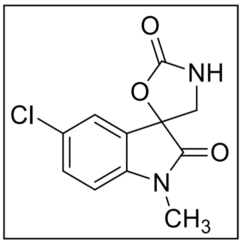

5-Chloro-1-methylspiro[indoline-3,5'-oxazolidine]-2,2'-dione (2h): Pale yellow solid (27.8 mg, 83% yield); Melting point 166-168 °C; ^1H NMR (400 MHz, DMSO-d_6) δ 8.12 (s, 1H), 7.73 (d, *J* = 2.1 Hz, 1H), 7.50 (dd, *J* = 8.4, 2.2 Hz, 1H), 7.10 (d, *J* = 8.4 Hz, 1H), 3.73 (d, *J* = 9.5 Hz, 1H), 3.70 (dd, *J* = 9.8, 0.8 Hz, 1H), 3.11 (s, 3H);^{13}C NMR (100 MHz, DMSO-d_6) δ 173.2, 157.8, 143.5, 131.5, 128.5, 127.7, 125.6, 111.4, 78.5, 47.5, 26.9; HRMS (ESI) calcd for $C_{11}H_{10}ClN_2O_3$[M+H]+ = 253.0380, found 253.0372.

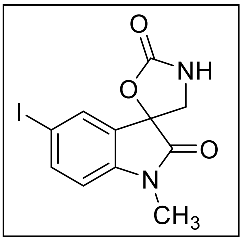

5-Iodo-1-methylspiro[indoline-3,5'-oxazolidine]-2,2'-dione (2i): Pale yellow solid (28.6 mg, 88% yield); Melting point 213-215 °C; ^{1}H NMR (400 MHz, DMSO-d_6) δ 8.12 (s, 1H), 7.94 (d, J = 1.8 Hz, 1H), 7.80 (dd, J = 8.2, 1.8 Hz, 1H), 6.94 (d, J = 8.2 Hz, 1H), 3.74 (d, J = 9.7 Hz, 1H), 3.70 (d, J = 9.7 Hz, 1H), 3.11 (s, 3H); ^{13}C NMR (100 MHz, DMSO) δ 172.9, 157.8, 144.3, 140.1, 133.5, 129.0, 112.3, 86.5, 78.3, 47.5, 26.8; HRMS (ESI) calcd for $C_{11}H_{10}IN_2O_3$ [M+H]+ = 344.9736, found 344.9730.

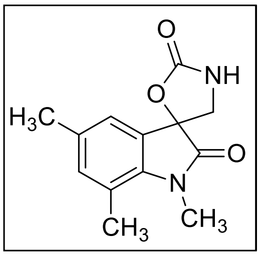

1,5,7-trimethylspiro[indoline-3,5'-oxazolidine]-2,2'-dione (2j): Pale yellow solid (32.2 mg, 96% yield, 92% GC yield using flue gas); Melting point 209-211 °C;^{1}H NMR (400 MHz, DMSO-d_6) δ 8.06 (s, 1H), 7.19 (s, 1H), 6.99 (s, 1H), 3.67 (d, J = 9.6 Hz, 1H), 3.60 (d, J = 9.6 Hz, 1H), 3.35 (s, 3H), 2.47 (s, 3H), 2.23 (s, 3H); ^{13}C NMR (100 MHz, DMSO-d_6) δ 173.9, 158.0, 139.4, 135.4, 132.9, 127.5, 123.4, 120.9, 78.4, 48.1, 29.7, 20.7, 18.6; HRMS (ESI) calcd for $C_{13}H_{15}N_2O_3$ [M+H]+ = 247.1083, found 247.1079.

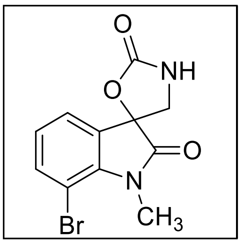

7-bromo-1-methylspiro[indoline-3,5'-oxazolidine]-2,2'-dione (2k): Pale yellow solid (30.8 mg, 94% yield); Melting point 210-212 °C; ^{1}H NMR (400 MHz, DMSO-d_6) δ 8.15 (s, 1H), 7.61 (s, 1H), 7.59 (s, 1H), 7.08 (t, J = 7.8 Hz, 1H), 3.75 (d, J = 9.8 Hz, 1H), 3.67 (d, J = 9.9 Hz, 1H), 3.46 (s, 3H); ^{13}C NMR (100 MHz, DMSO-d_6) δ 174.1, 157.7, 141.6, 136.9, 130.2,

125.5, 124.7, 102.6, 78.0, 48.1, 30.2; HRMS (ESI) calcd for $C_{11}H_{10}BrN_2O_3$ [M+H]+ = 296.9875, found 296.9870.

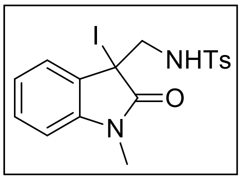

***N*-(3-Iodo-1-methyl-2-oxoindolin-3-yl)methyl)-4-methylbenzenesulfonamide (5a):** To a stirred solution of spirooxazolidinone **2a** (50 mg, 0.23 mmol) in dry dioxane (2 ml), sodium iodide (138 mg, 0.92 mmol) and *o*-phosphoric acid (48 μL, 0.92 mmol) was added at 70 °C. After 5 h, reaction mixture was cooled to room temperature and Et_3N (401 μL, 2.88 mmol) and TsCl (52 mg, 0.27 mmol) were successively added. The reaction mixture was stirred for 3 h and dioxane was removed. The product **5a** was isolated as a colourless gummy oil (92 mg, 88%) through purification in silica gel flash chromatography using hexanes-EtOAc (10:3).

^{1}H NMR (400 MHz, $CDCl_3$) δ 7.65 (d, *J* = 8.0 Hz, 2H), 7.37 (d, *J* = 7.4 Hz, 1H), 7.29 (t, *J* = 7.8 Hz, 1H), 7.23-7.18 (m, 2H), 7.04 (t, *J* = 7.6 Hz, 1H), 6.76 (d, *J* = 7.8 Hz, 1H), 5.43 (d, *J* = 9.6 Hz, 1H), 3.42 (dd, *J* = 12.9, 9.8 Hz, 1H), 3.08 (s, 3H), 3.02 (dd, *J* = 13.1, 3.3 Hz, 1H), 2.34 (s, 3H).^{13}C NMR (100 MHz, $CDCl_3$) δ 176.4, 143.8, 143.3 , 136.5 , 130.5 , 129.8 , 127.4, 127.1, 124.5, 123.6, 108.8, 73.5, 49.1, 26.3, 21.5; HRMS (ESI) calcd for $C_{17}H_{18}IN_2O_3S$ [M+H]+ = 457.0083, found 457.0076.

Note: The white iodoammonium salt **4a** was found to be sparingly soluble in d_6-DMSO, therefore spectral characterization has been done after *N*-tosyl protection.

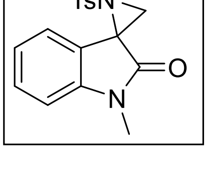

Procedure for isolation of 1'-methyl-1-tosylspiro[aziridine-2,3'-indolin]-2'-one (6a): To a solution of spirooxazolidinone **2a** (150 mg, 0.69 mmol) in dry dioxane (6 ml), sodium iodide (414 mg, 2.76 mmol) and *o*-phosphoric acid (144 μL, 2.76 mmol) were added. The mixture was stirred at 70 °C and the consumption of **2a** was monitored by TLC and GC-MS. After complete consumption of the substrate (monitored by TLC), TsCl (461.27 mg, 2.42 mmol) was added into the reaction mixture followed by portion wise addition of $LiOH.H_2O$ to

quench the acid and stirred for 2 h at 0°C. After completion of the reaction, aqueous solution was extracted with ethyl acetate (3 x 20 ml). The combine organic layer was washed thoroughly with brine solution and dried over Na_2SO_4 and removed under reduced pressure to obtain the crude mixture. The residue was purified by flash column chromatography with hexanes: ethyl acetate (5:1) as eluent to give **3a** (124 mg; 55% yield). Yellow solid; Melting point: 124-126 °C; ^{1}H NMR (400 MHz, $CDCl_3$) δ 7.85 (d, *J* = 8.1 Hz, 2H), 7.76 (d, *J* = 7.6 Hz, 1H), 7.40 (t, *J* = 7.7 Hz, 1H), 7.32 (d, *J* = 8.0 Hz, 2H), 7.14 (t, *J* = 7.6 Hz, 1H), 6.92 (d, *J* = 7.9 Hz, 1H), 3.46 (s, 1H), 3.28 (s, 3H), 3.12 (s, 1H), 2.44 (s, 3H).; ^{13}C NMR (101 MHz, $CDCL_3$) δ 169.9, 145.1, 144.7, 136.5, 130.2, 129.7, 127.9, 125.4, 123.0, 120.4, 108.9, 48.2, 40.3, 26.9, 21.7.; HRMS (ESI-TOF): calcd for $C_{17}H_{17}N_2O_3S$ [M+H]+ = 329.0960, found 329.0950.;

Reference

1 Chouhan, M., Senwar, K. R., Sharma, R., Grover, V., Nair, V. A. Regiospecific epoxide opening: a facile approach for the synthesis of 3-hydroxy-3-aminomethylindolin-2-one derivatives *Green Chem.*, **13**, 2553-2560 (2011).

X-ray crystal structure details:

Datablock for the compound **2g**

The details of crystal data collection and refinement of **2g** are summarized in Table 3. CCDC 1898609 contains the supplementary crystallographic data for this paper. This data can be obtained free of charge from The Cambridge Crystallography Data Centre via www.ccdc.cam.ac.uk/data_request/cif.

Datablock: shelx

```
Bond precision:   C-C = 0.0079 A          Wavelength=0.71073

Cell:              a=5.2646(14)      b=13.805(4)      c=14.818(4)
                   alpha=90          beta=90          gamma=90
Temperature:       293 K

                 Calculated                 Reported
Volume           1076.9(5)                  1077.0(5)
Space group      P 21 21 21                 P 21 21 21
Hall group       P 2ac 2ab                  P 2ac 2ab
Moiety formula   C11 H9 F N2 O3             ?
Sum formula      C11 H9 F N2 O3             C11 H9 F N2 O3
Mr               236.20                     236.20
Dx,g cm-3        1.457                      1.457
Z                4                          4
Mu (mm-1)        0.119                      0.119
F000             488.0                      488.0
F000'            488.30
h,k,lmax         7,19,20                    7,18,19
Nref             3140[ 1835]                2780
Tmin,Tmax        0.974,0.979
Tmin'            0.974

Correction method= Not given

Data completeness= 1.51/0.89       Theta(max)= 29.975

R(reflections)= 0.0839( 1443)      wR2(reflections)= 0.2103( 2780)

S = 1.027                  Npar= 154
```

The following ALERTS were generated. Each ALERT has the format
test-name_ALERT_alert-type_alert-level.
Click on the hyperlinks for more details of the test.

ORTEP diagram of **2g**:

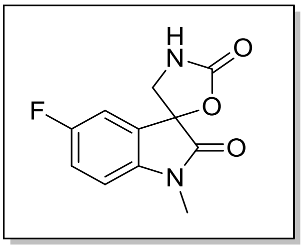

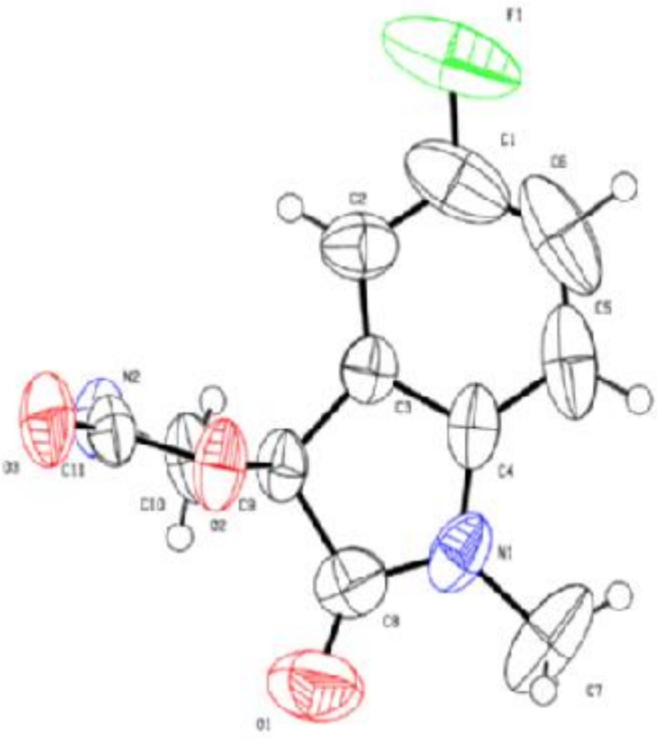

ORTEP drawing of compound **2g** showing thermal ellipsoids at the 50% probability level.

NMR spectra:

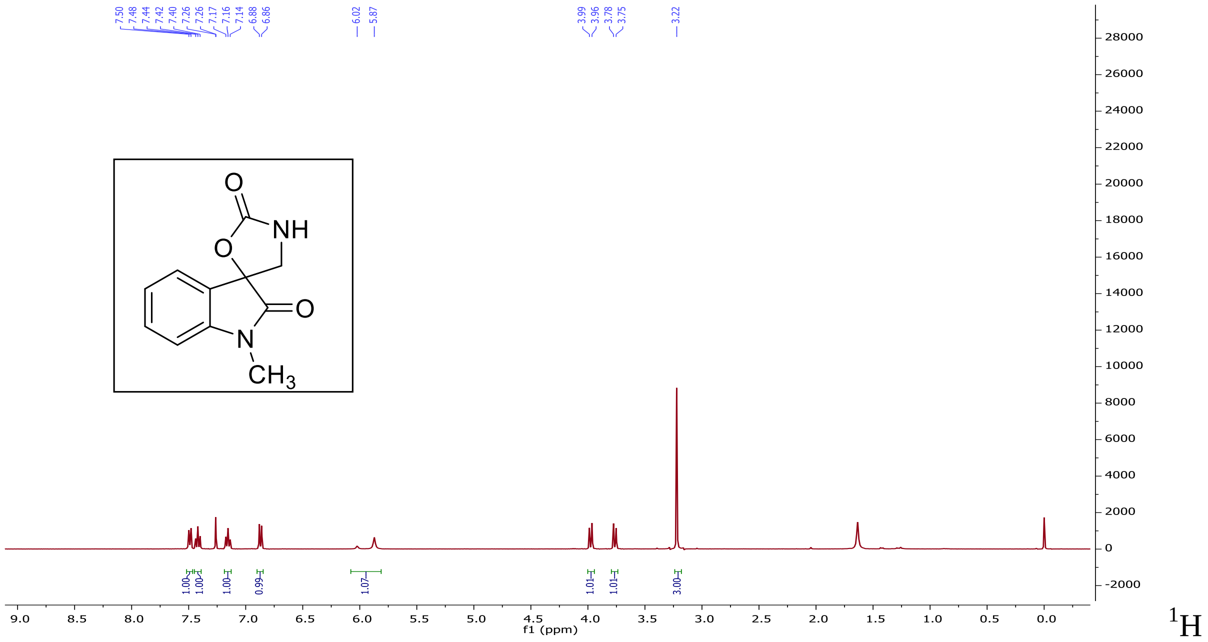

NMR spectrum of compound **2a** (400 MHz, $CDCl_3$)

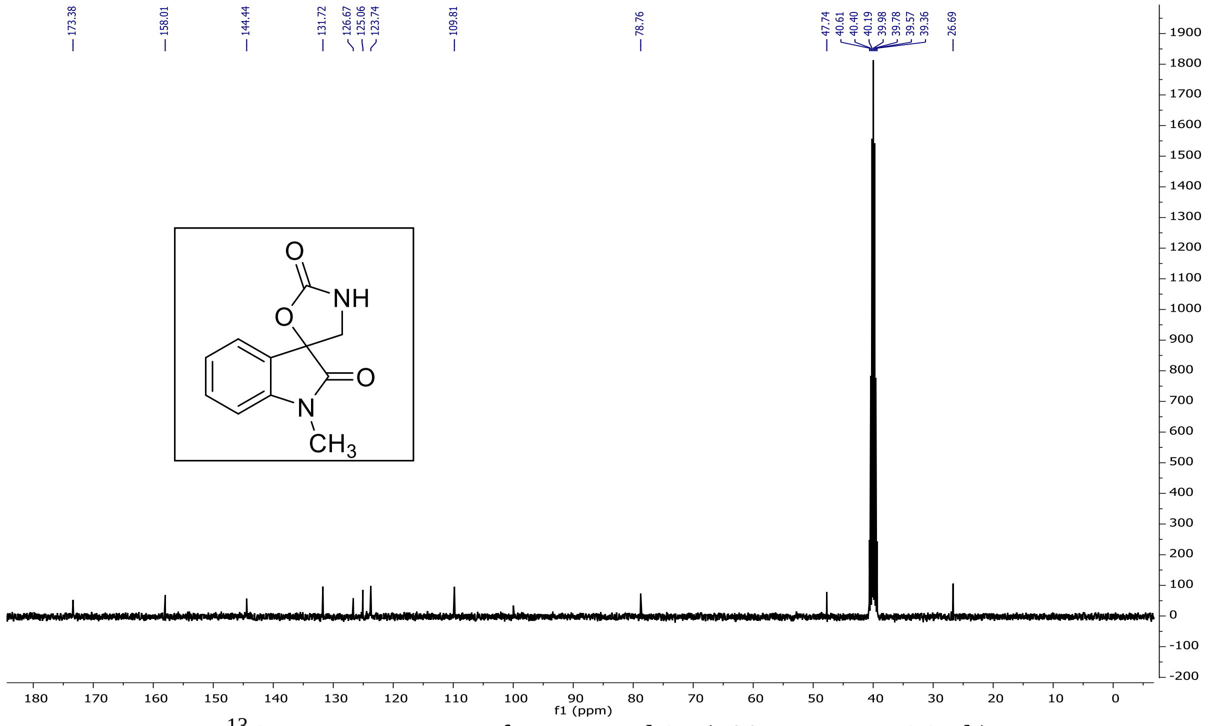

^{13}C NMR spectrum of compound **2a** (100 MHz, DMSO-d_6)

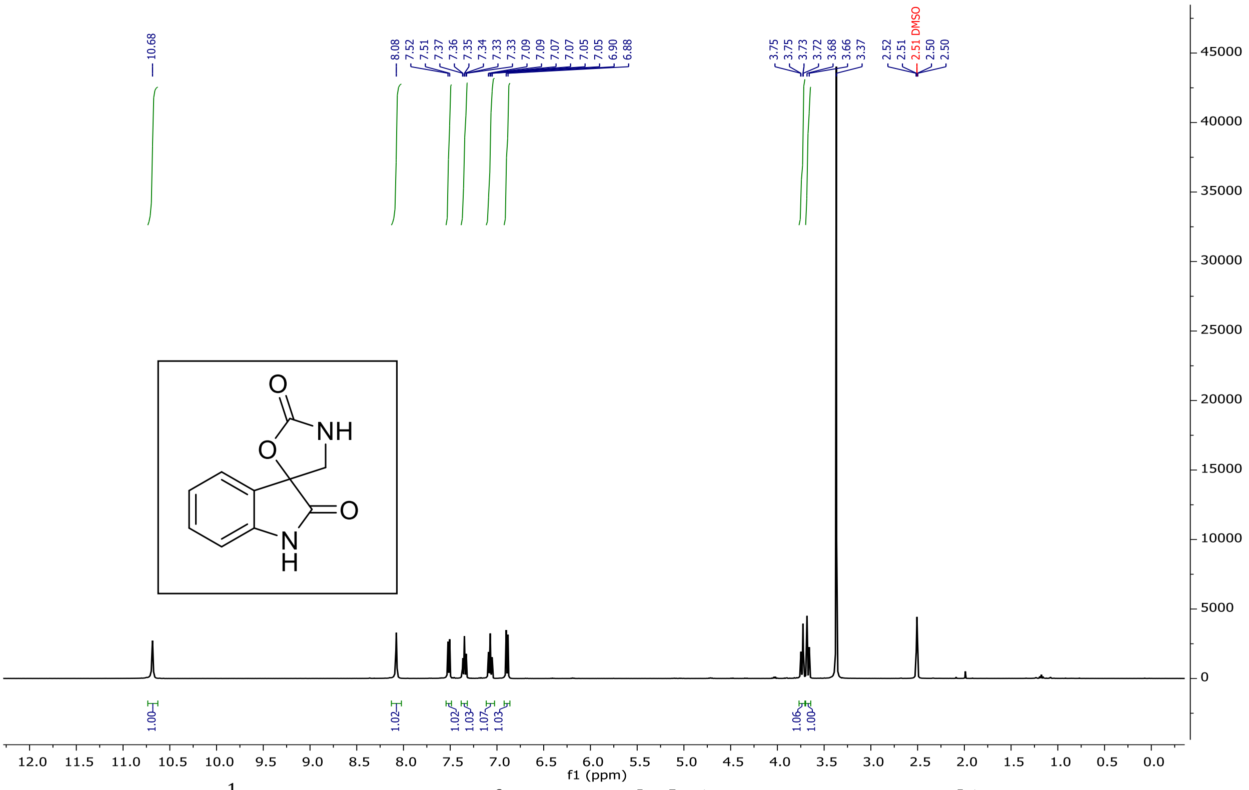

^{1}H NMR spectrum of compound **2b** (400 MHz, DMSO-d_6)

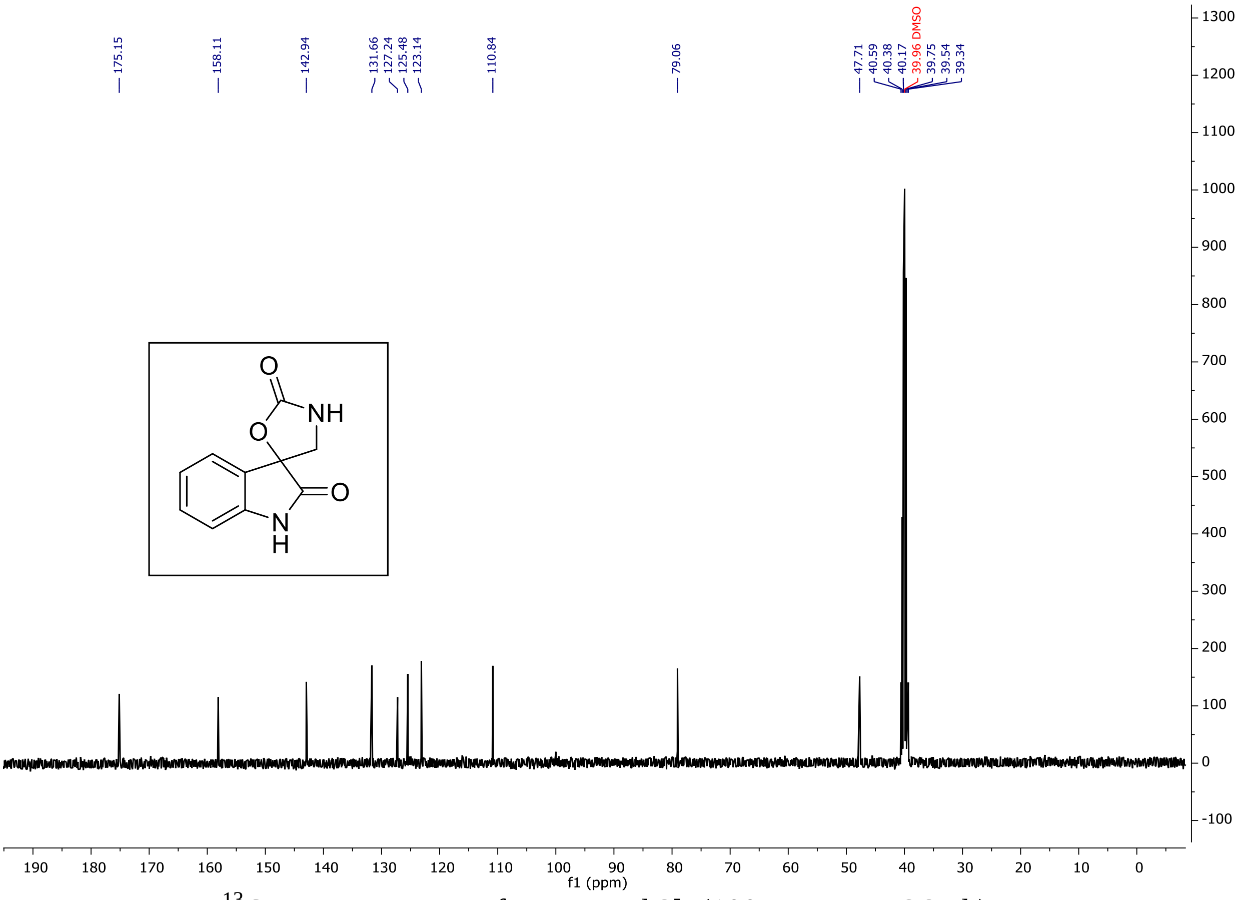

^{13}C NMR spectrum of compound **2b** (100 MHz, DMSO-d_6)

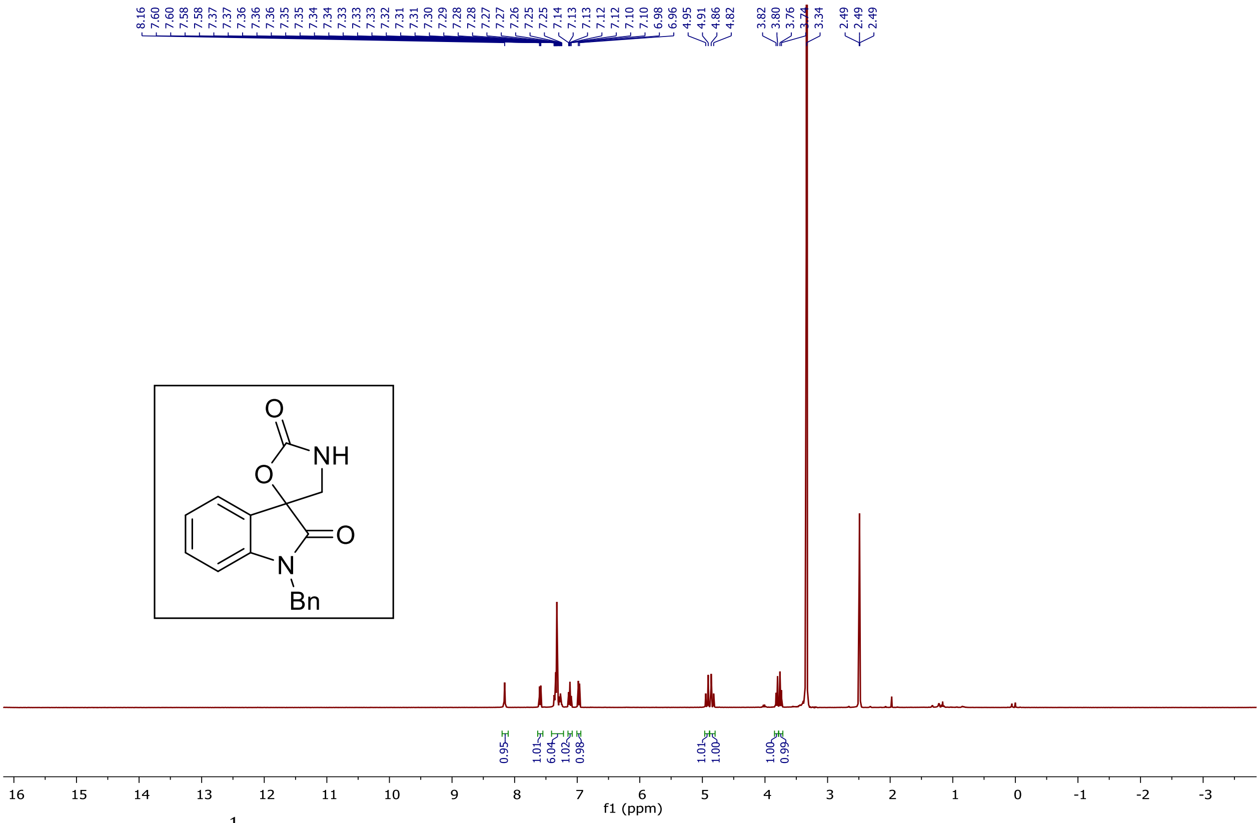

^{1}H NMR spectrum of compound **2c** (400 MHz, DMSO-d_6)

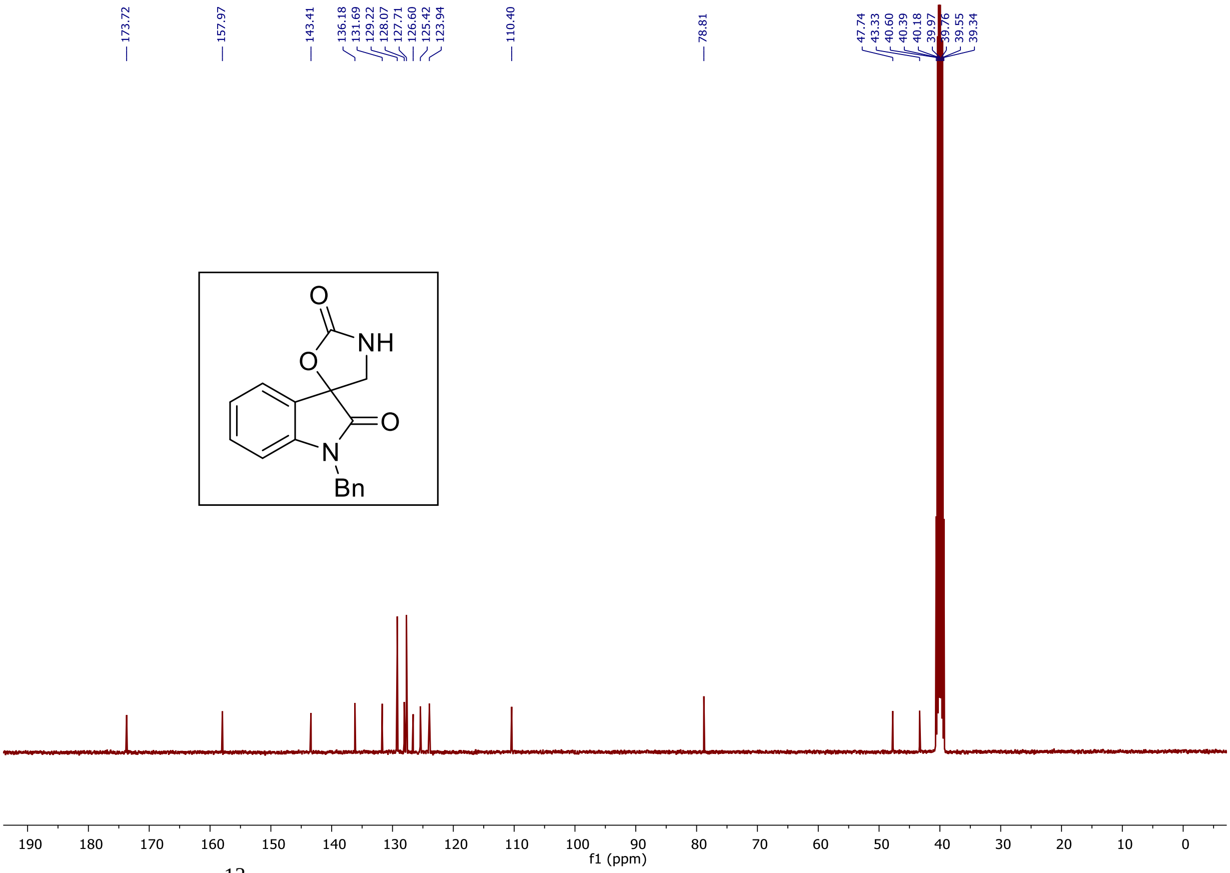

^{13}C NMR spectrum of compound **2c** (100 MHz, DMSO-d_6)

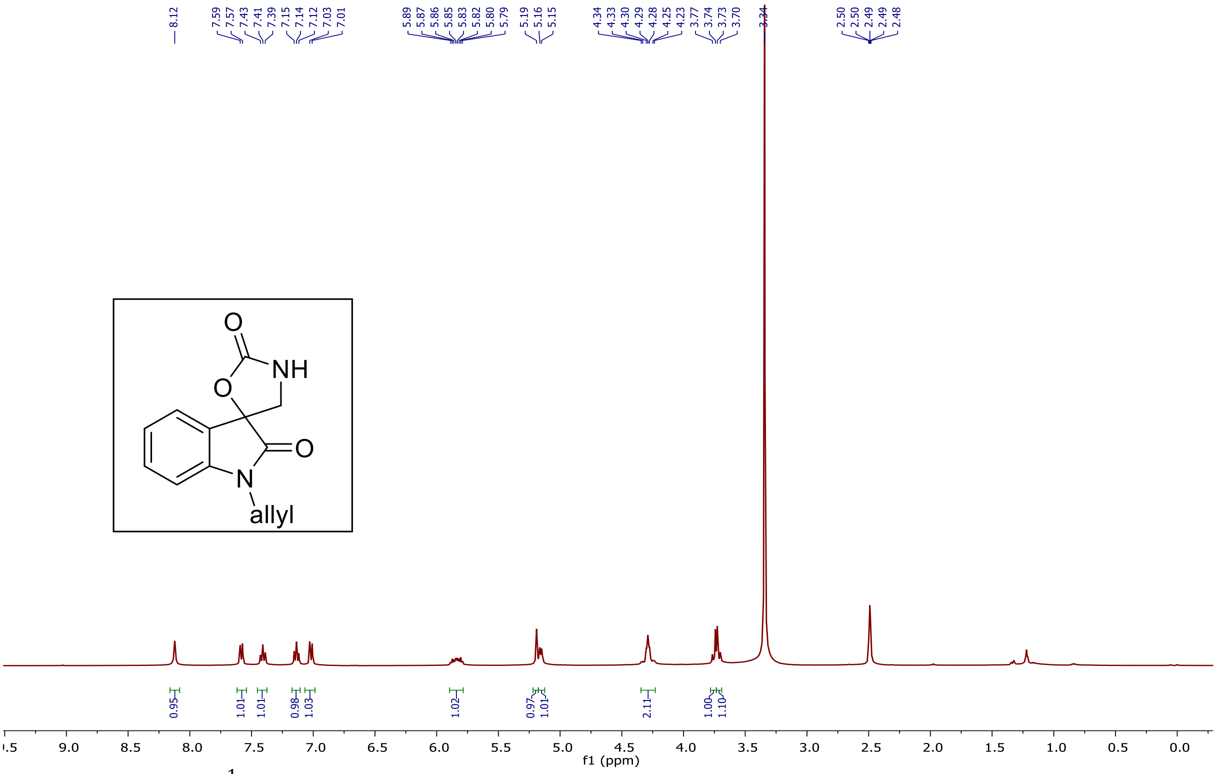

^{1}H NMR spectrum of compound **2d** (400 MHz, DMSO-d_6)

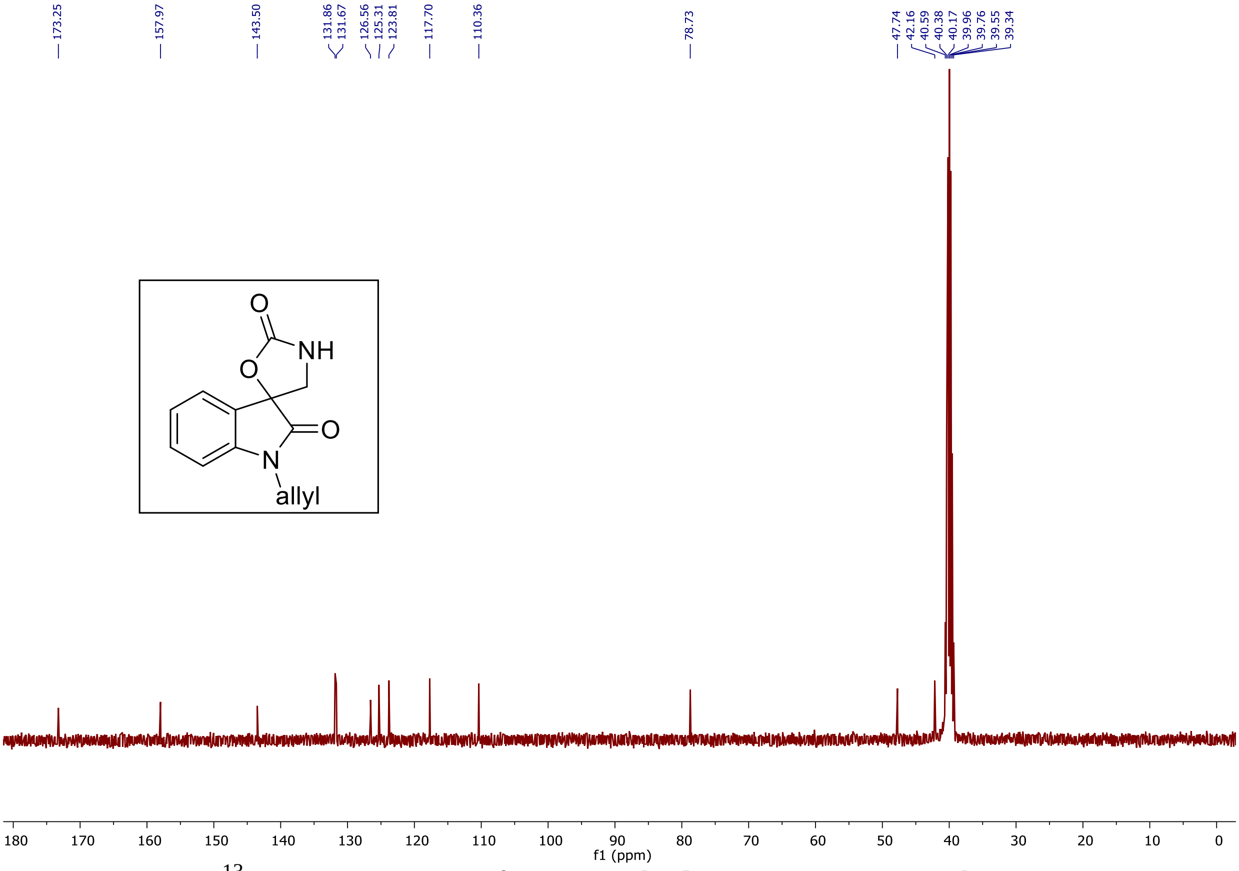

^{13}C NMR spectrum of compound **2d** (100 MHz, DMSO-d_6)

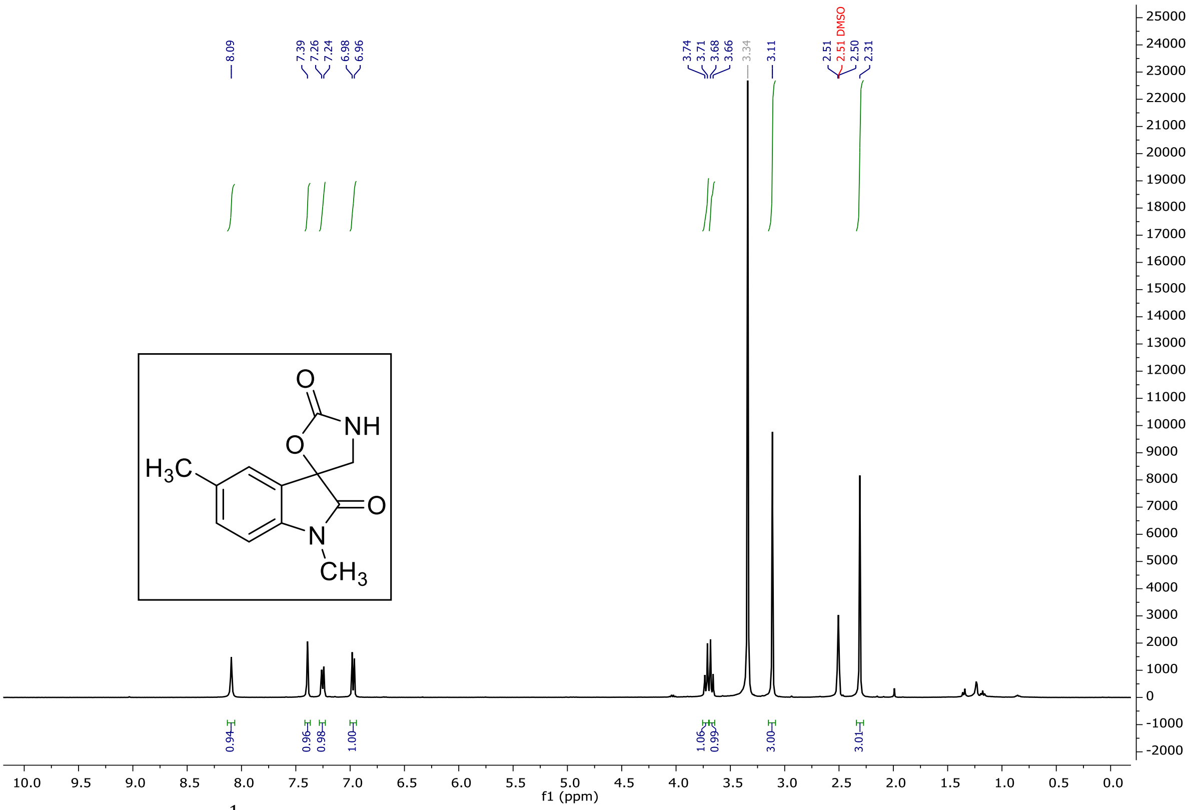

^{1}H NMR spectrum of compound **2e** (400 MHz, DMSO-d_6)

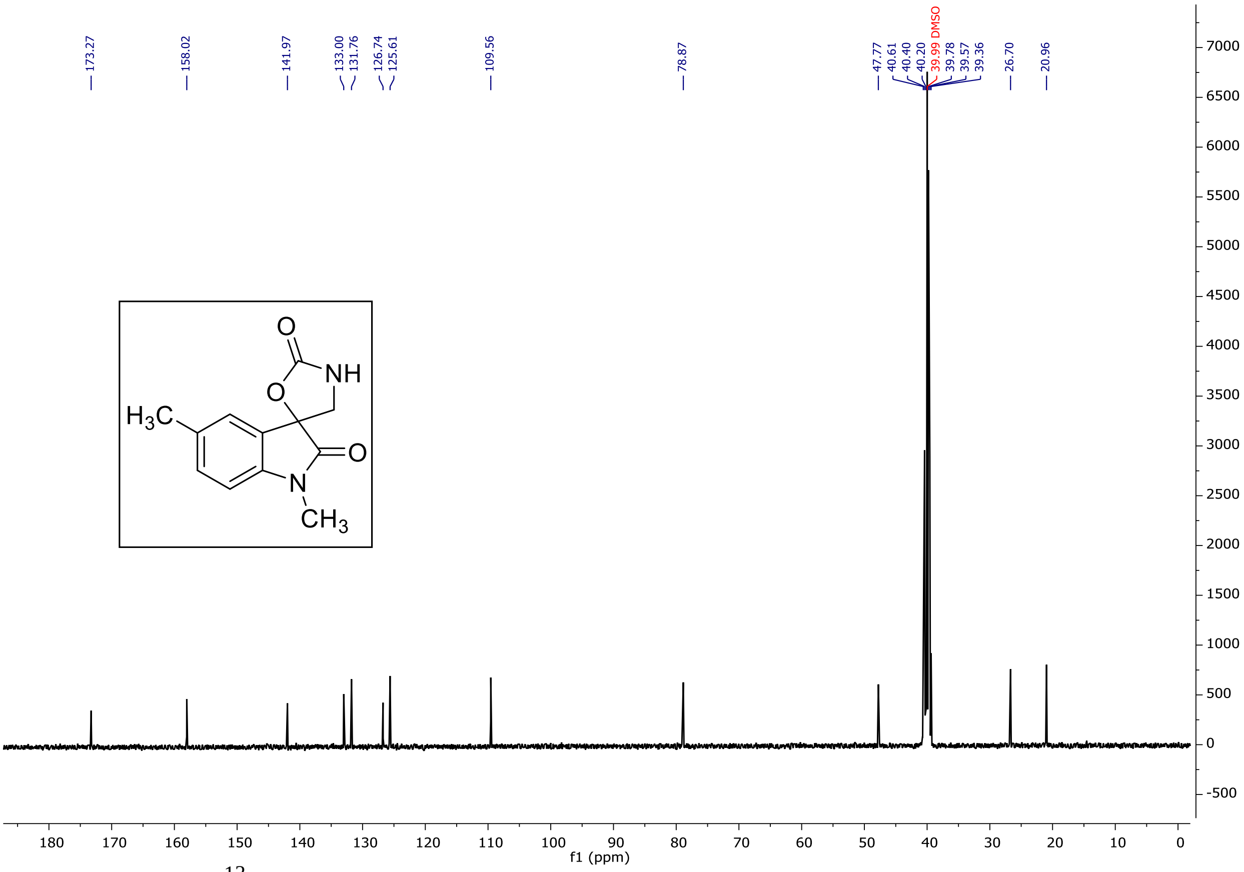

^{13}C NMR spectrum of compound **2e** (100 MHz, DMSO-d_6)

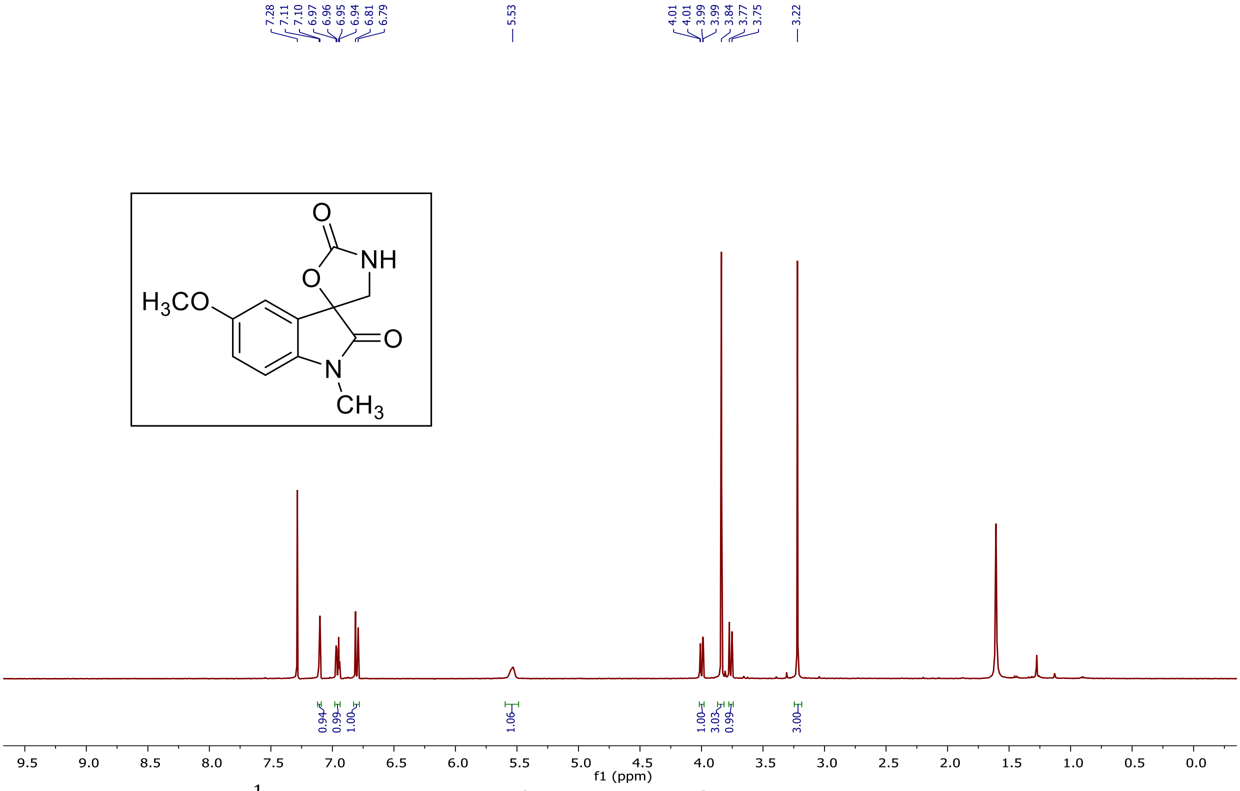

^{1}H NMR spectrum of compound **2f** (400 MHz, $CDCl_3$)

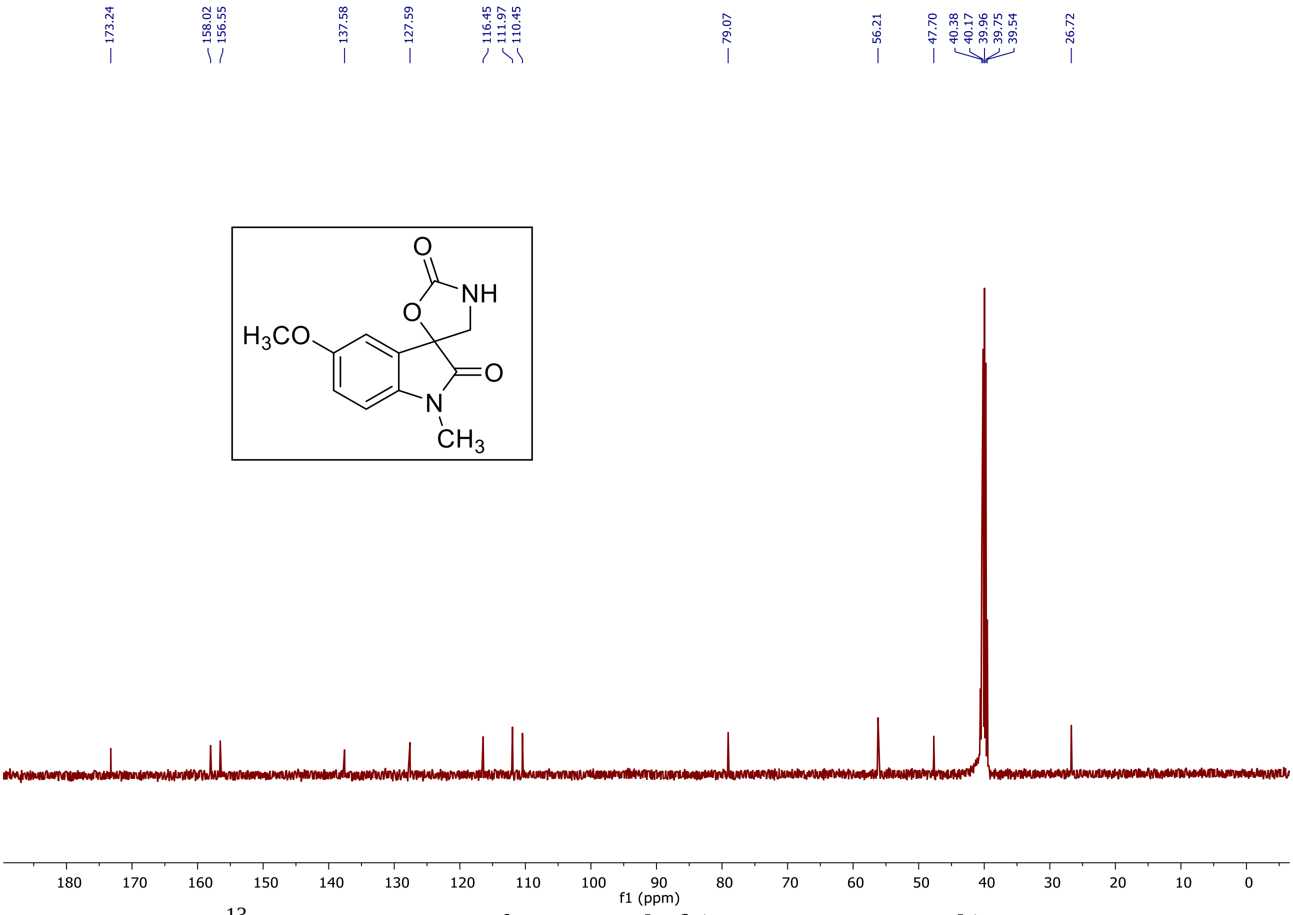

^{13}C NMR spectrum of compound **2f** (100 MHz, DMSO-d_6)

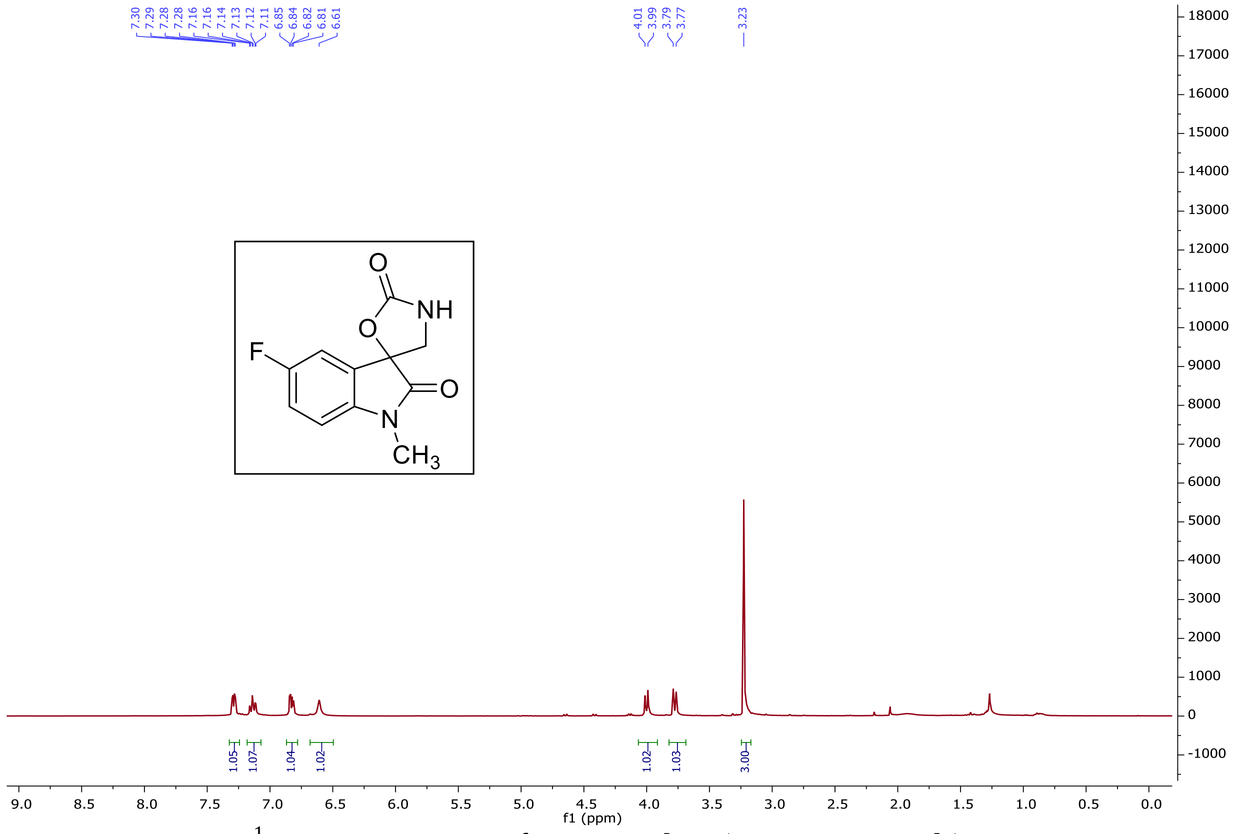

^{1}H NMR spectrum of compound **2g** (400 MHz, $CDCl_3$)

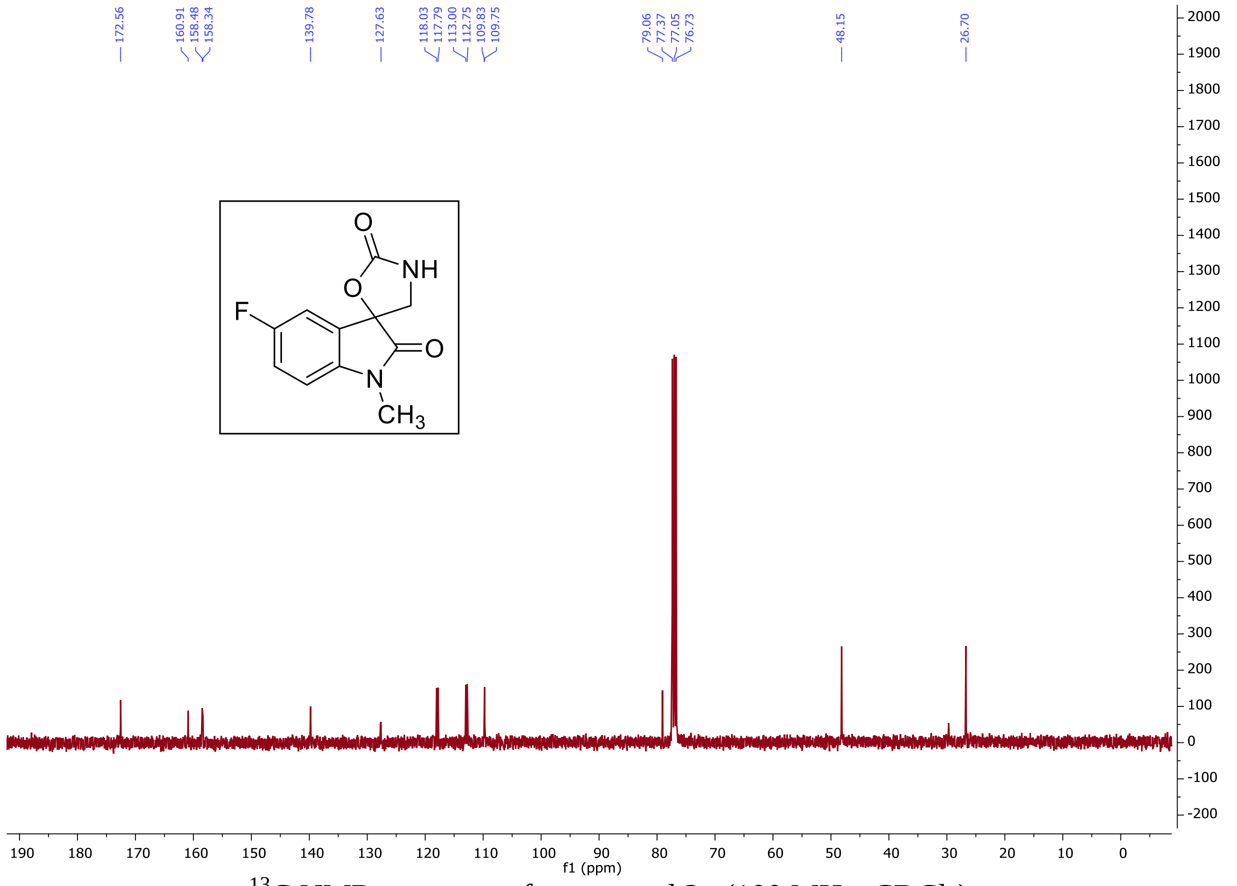

^{13}C NMR spectrum of compound **2g** (100 MHz, $CDCl_3$)

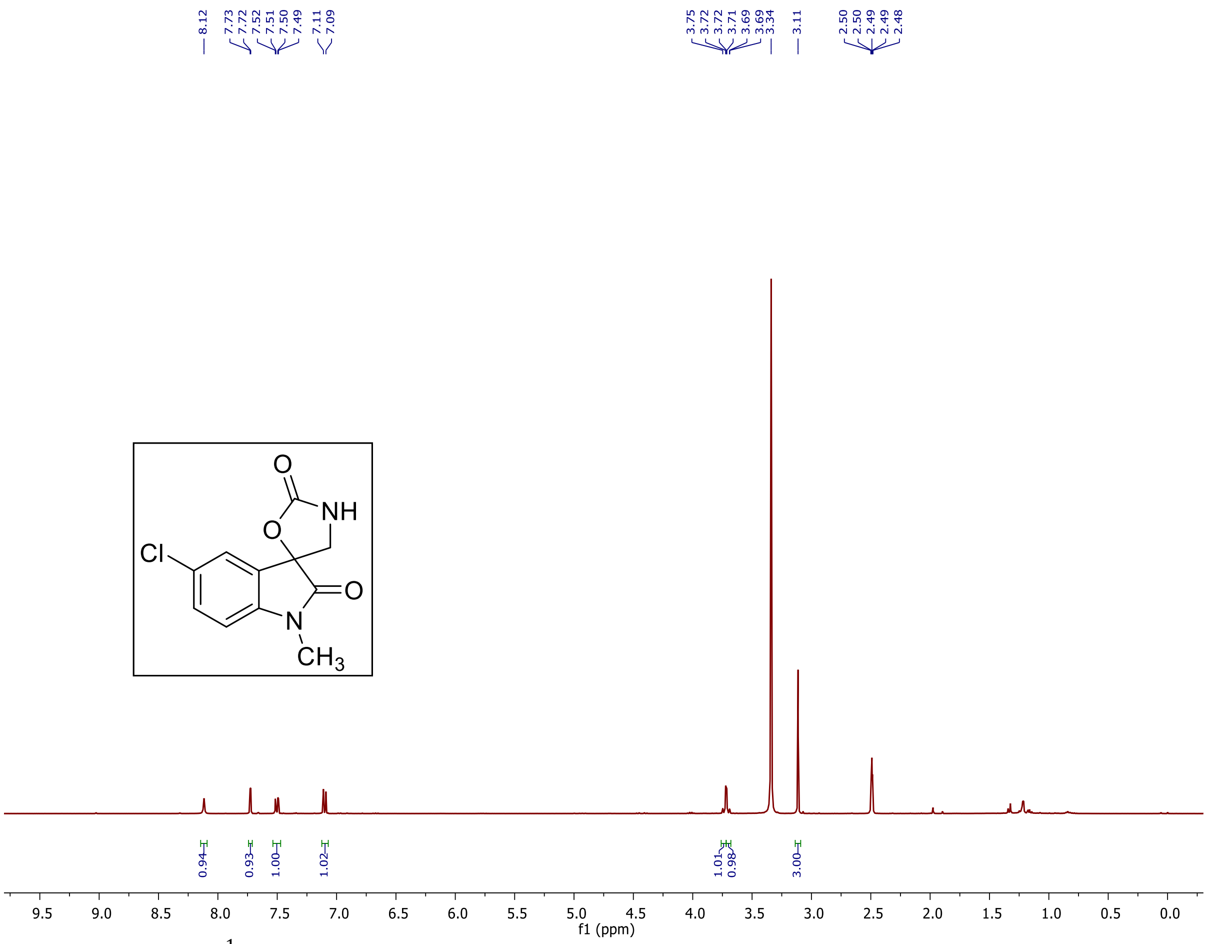

^{1}H NMR spectrum of compound **2h** (400 MHz, DMSO-d_6)

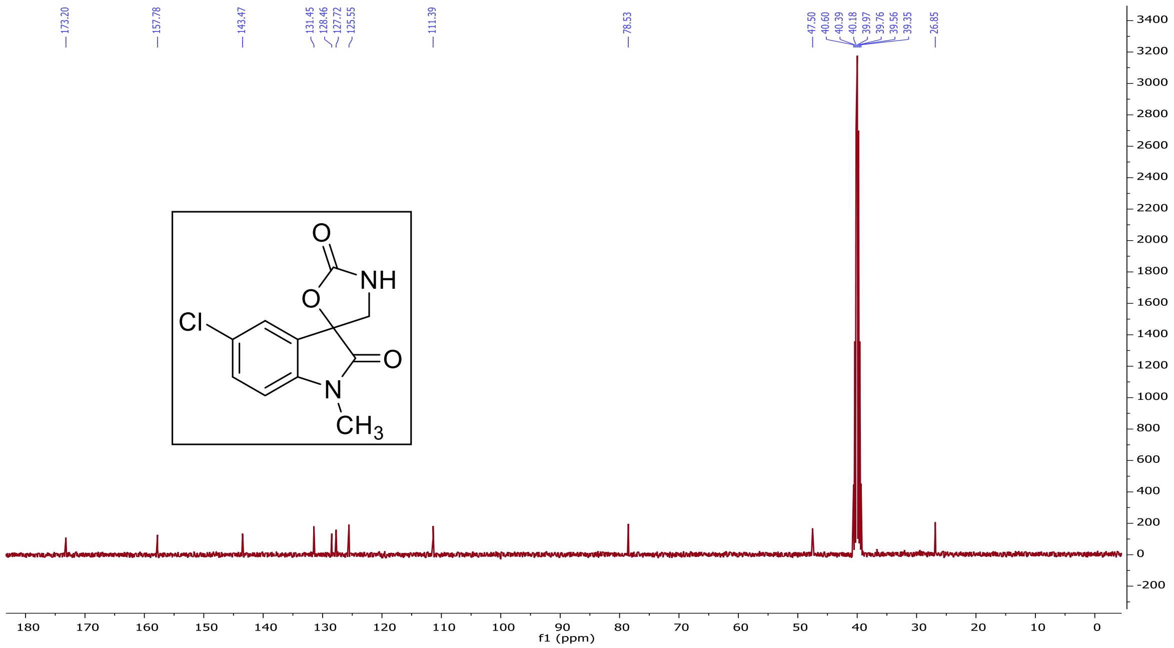

^{13}C NMR spectrum of compound **2h** (100 MHz, DMSO-d_6)

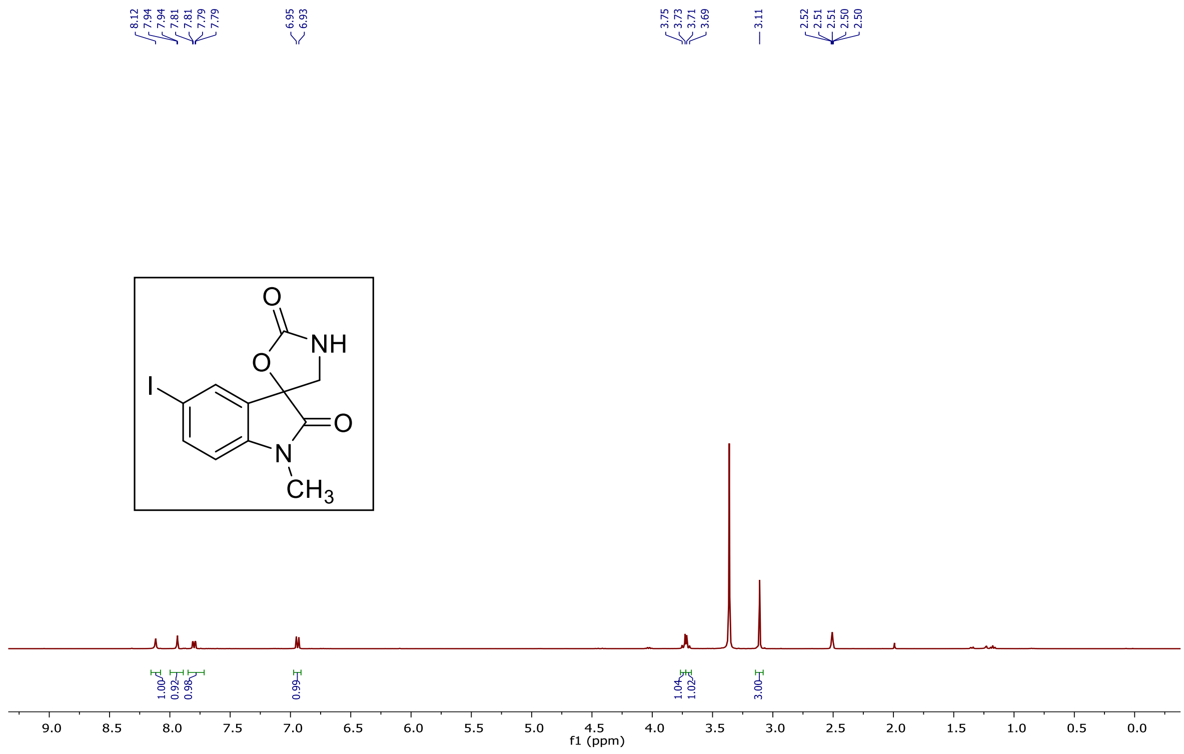

^{1}H NMR spectrum of compound **2i** (400 MHz, DMSO-d_6)

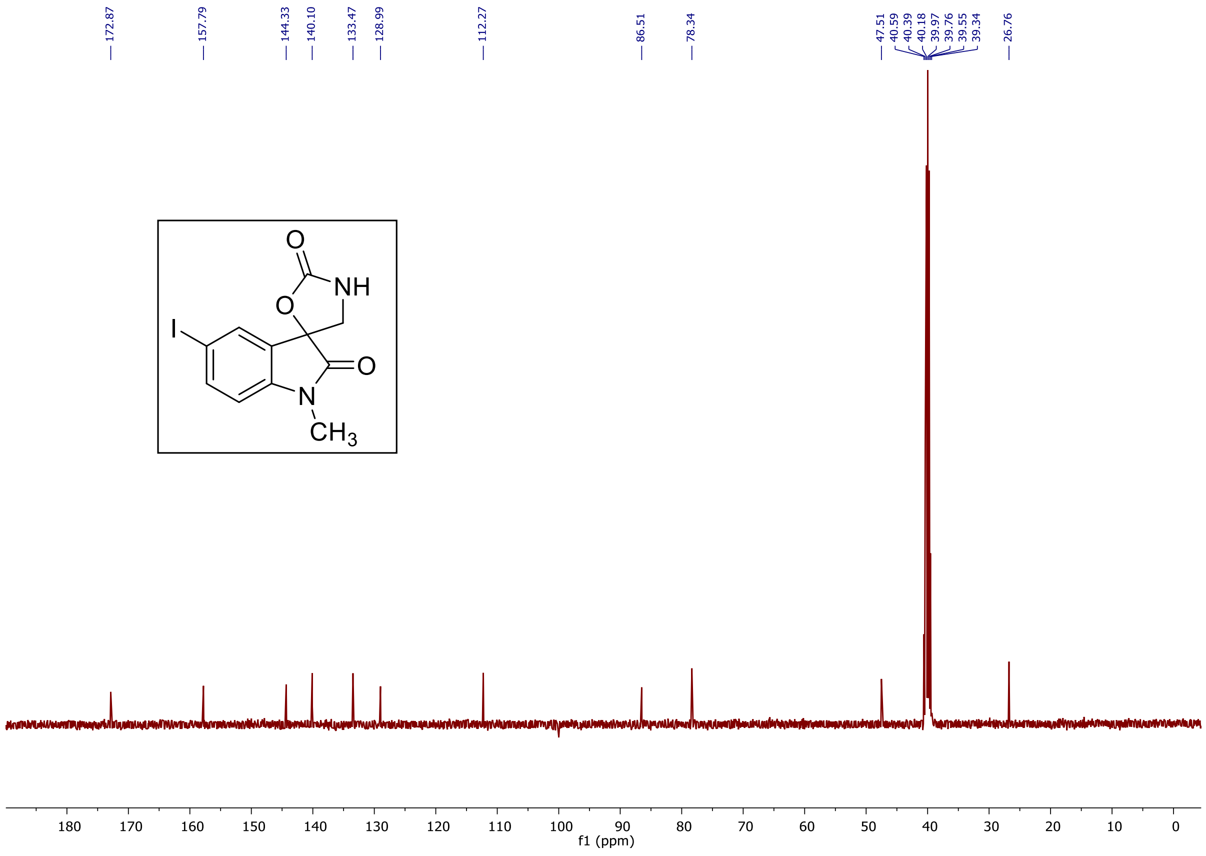

^{13}C NMR spectrum of compound **2i** (100 MHz, DMSO-d_6)

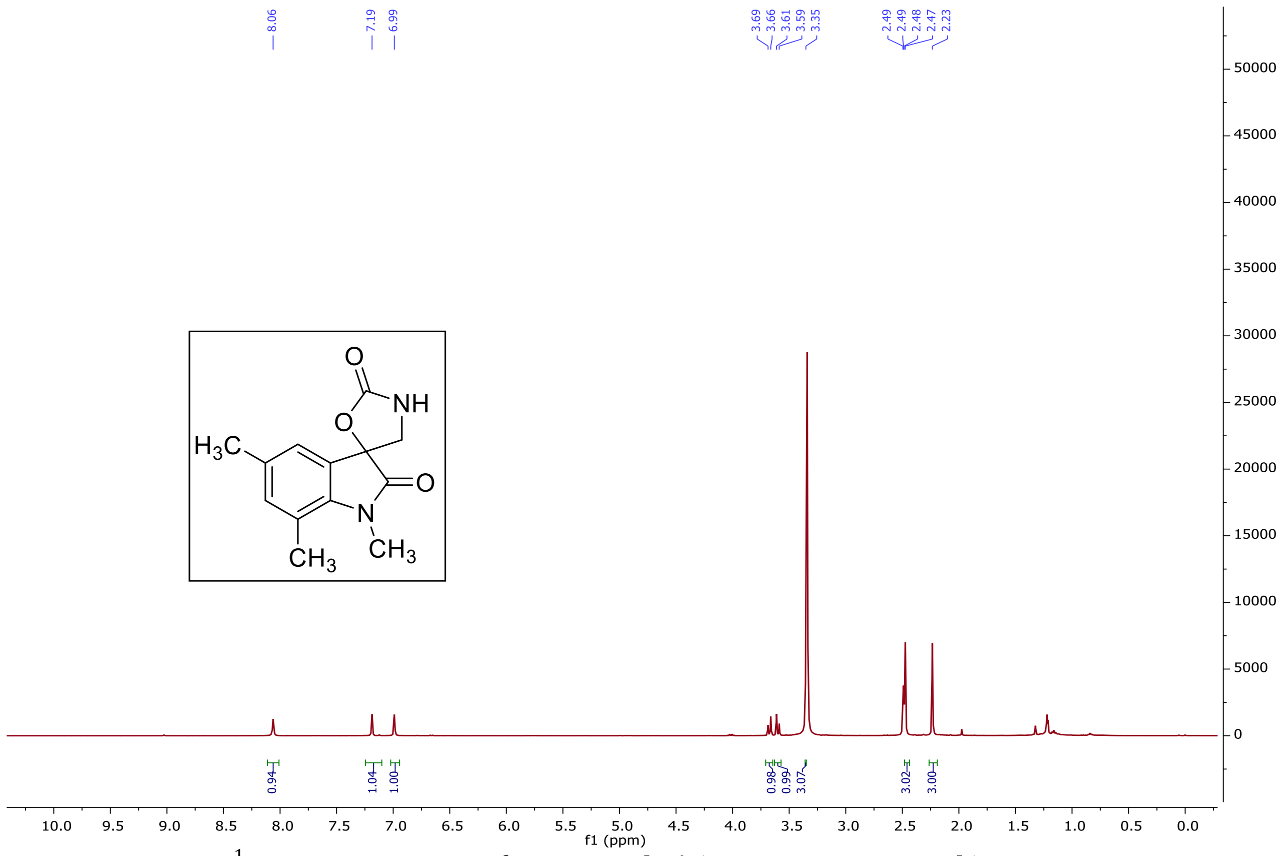

^{1}H NMR spectrum of compound **2j** (400 MHz, DMSO-d_6)

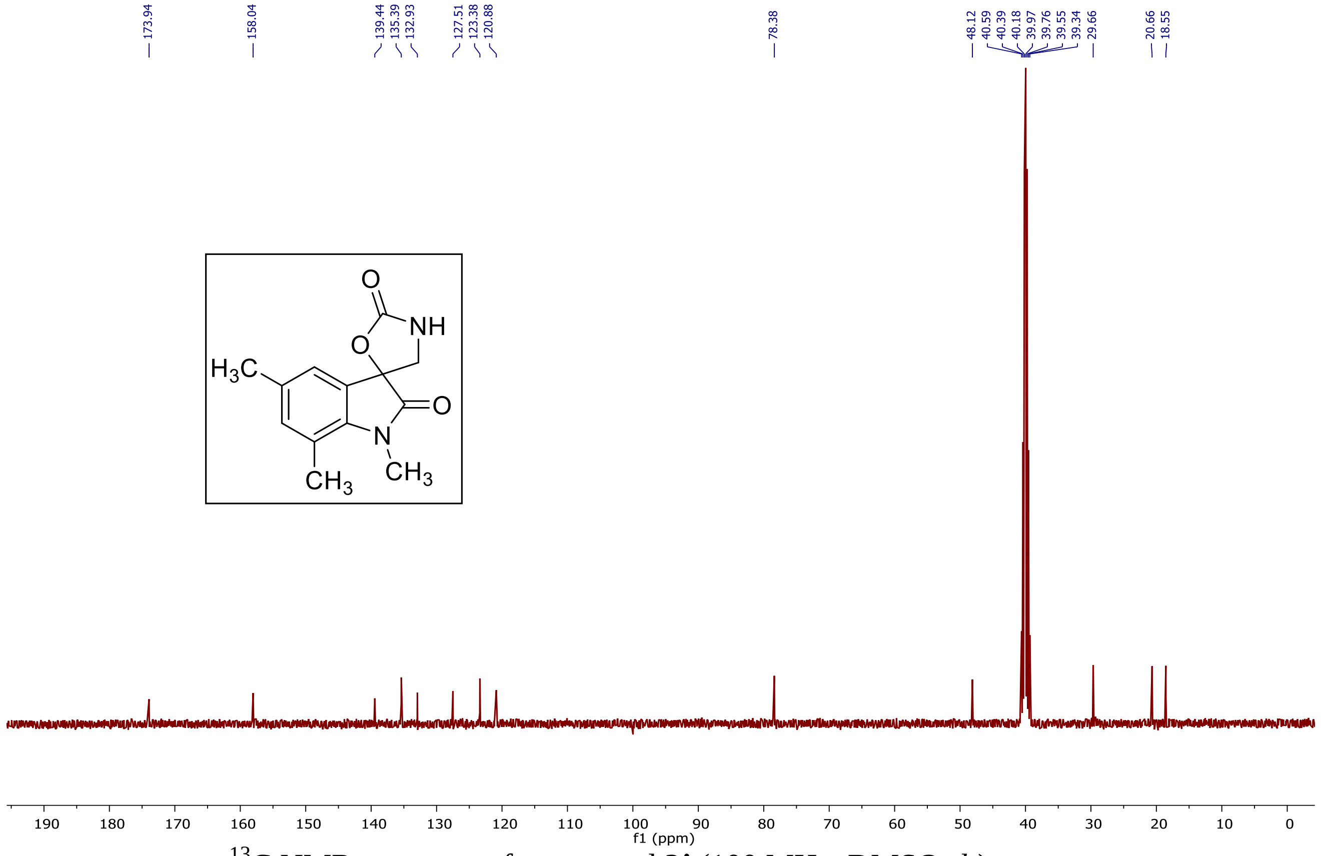

^{13}C NMR spectrum of compound **2j** (100 MHz, DMSO-d_6)

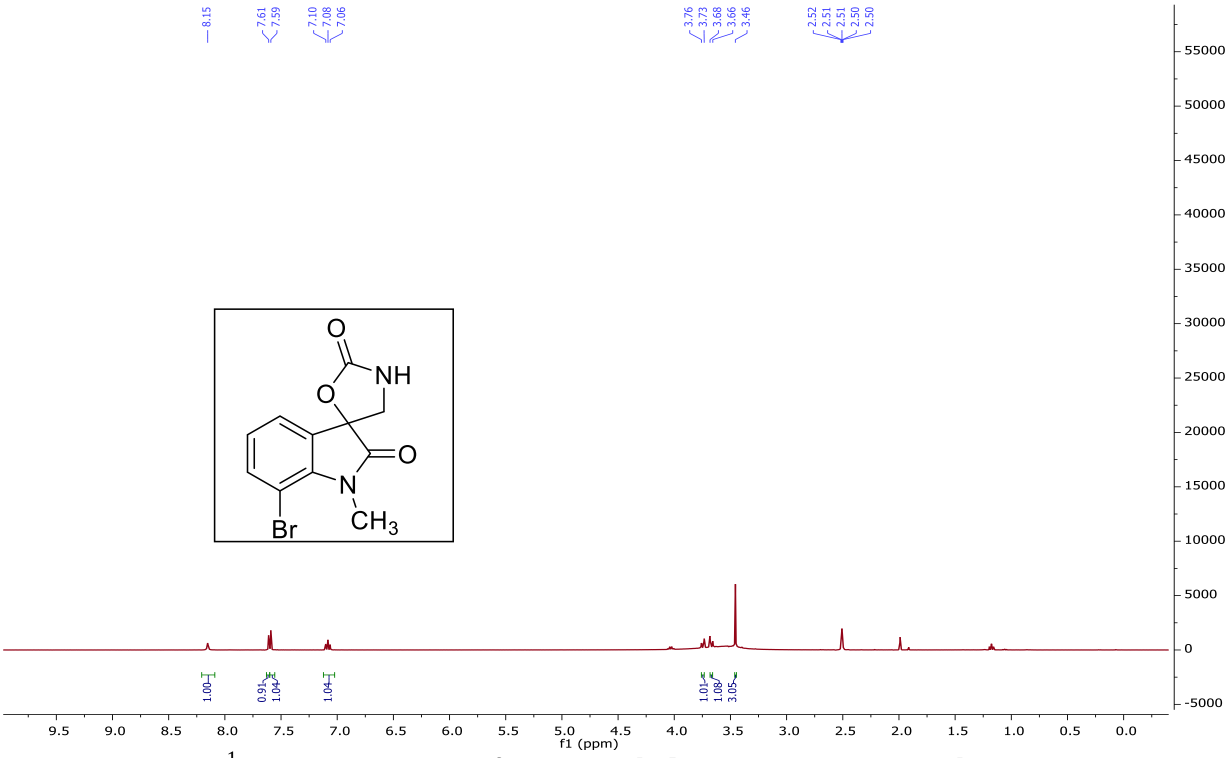

^{1}H NMR spectrum of compound **2k** (400 MHz, DMSO-d_6)

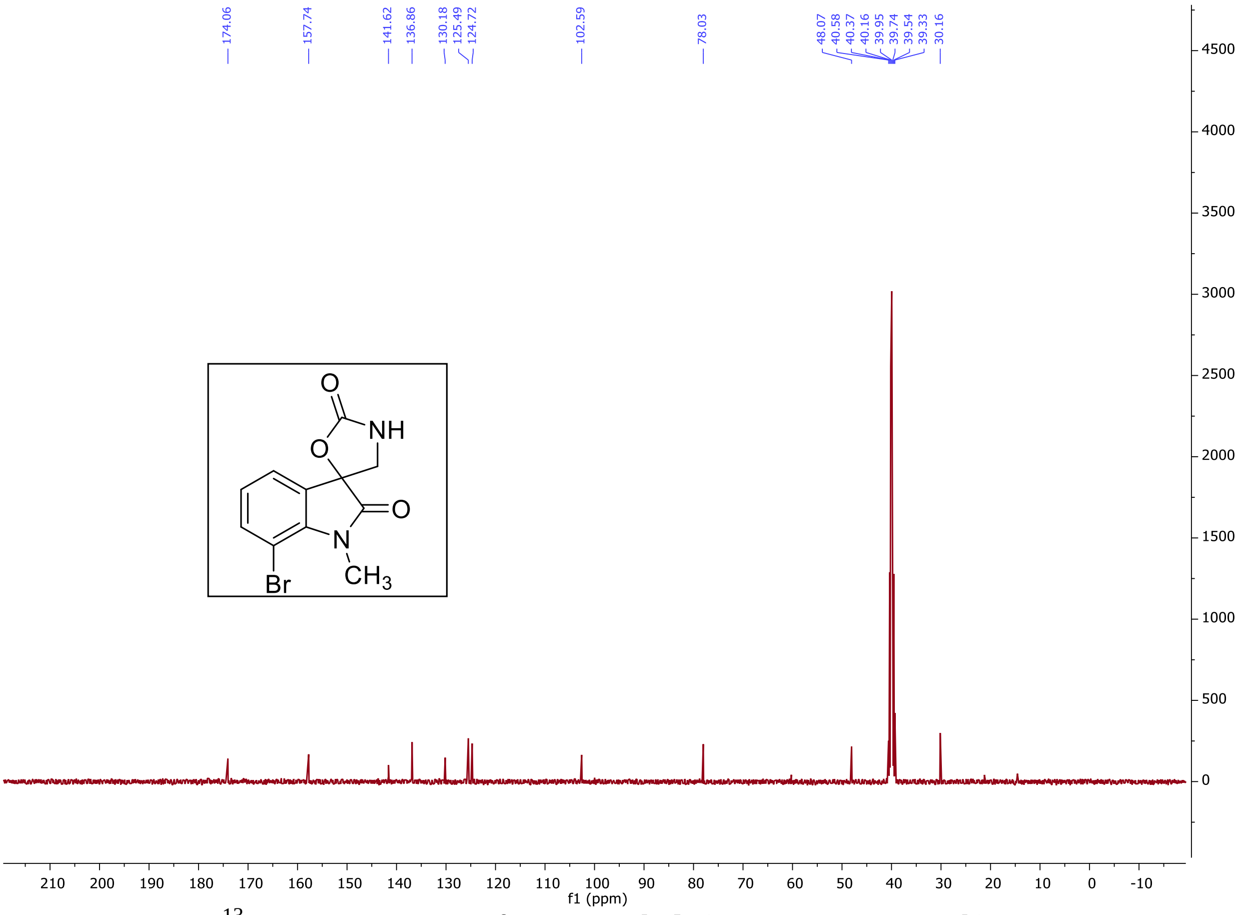

^{13}C NMR spectrum of compound **2k** (100 MHz, DMSO-d_6)

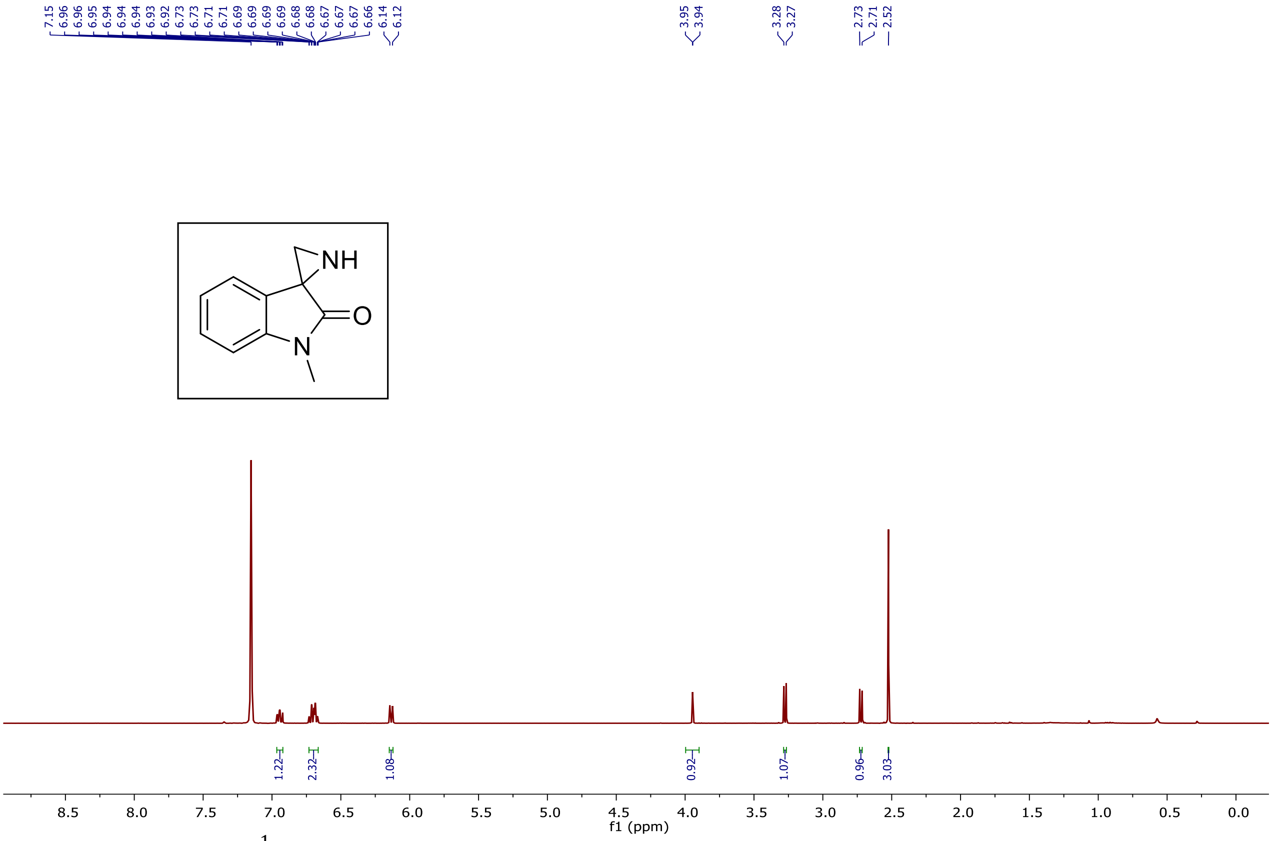

^{1}H NMR spectrum of compound **1a** (400 MHz, C_6D_6)

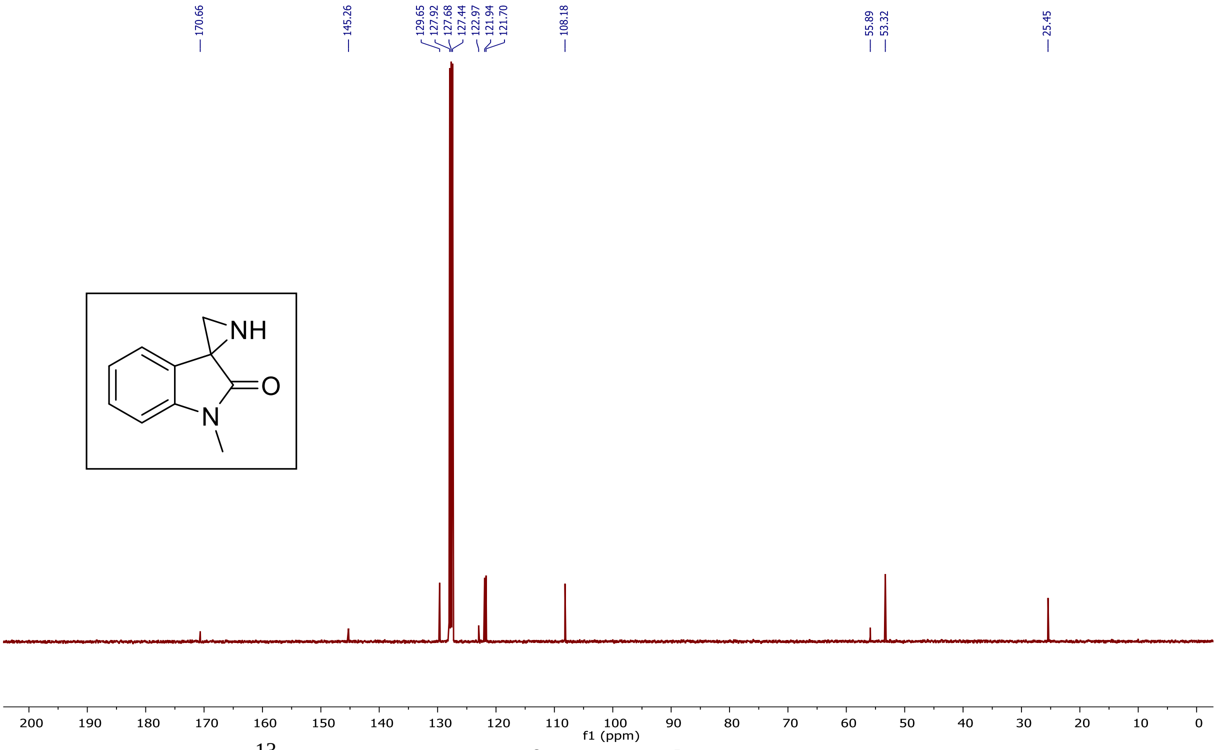

^{13}C NMR spectrum of compound **1a** (100 MHz, C_6D_6)

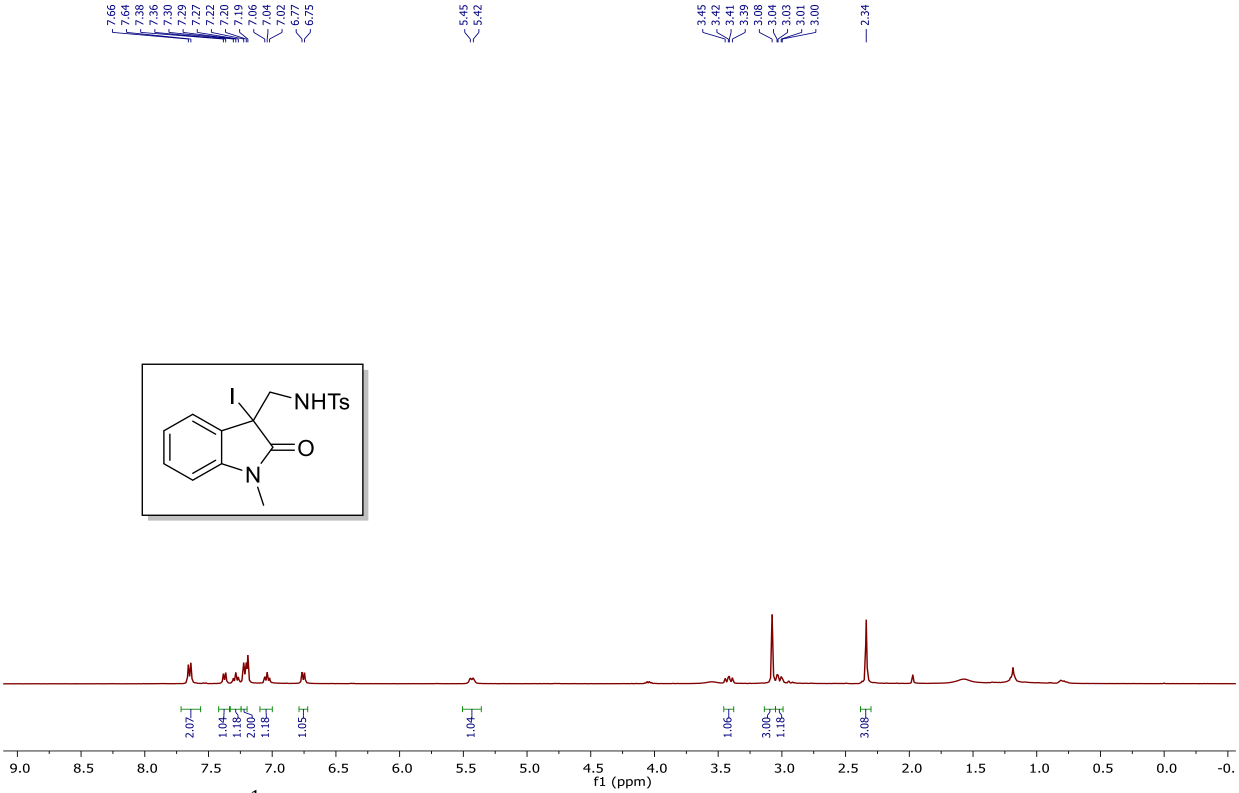

^{1}H NMR spectrum of compound **5a** (400 MHz, $CDCl_3$)

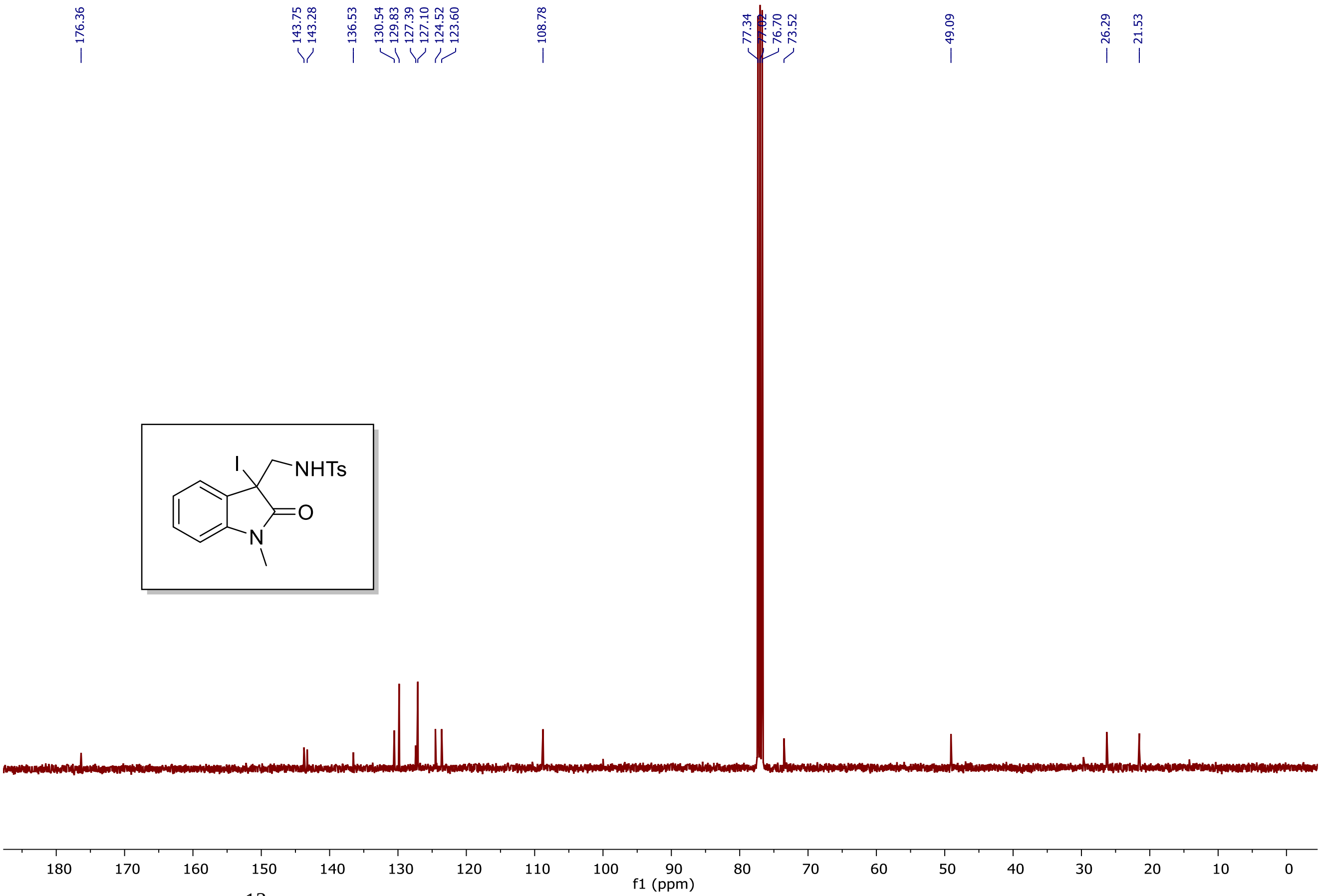

^{13}C NMR spectrum of compound **5a** (100 MHz, $CDCl_3$)

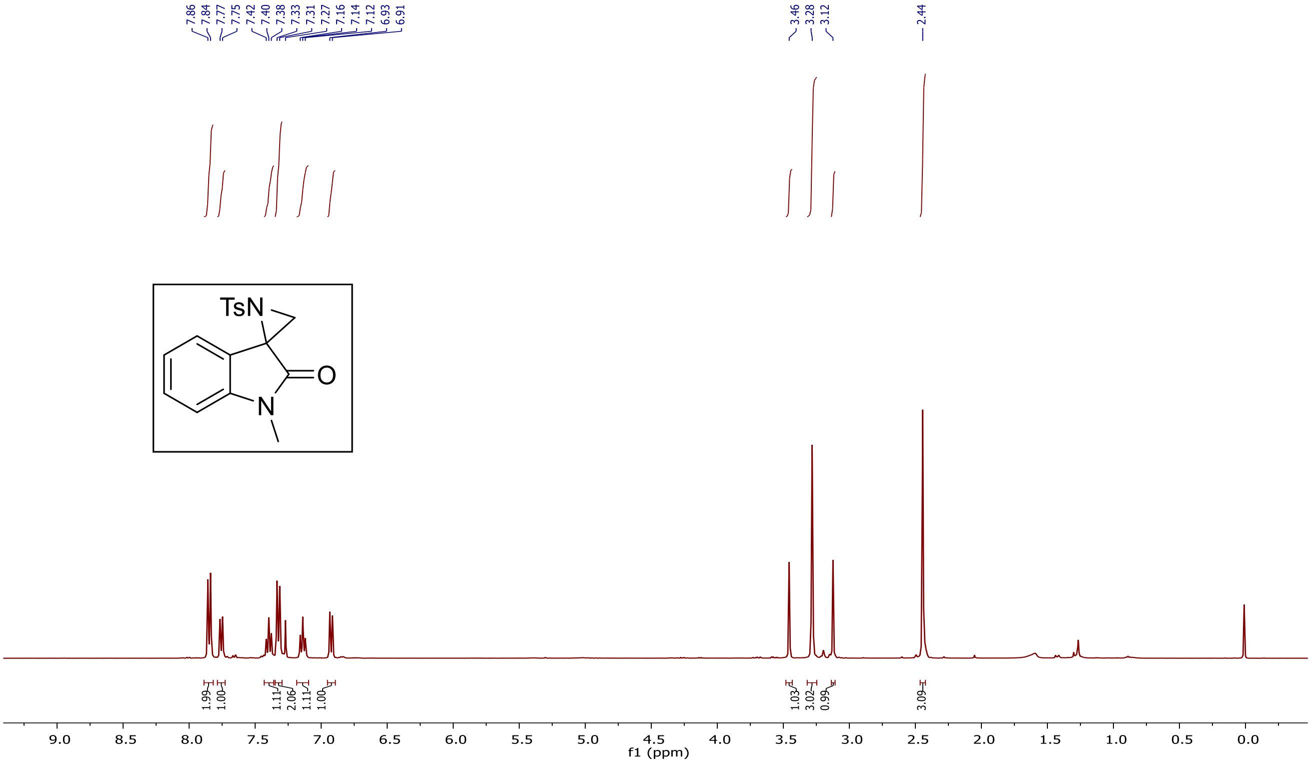

^{1}H NMR spectrum of compound **6a** (400 MHz, $CDCl_3$)

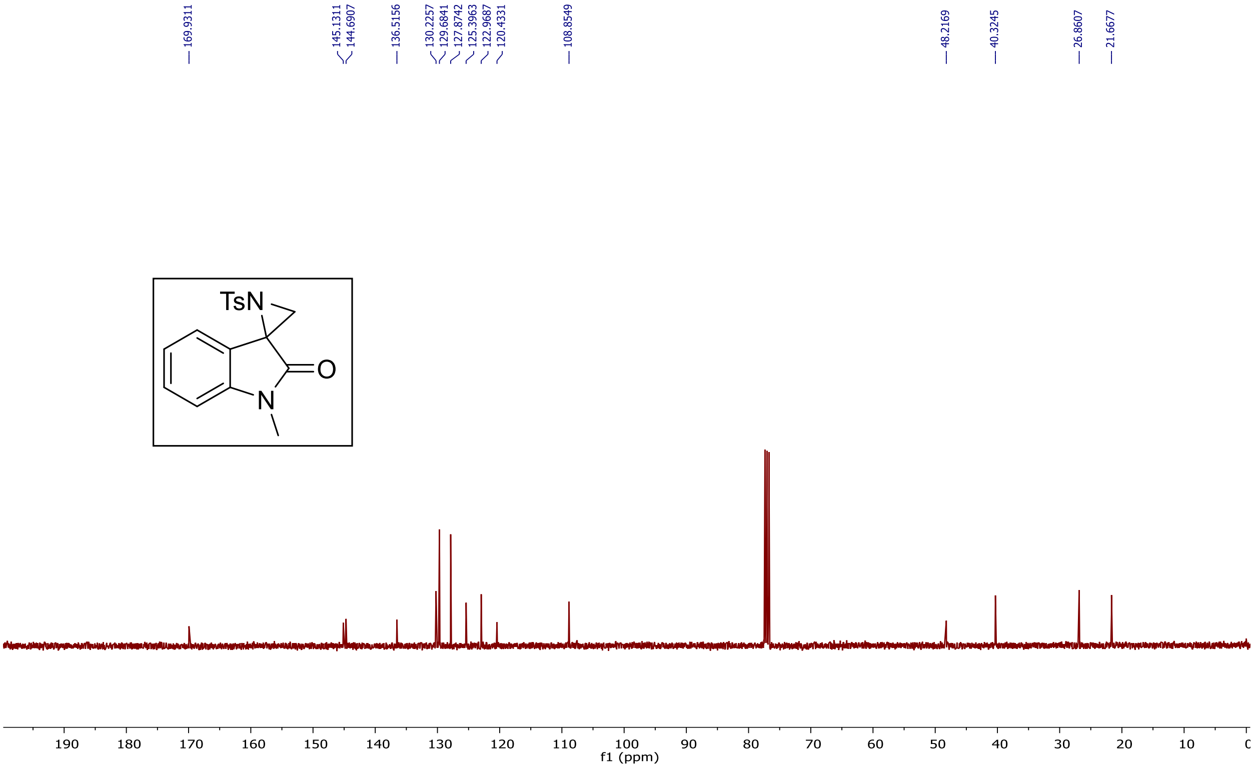

^{13}C NMR spectrum of compound **6a** (200 MHz, $CDCl_3$)
